# Supplementary material for: Spectral Signatures of Hydrogen Thioperoxide (HOSH) and Hydrogen Persulfide (HSSH): Possible Molecular Sulfur Sinks in the Dense ISM
Source: Molecules. 2022 May 17;27(10):3200. doi: 10.3390/molecules27103200 (PMC9143799; doi:10.3390/molecules27103200)
Supplement: Supplementary file 1 [file molecules-27-03200-s001.zip › molecules-1697032-supplementary.pdf]

Supporting Information for: Spectral Signatures of Hydrogen Thioperoxide (HOSH) and Hydrogen Persulfide (HSSH): Possible Molecular Sulfur Sinks in the Dense ISM

C. Zachary Palmer,[†] Ryan C. Fortenberry,[*,†] and Jospeh S. Francisco[‡]

†*Department of Chemistry and Biochemistry, University of Mississippi, University, Mississippi, United States*

‡*Department of Earth and Environmental Sciences, University of Pennsylvania, Philadelphia, Pennsylvania, United States*

E-mail: r410@olemiss.edu

Table S1: Coordinates for Calculating HOSH Dipole Components

	A	B	C
H	-0.842633611	0.001714644	-1.417667570
O	0.063234434	-0.011889422	-1.095489670
S	-0.004570772	0.045997723	0.565839045
H	-0.015725052	-1.276059704	0.808878993

Table S2: The CcCR Force Constants for HOOH

$F_{1,1}$	0.339640	$F_{1,2}$	-0.086915	$F_{1,3}$	-0.227477	$F_{1,4}$	0.008298
$F_{1,5}$	-0.015014	$F_{1,6}$	-0.007013	$F_{1,7}$	-0.346860	$F_{1,8}$	0.101582
$F_{1,9}$	0.233069	$F_{1,10}$	-0.001079	$F_{1,11}$	0.000347	$F_{1,12}$	0.001421
$F_{1,13}$	-0.086915	$F_{2,1}$	0.093007	$F_{2,2}$	0.059326	$F_{2,3}$	0.035081
$F_{2,4}$	-0.033380	$F_{2,5}$	-0.018962	$F_{2,6}$	0.051487	$F_{2,7}$	-0.054071
$F_{2,8}$	-0.037614	$F_{2,9}$	0.000347	$F_{2,10}$	-0.005556	$F_{2,11}$	-0.002750
$F_{2,12}$	-0.227477	$F_{2,13}$	0.059326	$F_{3,1}$	0.156259	$F_{3,2}$	-0.004265
$F_{3,3}$	0.004660	$F_{3,4}$	0.001213	$F_{3,5}$	0.233163	$F_{3,6}$	-0.066736
$F_{3,7}$	-0.158782	$F_{3,8}$	-0.001421	$F_{3,9}$	0.002750	$F_{3,10}$	0.001311
$F_{3,11}$	0.008298	$F_{3,12}$	0.035081	$F_{3,13}$	-0.004265	$F_{4,1}$	0.392539
$F_{4,2}$	-0.087947	$F_{4,3}$	0.238755	$F_{4,4}$	-0.053978	$F_{4,5}$	0.001379
$F_{4,6}$	-0.001327	$F_{4,7}$	-0.346860	$F_{4,8}$	0.051487	$F_{4,9}$	-0.233163
$F_{4,10}$	-0.015014	$F_{4,11}$	-0.033380	$F_{4,12}$	0.004660	$F_{4,13}$	-0.087947
$F_{5,1}$	0.315174	$F_{5,2}$	-0.045024	$F_{5,3}$	0.001379	$F_{5,4}$	-0.227724
$F_{5,5}$	-0.026372	$F_{5,6}$	0.101582	$F_{5,7}$	-0.054071	$F_{5,8}$	0.066736
$F_{5,9}$	-0.007013	$F_{5,10}$	-0.018962	$F_{5,11}$	0.001213	$F_{5,12}$	0.238755
$F_{5,13}$	-0.045024	$F_{6,1}$	0.177890	$F_{6,2}$	0.001327	$F_{6,3}$	0.026372
$F_{6,4}$	-0.020321	$F_{6,5}$	-0.233069	$F_{6,6}$	0.037614	$F_{6,7}$	-0.158782
$F_{6,8}$	-0.346860	$F_{6,9}$	0.051487	$F_{6,10}$	0.233163	$F_{6,11}$	-0.053978
$F_{6,12}$	0.001379	$F_{6,13}$	0.001327	$F_{7,1}$	0.392539	$F_{7,2}$	-0.087947
$F_{7,3}$	-0.238755	$F_{7,4}$	0.008298	$F_{7,5}$	0.035081	$F_{7,6}$	0.004265
$F_{7,7}$	0.101582	$F_{7,8}$	-0.054071	$F_{7,9}$	-0.066736	$F_{7,10}$	0.001379
$F_{7,11}$	-0.227724	$F_{7,12}$	0.026372	$F_{7,13}$	-0.087947	$F_{8,1}$	0.315174
$F_{8,2}$	0.045024	$F_{8,3}$	-0.015014	$F_{8,4}$	-0.033380	$F_{8,5}$	-0.004660
$F_{8,6}$	0.233069	$F_{8,7}$	-0.037614	$F_{8,8}$	-0.158782	$F_{8,9}$	-0.001327
$F_{8,10}$	-0.026372	$F_{8,11}$	-0.020321	$F_{8,12}$	-0.238755	$F_{8,13}$	0.045024
$F_{9,1}$	0.177890	$F_{9,2}$	0.007013	$F_{9,3}$	0.018962	$F_{9,4}$	0.001213
$F_{9,5}$	-0.001079	$F_{9,6}$	0.000347	$F_{9,7}$	-0.001421	$F_{9,8}$	-0.346860
$F_{9,9}$	0.101582	$F_{9,10}$	-0.233069	$F_{9,11}$	0.008298	$F_{9,12}$	-0.015014
$F_{9,13}$	0.007013	$F_{10,1}$	0.339640	$F_{10,2}$	-0.086915	$F_{10,3}$	0.227477
$F_{10,4}$	0.000347	$F_{10,5}$	-0.005556	$F_{10,6}$	0.002750	$F_{10,7}$	0.051487
$F_{10,8}$	-0.054071	$F_{10,9}$	0.037614	$F_{10,10}$	0.035081	$F_{10,11}$	-0.033380
$F_{10,12}$	0.018962	$F_{10,13}$	-0.086915	$F_{11,1}$	0.093007	$F_{11,2}$	-0.059326
$F_{11,3}$	0.001421	$F_{11,4}$	-0.002750	$F_{11,5}$	0.001311	$F_{11,6}$	-0.233163
$F_{11,7}$	0.066736	$F_{11,8}$	-0.158782	$F_{11,9}$	0.004265	$F_{11,10}$	-0.004660
$F_{11,11}$	0.001213	$F_{11,12}$	0.227477	$F_{11,13}$	-0.059326	$F_{12,1}$	0.156259
$F_{1,1,1}$	-0.7736	$F_{1,1,2}$	0.3477	$F_{1,2,2}$	0.0480	$F_{2,2,2}$	-0.0894
$F_{1,1,3}$	0.8345	$F_{1,2,3}$	-0.2802	$F_{2,2,3}$	-0.0292	$F_{1,3,3}$	-0.4433
$F_{2,3,3}$	0.1306	$F_{3,3,3}$	-0.0095	$F_{1,1,4}$	-0.0030	$F_{1,2,4}$	-0.0085
$F_{2,2,4}$	0.0171	$F_{1,3,4}$	0.0055	$F_{2,3,4}$	0.0265	$F_{3,3,4}$	-0.0019
$F_{1,4,4}$	-0.0011	$F_{2,4,4}$	-0.0080	$F_{3,4,4}$	0.0175	$F_{4,4,4}$	-0.7921
$F_{1,1,5}$	0.0230	$F_{1,2,5}$	0.0002	$F_{2,2,5}$	-0.0713	$F_{1,3,5}$	-0.0200
$F_{2,3,5}$	0.0016	$F_{3,3,5}$	0.0034	$F_{1,4,5}$	-0.0098	$F_{2,4,5}$	-0.0377
$F_{3,4,5}$	-0.0034	$F_{4,4,5}$	0.3696	$F_{1,5,5}$	0.0047	$F_{2,5,5}$	0.0844
$F_{3,5,5}$	0.0094	$F_{4,5,5}$	0.0188	$F_{5,5,5}$	-1.0407	$F_{1,1,6}$	0.0084
$F_{1,2,6}$	0.0211	$F_{2,2,6}$	-0.0173	$F_{1,3,6}$	0.0017	$F_{2,3,6}$	0.0096
$F_{3,3,6}$	-0.0064	$F_{1,4,6}$	0.0064	$F_{2,4,6}$	-0.0074	$F_{3,4,6}$	0.0065
$F_{4,4,6}$	-0.8842	$F_{1,5,6}$	0.0015	$F_{2,5,6}$	0.0245	$F_{3,5,6}$	-0.0129
$F_{4,5,6}$	0.2618	$F_{5,5,6}$	-0.0152	$F_{1,6,6}$	-0.0132	$F_{2,6,6}$	-0.0080
$F_{3,6,6}$	-0.0095	$F_{4,6,6}$	-0.4559	$F_{5,6,6}$	0.2010	$F_{6,6,6}$	0.0572
$F_{1,1,7}$	0.7745	$F_{1,2,7}$	-0.3390	$F_{2,2,7}$	-0.0663	$F_{1,3,7}$	-0.8408
$F_{2,3,7}$	0.2550	$F_{3,3,7}$	0.4456	$F_{1,4,7}$	0.0013	$F_{2,4,7}$	0.0184
$F_{3,4,7}$	-0.0234	$F_{4,4,7}$	0.0201	$F_{1,5,7}$	-0.0124	$F_{2,5,7}$	0.0387
$F_{3,5,7}$	0.0197	$F_{4,5,7}$	-0.0103	$F_{5,5,7}$	0.0283	$F_{1,6,7}$	-0.0140
$F_{2,6,7}$	-0.0170	$F_{3,6,7}$	-0.0064	$F_{4,6,7}$	0.0234	$F_{5,6,7}$	0.0197
$F_{6,6,7}$	0.0121	$F_{1,7,7}$	-0.7731	$F_{2,7,7}$	0.3193	$F_{3,7,7}$	0.8658
$F_{4,7,7}$	-0.0201	$F_{5,7,7}$	0.0422	$F_{6,7,7}$	0.0009	$F_{7,7,7}$	0.7921
$F_{1,1,8}$	-0.3699	$F_{1,2,8}$	-0.0488	$F_{2,2,8}$	0.1622	$F_{1,3,8}$	0.3029
$F_{2,3,8}$	0.0330	$F_{3,3,8}$	-0.1359	$F_{1,4,8}$	0.0195	$F_{2,4,8}$	0.0214
$F_{3,4,8}$	-0.0201	$F_{4,4,8}$	-0.0422	$F_{1,5,8}$	-0.0042	$F_{2,5,8}$	-0.0144
$F_{3,5,8}$	-0.0091	$F_{4,5,8}$	0.0456	$F_{5,5,8}$	0.8099	$F_{1,6,8}$	-0.0235
$F_{2,6,8}$	-0.0126	$F_{3,6,8}$	0.0033	$F_{4,6,8}$	-0.0194	$F_{5,6,8}$	0.0091
$F_{6,6,8}$	-0.0493	$F_{1,7,8}$	0.3495	$F_{2,7,8}$	0.0267	$F_{3,7,8}$	-0.2811
$F_{4,7,8}$	0.0103	$F_{5,7,8}$	-0.0456	$F_{6,7,8}$	0.0227	$F_{7,7,8}$	-0.3696
$F_{1,8,8}$	0.0518	$F_{2,8,8}$	-0.1465	$F_{3,8,8}$	-0.0294	$F_{4,8,8}$	-0.0283
$F_{5,8,8}$	-0.8099	$F_{6,8,8}$	0.0351	$F_{7,8,8}$	-0.0188	$F_{8,8,8}$	1.0407
$F_{1,1,9}$	-0.8435	$F_{1,2,9}$	0.2596	$F_{2,2,9}$	0.0447	$F_{1,3,9}$	0.4438
$F_{2,3,9}$	-0.1410	$F_{3,3,9}$	0.0157	$F_{1,4,9}$	-0.0102	$F_{2,4,9}$	-0.0255
$F_{3,4,9}$	-0.0024	$F_{4,4,9}$	0.0009	$F_{1,5,9}$	0.0202	$F_{2,5,9}$	-0.0316
$F_{3,5,9}$	0.0087	$F_{4,5,9}$	0.0227	$F_{5,5,9}$	0.0351	$F_{1,6,9}$	0.0115
$F_{2,6,9}$	-0.0027	$F_{3,6,9}$	0.0161	$F_{4,6,9}$	0.0081	$F_{5,6,9}$	-0.0547
$F_{6,6,9}$	-0.0161	$F_{1,7,9}$	0.8545	$F_{2,7,9}$	-0.2350	$F_{3,7,9}$	-0.4413
$F_{4,7,9}$	0.0234	$F_{5,7,9}$	-0.0194	$F_{6,7,9}$	-0.0081	$F_{7,7,9}$	-0.8842
$F_{1,8,9}$	-0.2831	$F_{2,8,9}$	-0.0185	$F_{3,8,9}$	0.1333	$F_{4,8,9}$	0.0197

Table S3: The CcCR Force Constants for HOOH

$F_{1,1}$	0.339642	$F_{1,2}$	-0.086915	$F_{1,3}$	-0.227477	$F_{1,4}$	-0.346861	$F_{1,5}$	0.101584
$F_{1,6}$	0.233070	$F_{1,7}$	0.008299	$F_{1,8}$	-0.015015	$F_{1,9}$	-0.007015	$F_{1,10}$	-0.001079
$F_{1,11}$	0.000347	$F_{1,12}$	0.001422	$F_{1,13}$	-0.086915	$F_{2,1}$	0.093010	$F_{2,2}$	0.059326
$F_{2,3}$	0.051486	$F_{2,4}$	-0.054071	$F_{2,5}$	-0.037612	$F_{2,6}$	0.035082	$F_{2,7}$	-0.033382
$F_{2,8}$	-0.018962	$F_{2,9}$	0.000347	$F_{2,10}$	-0.005556	$F_{2,11}$	-0.002752	$F_{2,12}$	-0.227477
$F_{2,13}$	0.059326	$F_{3,1}$	0.156261	$F_{3,2}$	0.233165	$F_{3,3}$	-0.066734	$F_{3,4}$	-0.158785
$F_{3,5}$	-0.004265	$F_{3,6}$	0.004657	$F_{3,7}$	0.001212	$F_{3,8}$	-0.001422	$F_{3,9}$	0.002752
$F_{3,10}$	0.001311	$F_{3,11}$	-0.346861	$F_{3,12}$	0.051486	$F_{3,13}$	0.233165	$F_{4,1}$	0.392542
$F_{4,2}$	-0.087949	$F_{4,3}$	-0.238757	$F_{4,4}$	-0.053980	$F_{4,5}$	0.001381	$F_{4,6}$	0.001327
$F_{4,7}$	0.008299	$F_{4,8}$	0.035082	$F_{4,9}$	0.004265	$F_{4,10}$	0.101584	$F_{4,11}$	-0.054071
$F_{4,12}$	-0.066734	$F_{4,13}$	-0.087949	$F_{5,1}$	0.315182	$F_{5,2}$	0.045021	$F_{5,3}$	0.001381
$F_{5,4}$	-0.227729	$F_{5,5}$	0.026371	$F_{5,6}$	-0.015015	$F_{5,7}$	-0.033382	$F_{5,8}$	-0.004657
$F_{5,9}$	0.233070	$F_{5,10}$	-0.037612	$F_{5,11}$	-0.158785	$F_{5,12}$	-0.238757	$F_{5,13}$	0.045021
$F_{6,1}$	0.177893	$F_{6,2}$	-0.001327	$F_{6,3}$	-0.026371	$F_{6,4}$	-0.020321	$F_{6,5}$	0.007015
$F_{6,6}$	0.018962	$F_{6,7}$	0.001212	$F_{6,8}$	0.008299	$F_{6,9}$	0.035082	$F_{6,10}$	-0.004265
$F_{6,11}$	-0.053980	$F_{6,12}$	0.001381	$F_{6,13}$	-0.001327	$F_{7,1}$	0.392542	$F_{7,2}$	-0.087949
$F_{7,3}$	0.238757	$F_{7,4}$	-0.346861	$F_{7,5}$	0.051486	$F_{7,6}$	-0.233165	$F_{7,7}$	-0.015015
$F_{7,8}$	-0.033382	$F_{7,9}$	0.004657	$F_{7,10}$	0.001381	$F_{7,11}$	-0.227729	$F_{7,12}$	-0.026371
$F_{7,13}$	-0.087949	$F_{8,1}$	0.315182	$F_{8,2}$	-0.045021	$F_{8,3}$	0.101584	$F_{8,4}$	-0.054071
$F_{8,5}$	0.066734	$F_{8,6}$	-0.007015	$F_{8,7}$	-0.018962	$F_{8,8}$	0.001212	$F_{8,9}$	0.001327
$F_{8,10}$	0.026371	$F_{8,11}$	-0.020321	$F_{8,12}$	0.238757	$F_{8,13}$	-0.045021	$F_{9,1}$	0.177893
$F_{9,2}$	-0.233070	$F_{9,3}$	0.037612	$F_{9,4}$	-0.158785	$F_{9,5}$	-0.001079	$F_{9,6}$	0.000347
$F_{9,7}$	-0.001422	$F_{9,8}$	0.008299	$F_{9,9}$	-0.015015	$F_{9,10}$	0.007015	$F_{9,11}$	-0.346861
$F_{9,12}$	0.101584	$F_{9,13}$	-0.233070	$F_{10,1}$	0.339642	$F_{10,2}$	-0.086915	$F_{10,3}$	0.227477
$F_{10,4}$	0.000347	$F_{10,5}$	-0.005556	$F_{10,6}$	0.002752	$F_{10,7}$	0.035082	$F_{10,8}$	-0.033382
$F_{10,9}$	0.018962	$F_{10,10}$	0.051486	$F_{10,11}$	-0.054071	$F_{10,12}$	0.037612	$F_{10,13}$	-0.086915
$F_{11,1}$	0.093010	$F_{11,2}$	-0.059326	$F_{11,3}$	0.001422	$F_{11,4}$	-0.002752	$F_{11,5}$	0.001311
$F_{11,6}$	0.004265	$F_{11,7}$	-0.004657	$F_{11,8}$	0.001212	$F_{11,9}$	-0.233165	$F_{11,10}$	0.066734
$F_{11,11}$	-0.158785	$F_{11,12}$	0.227477	$F_{11,13}$	-0.059326	$F_{12,1}$	0.156261	$F_{1,1,1}$	-0.7734
$F_{1,1,2}$	0.3477	$F_{1,2,2}$	0.0478	$F_{2,2,2}$	-0.0895	$F_{1,1,3}$	0.8344	$F_{1,2,3}$	-0.2802
$F_{2,2,3}$	-0.0291	$F_{1,3,3}$	-0.4432	$F_{2,3,3}$	0.1306	$F_{3,3,3}$	-0.0096	$F_{1,1,4}$	0.7743
$F_{1,2,4}$	-0.3388	$F_{2,2,4}$	-0.0661	$F_{1,3,4}$	-0.8407	$F_{2,3,4}$	0.2550	$F_{3,3,4}$	0.4456
$F_{1,4,4}$	-0.7730	$F_{2,4,4}$	0.3191	$F_{3,4,4}$	0.8656	$F_{4,4,4}$	0.7923	$F_{1,1,5}$	-0.3696
$F_{1,2,5}$	-0.0487	$F_{2,2,5}$	0.1624	$F_{1,3,5}$	0.3028	$F_{2,3,5}$	0.0329	$F_{3,3,5}$	-0.1357
$F_{1,4,5}$	0.3492	$F_{2,4,5}$	0.0265	$F_{3,4,5}$	-0.2810	$F_{4,4,5}$	-0.3690	$F_{1,5,5}$	0.0519
$F_{2,5,5}$	-0.1466	$F_{3,5,5}$	-0.0294	$F_{4,5,5}$	-0.0189	$F_{5,5,5}$	1.0417	$F_{1,1,6}$	-0.8433
$F_{1,2,6}$	0.2596	$F_{2,2,6}$	0.0446	$F_{1,3,6}$	0.4437	$F_{2,3,6}$	-0.1409	$F_{3,3,6}$	0.0158
$F_{1,4,6}$	0.8543	$F_{2,4,6}$	-0.2349	$F_{3,4,6}$	-0.4413	$F_{4,4,6}$	-0.8840	$F_{1,5,6}$	-0.2830
$F_{2,5,6}$	-0.0183	$F_{3,5,6}$	0.1331	$F_{4,5,6}$	0.2617	$F_{5,5,6}$	-0.0153	$F_{1,6,6}$	-0.4570
$F_{2,6,6}$	0.1435	$F_{3,6,6}$	-0.0317	$F_{4,6,6}$	0.4559	$F_{5,6,6}$	-0.2006	$F_{6,6,6}$	0.0573
$F_{1,1,7}$	-0.0030	$F_{1,2,7}$	-0.0086	$F_{2,2,7}$	0.0171	$F_{1,3,7}$	0.0055	$F_{2,3,7}$	0.0266
$F_{3,3,7}$	-0.0020	$F_{1,4,7}$	0.0014	$F_{2,4,7}$	0.0186	$F_{3,4,7}$	-0.0233	$F_{4,4,7}$	-0.0204
$F_{1,5,7}$	0.0197	$F_{2,5,7}$	0.0216	$F_{3,5,7}$	-0.0200	$F_{4,5,7}$	0.0100	$F_{5,5,7}$	-0.0282
$F_{1,6,7}$	-0.0102	$F_{2,6,7}$	-0.0255	$F_{3,6,7}$	-0.0023	$F_{4,6,7}$	0.0233	$F_{5,6,7}$	0.0197
$F_{6,6,7}$	-0.0122	$F_{1,7,7}$	-0.0012	$F_{2,7,7}$	-0.0081	$F_{3,7,7}$	0.0175	$F_{4,7,7}$	0.0204
$F_{5,7,7}$	-0.0418	$F_{6,7,7}$	0.0009	$F_{7,7,7}$	-0.7923	$F_{1,1,8}$	0.0228	$F_{1,2,8}$	0.0003
$F_{2,2,8}$	-0.0714	$F_{1,3,8}$	-0.0199	$F_{2,3,8}$	0.0016	$F_{3,3,8}$	0.0032	$F_{1,4,8}$	-0.0121
$F_{2,4,8}$	0.0387	$F_{3,4,8}$	0.0197	$F_{4,4,8}$	0.0418	$F_{1,5,8}$	-0.0044	$F_{2,5,8}$	-0.0145
$F_{3,5,8}$	-0.0090	$F_{4,5,8}$	-0.0454	$F_{5,5,8}$	-0.8105	$F_{1,6,8}$	0.0201	$F_{2,6,8}$	-0.0316

Table S4: The CcCR Force Constants for HOOH (cont.)

$F_{3,6,8}$	0.0090	$F_{4,6,8}$	-0.0194	$F_{5,6,8}$	0.0090	$F_{6,6,8}$	0.0490	$F_{1,7,8}$	-0.0098
$F_{2,7,8}$	-0.0378	$F_{3,7,8}$	-0.0036	$F_{4,7,8}$	-0.0100	$F_{5,7,8}$	0.0454	$F_{6,7,8}$	0.0229
$F_{7,7,8}$	0.3690	$F_{1,8,8}$	0.0047	$F_{2,8,8}$	0.0846	$F_{3,8,8}$	0.0094	$F_{4,8,8}$	0.0282
$F_{5,8,8}$	0.8105	$F_{6,8,8}$	0.0352	$F_{7,8,8}$	0.0189	$F_{8,8,8}$	-1.0417	$F_{1,1,9}$	0.0084
$F_{1,2,9}$	0.0212	$F_{2,2,9}$	-0.0172	$F_{1,3,9}$	0.0017	$F_{2,3,9}$	0.0097	$F_{3,3,9}$	-0.0064
$F_{1,4,9}$	-0.0140	$F_{2,4,9}$	-0.0171	$F_{3,4,9}$	-0.0065	$F_{4,4,9}$	0.0009	$F_{1,5,9}$	-0.0236
$F_{2,5,9}$	-0.0126	$F_{3,5,9}$	0.0032	$F_{4,5,9}$	0.0229	$F_{5,5,9}$	0.0352	$F_{1,6,9}$	0.0115
$F_{2,6,9}$	-0.0027	$F_{3,6,9}$	0.0161	$F_{4,6,9}$	-0.0080	$F_{5,6,9}$	0.0548	$F_{6,6,9}$	-0.0161
$F_{1,7,9}$	0.0064	$F_{2,7,9}$	-0.0074	$F_{3,7,9}$	0.0066	$F_{4,7,9}$	0.0233	$F_{5,7,9}$	-0.0194
$F_{6,7,9}$	0.0080	$F_{7,7,9}$	-0.8840	$F_{1,8,9}$	0.0016	$F_{2,8,9}$	0.0245	$F_{3,8,9}$	-0.0128
$F_{4,8,9}$	0.0197	$F_{5,8,9}$	0.0090	$F_{6,8,9}$	-0.0548	$F_{7,8,9}$	0.2617	$F_{8,8,9}$	-0.0153
$F_{1,9,9}$	-0.0132	$F_{2,9,9}$	-0.0081	$F_{3,9,9}$	-0.0095	$F_{4,9,9}$	0.0122	$F_{5,9,9}$	-0.0490
$F_{6,9,9}$	-0.0161	$F_{7,9,9}$	-0.4559	$F_{8,9,9}$	0.2006	$F_{9,9,9}$	0.0573	$F_{1,1,10}$	0.0021
$F_{1,2,10}$	-0.0002	$F_{2,2,10}$	0.0012	$F_{1,3,10}$	0.0007	$F_{2,3,10}$	-0.0013	$F_{3,3,10}$	-0.0004
$F_{1,4,10}$	-0.0027	$F_{2,4,10}$	0.0012	$F_{3,4,10}$	-0.0017	$F_{4,4,10}$	0.0012	$F_{1,5,10}$	0.0008
$F_{2,5,10}$	0.0006	$F_{3,5,10}$	-0.0018	$F_{4,5,10}$	0.0098	$F_{5,5,10}$	-0.0047	$F_{1,6,10}$	-0.0007
$F_{2,6,10}$	0.0008	$F_{3,6,10}$	-0.0000	$F_{4,6,10}$	0.0064	$F_{5,6,10}$	0.0016	$F_{6,6,10}$	0.0132
$F_{1,7,10}$	0.0027	$F_{2,7,10}$	-0.0018	$F_{3,7,10}$	0.0004	$F_{4,7,10}$	-0.0014	$F_{5,7,10}$	0.0121
$F_{6,7,10}$	-0.0140	$F_{7,7,10}$	0.7730	$F_{1,8,10}$	-0.0008	$F_{2,8,10}$	-0.0012	$F_{3,8,10}$	0.0038
$F_{4,8,10}$	-0.0197	$F_{5,8,10}$	0.0044	$F_{6,8,10}$	-0.0236	$F_{7,8,10}$	-0.3492	$F_{8,8,10}$	-0.0519
$F_{1,9,10}$	-0.0007	$F_{2,9,10}$	0.0032	$F_{3,9,10}$	-0.0018	$F_{4,9,10}$	-0.0102	$F_{5,9,10}$	0.0201
$F_{6,9,10}$	-0.0115	$F_{7,9,10}$	0.8543	$F_{8,9,10}$	-0.2830	$F_{9,9,10}$	0.4570	$F_{1,10,10}$	-0.0021
$F_{2,10,10}$	0.0008	$F_{3,10,10}$	0.0006	$F_{4,10,10}$	0.0030	$F_{5,10,10}$	-0.0228	$F_{6,10,10}$	0.0084
$F_{7,10,10}$	-0.7743	$F_{8,10,10}$	0.3696	$F_{9,10,10}$	-0.8433	$F_{10,10,10}$	0.7734	$F_{1,1,11}$	-0.0008
$F_{1,2,11}$	0.0006	$F_{2,2,11}$	-0.0015	$F_{1,3,11}$	-0.0027	$F_{2,3,11}$	-0.0053	$F_{3,3,11}$	0.0019
$F_{1,4,11}$	0.0018	$F_{2,4,11}$	0.0009	$F_{3,4,11}$	0.0063	$F_{4,4,11}$	0.0081	$F_{1,5,11}$	0.0012
$F_{2,5,11}$	-0.0013	$F_{3,5,11}$	0.0055	$F_{4,5,11}$	0.0378	$F_{5,5,11}$	-0.0846	$F_{1,6,11}$	0.0032
$F_{2,6,11}$	0.0053	$F_{3,6,11}$	-0.0011	$F_{4,6,11}$	-0.0074	$F_{5,6,11}$	0.0245	$F_{6,6,11}$	0.0081
$F_{1,7,11}$	-0.0012	$F_{2,7,11}$	-0.0009	$F_{3,7,11}$	-0.0030	$F_{4,7,11}$	-0.0186	$F_{5,7,11}$	-0.0387
$F_{6,7,11}$	-0.0171	$F_{7,7,11}$	-0.3191	$F_{1,8,11}$	-0.0006	$F_{2,8,11}$	0.0013	$F_{3,8,11}$	-0.0020
$F_{4,8,11}$	-0.0216	$F_{5,8,11}$	0.0145	$F_{6,8,11}$	-0.0126	$F_{7,8,11}$	-0.0265	$F_{8,8,11}$	0.1466
$F_{1,9,11}$	0.0008	$F_{2,9,11}$	0.0053	$F_{3,9,11}$	-0.0001	$F_{4,9,11}$	-0.0255	$F_{5,9,11}$	-0.0316
$F_{6,9,11}$	0.0027	$F_{7,9,11}$	-0.2349	$F_{8,9,11}$	-0.0183	$F_{9,9,11}$	-0.1435	$F_{1,10,11}$	0.0002
$F_{2,10,11}$	-0.0006	$F_{3,10,11}$	-0.0006	$F_{4,10,11}$	0.0086	$F_{5,10,11}$	-0.0003	$F_{6,10,11}$	0.0212
$F_{7,10,11}$	0.3388	$F_{8,10,11}$	0.0487	$F_{9,10,11}$	0.2596	$F_{10,10,11}$	-0.3477	$F_{1,11,11}$	-0.0012
$F_{2,11,11}$	0.0015	$F_{3,11,11}$	0.0018	$F_{4,11,11}$	-0.0171	$F_{5,11,11}$	0.0714	$F_{6,11,11}$	-0.0172
$F_{7,11,11}$	0.0661	$F_{8,11,11}$	-0.1624	$F_{9,11,11}$	0.0446	$F_{10,11,11}$	-0.0478	$F_{11,11,11}$	0.0895
$F_{1,1,12}$	0.0006	$F_{1,2,12}$	-0.0006	$F_{2,2,12}$	0.0018	$F_{1,3,12}$	-0.0022	$F_{2,3,12}$	0.0007
$F_{3,3,12}$	0.0002	$F_{1,4,12}$	0.0004	$F_{2,4,12}$	-0.0030	$F_{3,4,12}$	0.0022	$F_{4,4,12}$	0.0175
$F_{1,5,12}$	0.0038	$F_{2,5,12}$	-0.0020	$F_{3,5,12}$	-0.0006	$F_{4,5,12}$	-0.0036	$F_{5,5,12}$	0.0094
$F_{1,6,12}$	0.0018	$F_{2,6,12}$	0.0001	$F_{3,6,12}$	-0.0002	$F_{4,6,12}$	-0.0066	$F_{5,6,12}$	0.0128
$F_{6,6,12}$	-0.0095	$F_{1,7,12}$	-0.0017	$F_{2,7,12}$	0.0063	$F_{3,7,12}$	-0.0022	$F_{4,7,12}$	-0.0233
$F_{5,7,12}$	0.0197	$F_{6,7,12}$	0.0065	$F_{7,7,12}$	0.8656	$F_{1,8,12}$	-0.0018	$F_{2,8,12}$	0.0055
$F_{3,8,12}$	0.0006	$F_{4,8,12}$	-0.0200	$F_{5,8,12}$	-0.0090	$F_{6,8,12}$	-0.0032	$F_{7,8,12}$	-0.2810
$F_{8,8,12}$	-0.0294	$F_{1,9,12}$	0.0000	$F_{2,9,12}$	0.0011	$F_{3,9,12}$	-0.0002	$F_{4,9,12}$	0.0023
$F_{5,9,12}$	-0.0090	$F_{6,9,12}$	0.0161	$F_{7,9,12}$	0.4413	$F_{8,9,12}$	-0.1331	$F_{9,9,12}$	-0.0317
$F_{1,10,12}$	0.0007	$F_{2,10,12}$	-0.0027	$F_{3,10,12}$	0.0022	$F_{4,10,12}$	0.0055	$F_{5,10,12}$	-0.0199
$F_{6,10,12}$	-0.0017	$F_{7,10,12}$	-0.8407	$F_{8,10,12}$	0.3028	$F_{9,10,12}$	-0.4437	$F_{10,10,12}$	0.8344
$F_{1,11,12}$	-0.0013	$F_{2,11,12}$	-0.0053	$F_{3,11,12}$	-0.0007	$F_{4,11,12}$	0.0266	$F_{5,11,12}$	0.0016

Table S5: The CcCR Force Constants for HOOH (cont.)

$F_{6,11,12}$	-0.0097	$F_{7,11,12}$	0.2550	$F_{8,11,12}$	0.0329	$F_{9,11,12}$	0.1409	$F_{10,11,12}$	-0.2802
$F_{11,11,12}$	-0.0291	$F_{1,12,12}$	0.0004	$F_{2,12,12}$	-0.0019	$F_{3,12,12}$	0.0002	$F_{4,12,12}$	0.0020
$F_{5,12,12}$	-0.0032	$F_{6,12,12}$	-0.0064	$F_{7,12,12}$	-0.4456	$F_{8,12,12}$	0.1357	$F_{9,12,12}$	0.0158
$F_{10,12,12}$	0.4432	$F_{11,12,12}$	-0.1306	$F_{12,12,12}$	-0.0096	$F_{1,1,1,1}$	0.80	$F_{1,1,1,2}$	-0.97
$F_{1,1,2,2}$	-0.34	$F_{1,2,2,2}$	0.59	$F_{2,2,2,2}$	-0.11	$F_{1,1,1,3}$	-2.25	$F_{1,1,2,3}$	1.04
$F_{1,2,2,3}$	0.25	$F_{2,2,2,3}$	-0.40	$F_{1,1,3,3}$	1.60	$F_{1,2,3,3}$	-0.56	$F_{2,2,3,3}$	-0.13
$F_{1,3,3,3}$	0.06	$F_{2,3,3,3}$	-0.01	$F_{3,3,3,3}$	-0.95	$F_{1,1,1,4}$	-0.80	$F_{1,1,2,4}$	0.98
$F_{1,2,2,4}$	0.35	$F_{2,2,2,4}$	-0.57	$F_{1,1,3,4}$	2.27	$F_{1,2,3,4}$	-1.02	$F_{2,2,3,4}$	-0.27
$F_{1,3,3,4}$	-1.62	$F_{2,3,3,4}$	0.55	$F_{3,3,3,4}$	-0.06	$F_{1,1,4,4}$	0.78	$F_{1,2,4,4}$	-1.01
$F_{2,2,4,4}$	-0.42	$F_{1,3,4,4}$	-2.29	$F_{2,3,4,4}$	1.01	$F_{3,3,4,4}$	1.63	$F_{1,4,4,4}$	-0.74
$F_{2,4,4,4}$	1.09	$F_{3,4,4,4}$	2.33	$F_{4,4,4,4}$	0.66	$F_{1,1,1,5}$	0.95	$F_{1,1,2,5}$	0.32
$F_{1,2,2,5}$	-0.67	$F_{2,2,2,5}$	0.18	$F_{1,1,3,5}$	-1.06	$F_{1,2,3,5}$	-0.25	$F_{2,2,3,5}$	0.46
$F_{1,3,3,5}$	0.55	$F_{2,3,3,5}$	0.13	$F_{3,3,3,5}$	0.04	$F_{1,1,4,5}$	-0.95	$F_{1,2,4,5}$	-0.33
$F_{2,2,4,5}$	0.62	$F_{1,3,4,5}$	1.04	$F_{2,3,4,5}$	0.24	$F_{3,3,4,5}$	-0.55	$F_{1,4,4,5}$	0.97
$F_{2,4,4,5}$	0.35	$F_{3,4,4,5}$	-1.01	$F_{4,4,4,5}$	-1.03	$F_{1,1,5,5}$	-0.36	$F_{1,2,5,5}$	0.71
$F_{2,2,5,5}$	-0.18	$F_{1,3,5,5}$	0.28	$F_{2,3,5,5}$	-0.49	$F_{3,3,5,5}$	-0.16	$F_{1,4,5,5}$	0.34
$F_{2,4,5,5}$	-0.69	$F_{3,4,5,5}$	-0.26	$F_{4,4,5,5}$	-0.64	$F_{1,5,5,5}$	-0.81	$F_{2,5,5,5}$	0.23
$F_{3,5,5,5}$	0.58	$F_{4,5,5,5}$	0.93	$F_{5,5,5,5}$	2.02	$F_{1,1,1,6}$	2.27	$F_{1,1,2,6}$	-1.02
$F_{1,2,2,6}$	-0.28	$F_{2,2,2,6}$	0.40	$F_{1,1,3,6}$	-1.61	$F_{1,2,3,6}$	0.56	$F_{2,2,3,6}$	0.14
$F_{1,3,3,6}$	-0.07	$F_{2,3,3,6}$	-0.02	$F_{3,3,3,6}$	0.96	$F_{1,1,4,6}$	-2.28	$F_{1,2,4,6}$	1.00
$F_{2,2,4,6}$	0.30	$F_{1,3,4,6}$	1.63	$F_{2,3,4,6}$	-0.56	$F_{3,3,4,6}$	0.09	$F_{1,4,4,6}$	2.30
$F_{2,4,4,6}$	-1.00	$F_{3,4,4,6}$	-1.65	$F_{4,4,4,6}$	-2.33	$F_{1,1,5,6}$	1.03	$F_{1,2,5,6}$	0.25
$F_{2,2,5,6}$	-0.43	$F_{1,3,5,6}$	-0.55	$F_{2,3,5,6}$	-0.15	$F_{3,3,5,6}$	-0.02	$F_{1,4,5,6}$	-1.01
$F_{2,4,5,6}$	-0.24	$F_{3,4,5,6}$	0.56	$F_{4,4,5,6}$	0.99	$F_{1,5,5,6}$	-0.27	$F_{2,5,5,6}$	0.49
$F_{3,5,5,6}$	0.15	$F_{4,5,5,6}$	0.25	$F_{5,5,5,6}$	-0.65	$F_{1,1,6,6}$	1.64	$F_{1,2,6,6}$	-0.57
$F_{2,2,6,6}$	-0.18	$F_{1,3,6,6}$	0.07	$F_{2,3,6,6}$	0.05	$F_{3,3,6,6}$	-0.97	$F_{1,4,6,6}$	-1.66
$F_{2,4,6,6}$	0.58	$F_{3,4,6,6}$	-0.12	$F_{4,4,6,6}$	1.68	$F_{1,5,6,6}$	0.55	$F_{2,5,6,6}$	0.17
$F_{3,5,6,6}$	-0.03	$F_{4,5,6,6}$	-0.57	$F_{5,5,6,6}$	-0.51	$F_{1,6,6,6}$	-0.09	$F_{2,6,6,6}$	-0.13
$F_{3,6,6,6}$	0.99	$F_{4,6,6,6}$	0.16	$F_{5,6,6,6}$	0.14	$F_{6,6,6,6}$	-0.99	$F_{1,1,1,7}$	-0.00
$F_{1,1,2,7}$	-0.02	$F_{1,2,2,7}$	-0.02	$F_{2,2,2,7}$	-0.03	$F_{1,1,3,7}$	-0.01	$F_{1,2,3,7}$	-0.02
$F_{2,2,3,7}$	0.03	$F_{1,3,3,7}$	0.01	$F_{2,3,3,7}$	0.01	$F_{3,3,3,7}$	0.00	$F_{1,1,4,7}$	0.01
$F_{1,2,4,7}$	0.02	$F_{2,2,4,7}$	0.06	$F_{1,3,4,7}$	0.03	$F_{2,3,4,7}$	0.01	$F_{3,3,4,7}$	-0.02
$F_{1,4,4,7}$	-0.03	$F_{2,4,4,7}$	-0.08	$F_{3,4,4,7}$	-0.04	$F_{4,4,4,7}$	0.09	$F_{1,1,5,7}$	0.01
$F_{1,2,5,7}$	0.01	$F_{2,2,5,7}$	0.05	$F_{1,3,5,7}$	0.01	$F_{2,3,5,7}$	0.00	$F_{3,3,5,7}$	-0.01
$F_{1,4,5,7}$	-0.02	$F_{2,4,5,7}$	-0.02	$F_{3,4,5,7}$	-0.02	$F_{4,4,5,7}$	0.05	$F_{1,5,5,7}$	0.02
$F_{2,5,5,7}$	-0.03	$F_{3,5,5,7}$	-0.01	$F_{4,5,5,7}$	0.27	$F_{5,5,5,7}$	-0.18	$F_{1,1,6,7}$	0.01
$F_{1,2,6,7}$	0.02	$F_{2,2,6,7}$	-0.02	$F_{1,3,6,7}$	-0.01	$F_{2,3,6,7}$	-0.00	$F_{3,3,6,7}$	-0.02
$F_{1,4,6,7}$	-0.02	$F_{2,4,6,7}$	-0.01	$F_{3,4,6,7}$	0.02	$F_{4,4,6,7}$	0.03	$F_{1,5,6,7}$	-0.02
$F_{2,5,6,7}$	-0.00	$F_{3,5,6,7}$	-0.00	$F_{4,5,6,7}$	0.02	$F_{5,5,6,7}$	0.02	$F_{1,6,6,7}$	0.02
$F_{2,6,6,7}$	-0.01	$F_{3,6,6,7}$	0.04	$F_{4,6,6,7}$	-0.02	$F_{5,6,6,7}$	0.00	$F_{6,6,6,7}$	-0.06
$F_{1,1,7,7}$	-0.01	$F_{1,2,7,7}$	-0.00	$F_{2,2,7,7}$	-0.04	$F_{1,3,7,7}$	-0.01	$F_{2,3,7,7}$	0.01
$F_{3,3,7,7}$	0.01	$F_{1,4,7,7}$	0.02	$F_{2,4,7,7}$	0.06	$F_{3,4,7,7}$	0.01	$F_{4,4,7,7}$	-0.08
$F_{1,5,7,7}$	0.00	$F_{2,5,7,7}$	0.01	$F_{3,5,7,7}$	0.01	$F_{4,5,7,7}$	-0.03	$F_{5,5,7,7}$	-0.33
$F_{1,6,7,7}$	0.01	$F_{2,6,7,7}$	-0.01	$F_{3,6,7,7}$	0.00	$F_{4,6,7,7}$	-0.02	$F_{5,6,7,7}$	0.01
$F_{6,6,7,7}$	0.01	$F_{1,7,7,7}$	-0.01	$F_{2,7,7,7}$	-0.06	$F_{3,7,7,7}$	-0.00	$F_{4,7,7,7}$	0.09
$F_{5,7,7,7}$	-0.00	$F_{6,7,7,7}$	0.00	$F_{7,7,7,7}$	0.66	$F_{1,1,1,8}$	0.02	$F_{1,1,2,8}$	0.02
$F_{1,2,2,8}$	0.07	$F_{2,2,2,8}$	-0.07	$F_{1,1,3,8}$	0.02	$F_{1,2,3,8}$	-0.00	$F_{2,2,3,8}$	-0.05
$F_{1,3,3,8}$	0.01	$F_{2,3,3,8}$	0.01	$F_{3,3,3,8}$	-0.03	$F_{1,1,4,8}$	-0.03	$F_{1,2,4,8}$	-0.02
$F_{2,2,4,8}$	-0.04	$F_{1,3,4,8}$	-0.02	$F_{2,3,4,8}$	0.03	$F_{3,3,4,8}$	-0.01	$F_{1,4,4,8}$	0.03

Table S6: The CcCR Force Constants for HOOH (cont.)

$F_{2,4,4,8}$	0.05	$F_{3,4,4,8}$	0.00	$F_{4,4,4,8}$	-0.00	$F_{1,1,5,8}$	0.04	$F_{1,2,5,8}$	-0.05
$F_{2,2,5,8}$	0.00	$F_{1,3,5,8}$	-0.03	$F_{2,3,5,8}$	0.03	$F_{3,3,5,8}$	0.02	$F_{1,4,5,8}$	-0.01
$F_{2,4,5,8}$	0.06	$F_{3,4,5,8}$	0.01	$F_{4,4,5,8}$	0.27	$F_{1,5,5,8}$	0.09	$F_{2,5,5,8}$	-0.06
$F_{3,5,5,8}$	-0.09	$F_{4,5,5,8}$	-0.27	$F_{5,5,5,8}$	-2.11	$F_{1,1,6,8}$	-0.01	$F_{1,2,6,8}$	0.03
$F_{2,2,6,8}$	0.03	$F_{1,3,6,8}$	-0.01	$F_{2,3,6,8}$	0.01	$F_{3,3,6,8}$	0.03	$F_{1,4,6,8}$	0.01
$F_{2,4,6,8}$	-0.06	$F_{3,4,6,8}$	0.01	$F_{4,4,6,8}$	0.02	$F_{1,5,6,8}$	0.02	$F_{2,5,6,8}$	-0.06
$F_{3,5,6,8}$	-0.00	$F_{4,5,6,8}$	0.00	$F_{5,5,6,8}$	0.12	$F_{1,6,6,8}$	0.03	$F_{2,6,6,8}$	0.01
$F_{3,6,6,8}$	-0.02	$F_{4,6,6,8}$	-0.01	$F_{5,6,6,8}$	0.30	$F_{6,6,6,8}$	0.02	$F_{1,1,7,8}$	0.01
$F_{1,2,7,8}$	0.01	$F_{2,2,7,8}$	-0.03	$F_{1,3,7,8}$	0.00	$F_{2,3,7,8}$	-0.03	$F_{3,3,7,8}$	0.00
$F_{1,4,7,8}$	-0.01	$F_{2,4,7,8}$	-0.03	$F_{3,4,7,8}$	0.01	$F_{4,4,7,8}$	-0.03	$F_{1,5,7,8}$	-0.03
$F_{2,5,7,8}$	-0.01	$F_{3,5,7,8}$	0.01	$F_{4,5,7,8}$	-0.23	$F_{5,5,7,8}$	0.22	$F_{1,6,7,8}$	0.00
$F_{2,6,7,8}$	0.02	$F_{3,6,7,8}$	0.00	$F_{4,6,7,8}$	-0.02	$F_{5,6,7,8}$	0.01	$F_{6,6,7,8}$	-0.01
$F_{1,7,7,8}$	0.00	$F_{2,7,7,8}$	0.02	$F_{3,7,7,8}$	-0.01	$F_{4,7,7,8}$	0.05	$F_{5,7,7,8}$	0.27
$F_{6,7,7,8}$	-0.01	$F_{7,7,7,8}$	-1.03	$F_{1,1,8,8}$	-0.05	$F_{1,2,8,8}$	-0.02	$F_{2,2,8,8}$	0.06
$F_{1,3,8,8}$	0.04	$F_{2,3,8,8}$	0.02	$F_{3,3,8,8}$	-0.02	$F_{1,4,8,8}$	0.03	$F_{2,4,8,8}$	-0.02
$F_{3,4,8,8}$	-0.05	$F_{4,4,8,8}$	-0.33	$F_{1,5,8,8}$	-0.04	$F_{2,5,8,8}$	0.06	$F_{3,5,8,8}$	0.06
$F_{4,5,8,8}$	0.22	$F_{5,5,8,8}$	2.11	$F_{1,6,8,8}$	-0.05	$F_{2,6,8,8}$	0.03	$F_{3,6,8,8}$	-0.01
$F_{4,6,8,8}$	0.06	$F_{5,6,8,8}$	-0.03	$F_{6,6,8,8}$	-0.32	$F_{1,7,8,8}$	0.02	$F_{2,7,8,8}$	0.03
$F_{3,7,8,8}$	0.03	$F_{4,7,8,8}$	0.27	$F_{5,7,8,8}$	-0.27	$F_{6,7,8,8}$	-0.04	$F_{7,7,8,8}$	-0.64
$F_{1,8,8,8}$	0.06	$F_{2,8,8,8}$	-0.13	$F_{3,8,8,8}$	-0.08	$F_{4,8,8,8}$	-0.18	$F_{5,8,8,8}$	-2.11
$F_{6,8,8,8}$	0.02	$F_{7,8,8,8}$	0.93	$F_{8,8,8,8}$	2.02	$F_{1,1,1,9}$	-0.02	$F_{1,1,2,9}$	-0.01
$F_{1,2,2,9}$	0.03	$F_{2,2,2,9}$	0.00	$F_{1,1,3,9}$	0.01	$F_{1,2,3,9}$	-0.00	$F_{2,2,3,9}$	-0.00
$F_{1,3,3,9}$	0.01	$F_{2,3,3,9}$	0.03	$F_{3,3,3,9}$	-0.00	$F_{1,1,4,9}$	0.01	$F_{1,2,4,9}$	0.01
$F_{2,2,4,9}$	-0.02	$F_{1,3,4,9}$	-0.01	$F_{2,3,4,9}$	0.01	$F_{3,3,4,9}$	-0.03	$F_{1,4,4,9}$	-0.01
$F_{2,4,4,9}$	-0.01	$F_{3,4,4,9}$	0.01	$F_{4,4,4,9}$	-0.00	$F_{1,1,5,9}$	0.02	$F_{1,2,5,9}$	0.00
$F_{2,2,5,9}$	-0.03	$F_{1,3,5,9}$	0.00	$F_{2,3,5,9}$	0.01	$F_{3,3,5,9}$	-0.02	$F_{1,4,5,9}$	-0.03
$F_{2,4,5,9}$	-0.01	$F_{3,4,5,9}$	-0.01	$F_{4,4,5,9}$	0.01	$F_{1,5,5,9}$	-0.03	$F_{2,5,5,9}$	0.01
$F_{3,5,5,9}$	0.01	$F_{4,5,5,9}$	0.04	$F_{5,5,5,9}$	-0.02	$F_{1,1,6,9}$	-0.02	$F_{1,2,6,9}$	0.01
$F_{2,2,6,9}$	0.04	$F_{1,3,6,9}$	-0.01	$F_{2,3,6,9}$	-0.03	$F_{3,3,6,9}$	0.01	$F_{1,4,6,9}$	0.02
$F_{2,4,6,9}$	-0.03	$F_{3,4,6,9}$	0.03	$F_{4,4,6,9}$	-0.02	$F_{1,5,6,9}$	0.01	$F_{2,5,6,9}$	-0.02
$F_{3,5,6,9}$	0.05	$F_{4,5,6,9}$	0.02	$F_{5,5,6,9}$	0.32	$F_{1,6,6,9}$	0.02	$F_{2,6,6,9}$	0.07
$F_{3,6,6,9}$	-0.02	$F_{4,6,6,9}$	-0.04	$F_{5,6,6,9}$	-0.09	$F_{6,6,6,9}$	0.01	$F_{1,1,7,9}$	0.01
$F_{1,2,7,9}$	0.00	$F_{2,2,7,9}$	-0.01	$F_{1,3,7,9}$	0.00	$F_{2,3,7,9}$	-0.01	$F_{3,3,7,9}$	0.02
$F_{1,4,7,9}$	-0.01	$F_{2,4,7,9}$	-0.01	$F_{3,4,7,9}$	0.01	$F_{4,4,7,9}$	0.02	$F_{1,5,7,9}$	0.01
$F_{2,5,7,9}$	0.00	$F_{3,5,7,9}$	0.01	$F_{4,5,7,9}$	0.02	$F_{5,5,7,9}$	-0.06	$F_{1,6,7,9}$	0.00
$F_{2,6,7,9}$	0.02	$F_{3,6,7,9}$	-0.02	$F_{4,6,7,9}$	-0.00	$F_{5,6,7,9}$	-0.01	$F_{6,6,7,9}$	0.01
$F_{1,7,7,9}$	-0.00	$F_{2,7,7,9}$	0.01	$F_{3,7,7,9}$	-0.01	$F_{4,7,7,9}$	-0.03	$F_{5,7,7,9}$	-0.02
$F_{6,7,7,9}$	-0.02	$F_{7,7,7,9}$	2.33	$F_{1,1,8,9}$	-0.00	$F_{1,2,8,9}$	-0.03	$F_{2,2,8,9}$	0.02
$F_{1,3,8,9}$	-0.00	$F_{2,3,8,9}$	-0.01	$F_{3,3,8,9}$	-0.00	$F_{1,4,8,9}$	0.01	$F_{2,4,8,9}$	0.02
$F_{3,4,8,9}$	0.01	$F_{4,4,8,9}$	-0.01	$F_{1,5,8,9}$	0.02	$F_{2,5,8,9}$	0.02	$F_{3,5,8,9}$	-0.02
$F_{4,5,8,9}$	-0.01	$F_{5,5,8,9}$	0.03	$F_{1,6,8,9}$	-0.02	$F_{2,6,8,9}$	-0.02	$F_{3,6,8,9}$	-0.02
$F_{4,6,8,9}$	-0.01	$F_{5,6,8,9}$	-0.28	$F_{6,6,8,9}$	-0.03	$F_{1,7,8,9}$	-0.01	$F_{2,7,8,9}$	0.01
$F_{3,7,8,9}$	-0.01	$F_{4,7,8,9}$	-0.02	$F_{5,7,8,9}$	-0.00	$F_{6,7,8,9}$	0.02	$F_{7,7,8,9}$	-0.99
$F_{1,8,8,9}$	0.00	$F_{2,8,8,9}$	-0.04	$F_{3,8,8,9}$	0.04	$F_{4,8,8,9}$	-0.02	$F_{5,8,8,9}$	-0.12
$F_{6,8,8,9}$	0.32	$F_{7,8,8,9}$	-0.25	$F_{8,8,8,9}$	0.65	$F_{1,1,9,9}$	0.01	$F_{1,2,9,9}$	-0.01
$F_{2,2,9,9}$	-0.03	$F_{1,3,9,9}$	0.00	$F_{2,3,9,9}$	0.00	$F_{3,3,9,9}$	-0.00	$F_{1,4,9,9}$	-0.01
$F_{2,4,9,9}$	0.02	$F_{3,4,9,9}$	-0.00	$F_{4,4,9,9}$	0.01	$F_{1,5,9,9}$	-0.01	$F_{2,5,9,9}$	0.01
$F_{3,5,9,9}$	-0.02	$F_{4,5,9,9}$	-0.01	$F_{5,5,9,9}$	-0.32	$F_{1,6,9,9}$	-0.01	$F_{2,6,9,9}$	-0.04
$F_{3,6,9,9}$	0.02	$F_{4,6,9,9}$	-0.01	$F_{5,6,9,9}$	0.03	$F_{6,6,9,9}$	-0.00	$F_{1,7,9,9}$	-0.01

Table S7: The CcCR Force Constants for HOOH (cont.)

$F_{2,7,9,9}$	-0.00	$F_{3,7,9,9}$	0.00	$F_{4,7,9,9}$	-0.02	$F_{5,7,9,9}$	-0.01	$F_{6,7,9,9}$	0.04
$F_{7,7,9,9}$	1.68	$F_{1,8,9,9}$	0.02	$F_{2,8,9,9}$	0.04	$F_{3,8,9,9}$	0.02	$F_{4,8,9,9}$	0.00
$F_{5,8,9,9}$	0.30	$F_{6,8,9,9}$	0.09	$F_{7,8,9,9}$	-0.57	$F_{8,8,9,9}$	-0.51	$F_{1,9,9,9}$	0.01
$F_{2,9,9,9}$	0.03	$F_{3,9,9,9}$	-0.01	$F_{4,9,9,9}$	0.06	$F_{5,9,9,9}$	-0.02	$F_{6,9,9,9}$	0.01
$F_{7,9,9,9}$	-0.16	$F_{8,9,9,9}$	-0.14	$F_{9,9,9,9}$	-0.99	$F_{1,1,1,10}$	-0.00	$F_{1,1,2,10}$	0.00
$F_{1,2,2,10}$	-0.00	$F_{2,2,2,10}$	0.00	$F_{1,1,3,10}$	0.00	$F_{1,2,3,10}$	0.00	$F_{2,2,3,10}$	-0.00
$F_{1,3,3,10}$	0.00	$F_{2,3,3,10}$	-0.00	$F_{3,3,3,10}$	0.00	$F_{1,1,4,10}$	0.00	$F_{1,2,4,10}$	-0.00
$F_{2,2,4,10}$	0.00	$F_{1,3,4,10}$	-0.00	$F_{2,3,4,10}$	-0.00	$F_{3,3,4,10}$	-0.00	$F_{1,4,4,10}$	-0.00
$F_{2,4,4,10}$	-0.00	$F_{3,4,4,10}$	-0.00	$F_{4,4,4,10}$	-0.01	$F_{1,1,5,10}$	0.00	$F_{1,2,5,10}$	0.00
$F_{2,2,5,10}$	0.00	$F_{1,3,5,10}$	0.00	$F_{2,3,5,10}$	0.00	$F_{3,3,5,10}$	0.00	$F_{1,4,5,10}$	-0.00
$F_{2,4,5,10}$	-0.00	$F_{3,4,5,10}$	-0.01	$F_{4,4,5,10}$	0.00	$F_{1,5,5,10}$	-0.00	$F_{2,5,5,10}$	0.00
$F_{3,5,5,10}$	-0.01	$F_{4,5,5,10}$	0.02	$F_{5,5,5,10}$	0.06	$F_{1,1,6,10}$	-0.00	$F_{1,2,6,10}$	-0.00
$F_{2,2,6,10}$	-0.00	$F_{1,3,6,10}$	-0.00	$F_{2,3,6,10}$	0.00	$F_{3,3,6,10}$	-0.00	$F_{1,4,6,10}$	0.00
$F_{2,4,6,10}$	0.00	$F_{3,4,6,10}$	0.00	$F_{4,4,6,10}$	0.00	$F_{1,5,6,10}$	-0.00	$F_{2,5,6,10}$	-0.00
$F_{3,5,6,10}$	-0.00	$F_{4,5,6,10}$	0.01	$F_{5,5,6,10}$	-0.00	$F_{1,6,6,10}$	0.00	$F_{2,6,6,10}$	-0.00
$F_{3,6,6,10}$	0.00	$F_{4,6,6,10}$	-0.01	$F_{5,6,6,10}$	0.02	$F_{6,6,6,10}$	-0.01	$F_{1,1,7,10}$	0.00
$F_{1,2,7,10}$	-0.00	$F_{2,2,7,10}$	-0.00	$F_{1,3,7,10}$	0.00	$F_{2,3,7,10}$	-0.00	$F_{3,3,7,10}$	0.00
$F_{1,4,7,10}$	0.00	$F_{2,4,7,10}$	0.00	$F_{3,4,7,10}$	-0.00	$F_{4,4,7,10}$	0.02	$F_{1,5,7,10}$	0.01
$F_{2,5,7,10}$	0.00	$F_{3,5,7,10}$	0.00	$F_{4,5,7,10}$	-0.01	$F_{5,5,7,10}$	0.03	$F_{1,6,7,10}$	-0.00
$F_{2,6,7,10}$	-0.00	$F_{3,6,7,10}$	-0.00	$F_{4,6,7,10}$	0.01	$F_{5,6,7,10}$	-0.01	$F_{6,6,7,10}$	-0.01
$F_{1,7,7,10}$	-0.00	$F_{2,7,7,10}$	0.01	$F_{3,7,7,10}$	-0.00	$F_{4,7,7,10}$	-0.03	$F_{5,7,7,10}$	0.03
$F_{6,7,7,10}$	0.01	$F_{7,7,7,10}$	-0.74	$F_{1,1,8,10}$	-0.00	$F_{1,2,8,10}$	-0.00	$F_{2,2,8,10}$	-0.00
$F_{1,3,8,10}$	-0.00	$F_{2,3,8,10}$	0.00	$F_{3,3,8,10}$	0.00	$F_{1,4,8,10}$	0.01	$F_{2,4,8,10}$	-0.00
$F_{3,4,8,10}$	0.01	$F_{4,4,8,10}$	0.00	$F_{1,5,8,10}$	0.00	$F_{2,5,8,10}$	-0.00	$F_{3,5,8,10}$	0.01
$F_{4,5,8,10}$	-0.03	$F_{5,5,8,10}$	-0.04	$F_{1,6,8,10}$	0.00	$F_{2,6,8,10}$	0.00	$F_{3,6,8,10}$	0.00
$F_{4,6,8,10}$	-0.01	$F_{5,6,8,10}$	-0.02	$F_{6,6,8,10}$	-0.01	$F_{1,7,8,10}$	-0.00	$F_{2,7,8,10}$	0.00
$F_{3,7,8,10}$	0.00	$F_{4,7,8,10}$	-0.02	$F_{5,7,8,10}$	-0.01	$F_{6,7,8,10}$	0.03	$F_{7,7,8,10}$	0.97
$F_{1,8,8,10}$	-0.00	$F_{2,8,8,10}$	0.01	$F_{3,8,8,10}$	-0.02	$F_{4,8,8,10}$	0.02	$F_{5,8,8,10}$	0.09
$F_{6,8,8,10}$	0.03	$F_{7,8,8,10}$	0.34	$F_{8,8,8,10}$	-0.81	$F_{1,1,9,10}$	-0.00	$F_{1,2,9,10}$	-0.00
$F_{2,2,9,10}$	0.00	$F_{1,3,9,10}$	0.00	$F_{2,3,9,10}$	0.00	$F_{3,3,9,10}$	-0.00	$F_{1,4,9,10}$	0.00
$F_{2,4,9,10}$	0.00	$F_{3,4,9,10}$	-0.00	$F_{4,4,9,10}$	-0.01	$F_{1,5,9,10}$	-0.00	$F_{2,5,9,10}$	-0.00
$F_{3,5,9,10}$	-0.00	$F_{4,5,9,10}$	-0.00	$F_{5,5,9,10}$	0.05	$F_{1,6,9,10}$	-0.00	$F_{2,6,9,10}$	-0.00
$F_{3,6,9,10}$	0.00	$F_{4,6,9,10}$	0.00	$F_{5,6,9,10}$	-0.02	$F_{6,6,9,10}$	0.01	$F_{1,7,9,10}$	-0.00
$F_{2,7,9,10}$	-0.00	$F_{3,7,9,10}$	0.00	$F_{4,7,9,10}$	0.02	$F_{5,7,9,10}$	-0.01	$F_{6,7,9,10}$	0.02
$F_{7,7,9,10}$	-2.30	$F_{1,8,9,10}$	0.00	$F_{2,8,9,10}$	-0.00	$F_{3,8,9,10}$	0.00	$F_{4,8,9,10}$	0.02
$F_{5,8,9,10}$	-0.02	$F_{6,8,9,10}$	0.01	$F_{7,8,9,10}$	1.01	$F_{8,8,9,10}$	0.27	$F_{1,9,9,10}$	0.00
$F_{2,9,9,10}$	-0.01	$F_{3,9,9,10}$	0.00	$F_{4,9,9,10}$	0.02	$F_{5,9,9,10}$	0.03	$F_{6,9,9,10}$	-0.02
$F_{7,9,9,10}$	-1.66	$F_{8,9,9,10}$	0.55	$F_{9,9,9,10}$	0.09	$F_{1,1,10,10}$	-0.00	$F_{1,2,10,10}$	-0.00
$F_{2,2,10,10}$	0.00	$F_{1,3,10,10}$	-0.00	$F_{2,3,10,10}$	-0.00	$F_{3,3,10,10}$	-0.00	$F_{1,4,10,10}$	0.00
$F_{2,4,10,10}$	0.00	$F_{3,4,10,10}$	0.00	$F_{4,4,10,10}$	-0.01	$F_{1,5,10,10}$	-0.00	$F_{2,5,10,10}$	-0.00
$F_{3,5,10,10}$	0.00	$F_{4,5,10,10}$	0.01	$F_{5,5,10,10}$	-0.05	$F_{1,6,10,10}$	0.00	$F_{2,6,10,10}$	0.00
$F_{3,6,10,10}$	0.00	$F_{4,6,10,10}$	-0.01	$F_{5,6,10,10}$	0.00	$F_{6,6,10,10}$	0.01	$F_{1,7,10,10}$	0.00
$F_{2,7,10,10}$	-0.01	$F_{3,7,10,10}$	0.00	$F_{4,7,10,10}$	0.01	$F_{5,7,10,10}$	-0.03	$F_{6,7,10,10}$	-0.01
$F_{7,7,10,10}$	0.78	$F_{1,8,10,10}$	0.00	$F_{2,8,10,10}$	-0.00	$F_{3,8,10,10}$	-0.00	$F_{4,8,10,10}$	0.01
$F_{5,8,10,10}$	0.04	$F_{6,8,10,10}$	-0.02	$F_{7,8,10,10}$	-0.95	$F_{8,8,10,10}$	-0.36	$F_{1,9,10,10}$	0.00
$F_{2,9,10,10}$	0.00	$F_{3,9,10,10}$	-0.00	$F_{4,9,10,10}$	-0.01	$F_{5,9,10,10}$	0.01	$F_{6,9,10,10}$	-0.02
$F_{7,9,10,10}$	2.28	$F_{8,9,10,10}$	-1.03	$F_{9,9,10,10}$	1.64	$F_{1,10,10,10}$	-0.00	$F_{2,10,10,10}$	0.00
$F_{3,10,10,10}$	-0.00	$F_{4,10,10,10}$	-0.00	$F_{5,10,10,10}$	0.02	$F_{6,10,10,10}$	0.02	$F_{7,10,10,10}$	-0.80
$F_{8,10,10,10}$	0.95	$F_{9,10,10,10}$	-2.27	$F_{10,10,10,10}$	0.80	$F_{1,1,1,11}$	0.00	$F_{1,1,2,11}$	0.00

Table S8: The CcCR Force Constants for HOOH (cont.)

$F_{1,2,2,11}$	0.00	$F_{2,2,2,11}$	0.01	$F_{1,1,3,11}$	0.00	$F_{1,2,3,11}$	0.00	$F_{2,2,3,11}$	-0.00
$F_{1,3,3,11}$	-0.00	$F_{2,3,3,11}$	-0.00	$F_{3,3,3,11}$	-0.00	$F_{1,1,4,11}$	-0.01	$F_{1,2,4,11}$	-0.00
$F_{2,2,4,11}$	-0.01	$F_{1,3,4,11}$	-0.00	$F_{2,3,4,11}$	-0.00	$F_{3,3,4,11}$	0.01	$F_{1,4,4,11}$	0.01
$F_{2,4,4,11}$	0.01	$F_{3,4,4,11}$	0.00	$F_{4,4,4,11}$	-0.06	$F_{1,1,5,11}$	-0.00	$F_{1,2,5,11}$	-0.00
$F_{2,2,5,11}$	-0.01	$F_{1,3,5,11}$	-0.00	$F_{2,3,5,11}$	0.00	$F_{3,3,5,11}$	0.01	$F_{1,4,5,11}$	0.00
$F_{2,4,5,11}$	0.01	$F_{3,4,5,11}$	0.01	$F_{4,4,5,11}$	0.02	$F_{1,5,5,11}$	0.01	$F_{2,5,5,11}$	0.01
$F_{3,5,5,11}$	0.00	$F_{4,5,5,11}$	0.03	$F_{5,5,5,11}$	-0.13	$F_{1,1,6,11}$	-0.00	$F_{1,2,6,11}$	-0.00
$F_{2,2,6,11}$	-0.00	$F_{1,3,6,11}$	0.00	$F_{2,3,6,11}$	0.00	$F_{3,3,6,11}$	0.00	$F_{1,4,6,11}$	0.00
$F_{2,4,6,11}$	0.01	$F_{3,4,6,11}$	-0.01	$F_{4,4,6,11}$	-0.01	$F_{1,5,6,11}$	0.00	$F_{2,5,6,11}$	0.00
$F_{3,5,6,11}$	-0.01	$F_{4,5,6,11}$	-0.01	$F_{5,5,6,11}$	0.04	$F_{1,6,6,11}$	-0.01	$F_{2,6,6,11}$	-0.01
$F_{3,6,6,11}$	-0.01	$F_{4,6,6,11}$	-0.00	$F_{5,6,6,11}$	0.04	$F_{6,6,6,11}$	-0.03	$F_{1,1,7,11}$	0.00
$F_{1,2,7,11}$	0.00	$F_{2,2,7,11}$	0.01	$F_{1,3,7,11}$	0.00	$F_{2,3,7,11}$	0.00	$F_{3,3,7,11}$	-0.00
$F_{1,4,7,11}$	0.00	$F_{2,4,7,11}$	-0.01	$F_{3,4,7,11}$	-0.00	$F_{4,4,7,11}$	0.06	$F_{1,5,7,11}$	-0.00
$F_{2,5,7,11}$	-0.01	$F_{3,5,7,11}$	-0.00	$F_{4,5,7,11}$	-0.03	$F_{5,5,7,11}$	-0.02	$F_{1,6,7,11}$	-0.00
$F_{2,6,7,11}$	-0.00	$F_{3,6,7,11}$	0.00	$F_{4,6,7,11}$	0.01	$F_{5,6,7,11}$	-0.02	$F_{6,6,7,11}$	0.02
$F_{1,7,7,11}$	-0.00	$F_{2,7,7,11}$	0.01	$F_{3,7,7,11}$	0.00	$F_{4,7,7,11}$	-0.08	$F_{5,7,7,11}$	0.05
$F_{6,7,7,11}$	0.01	$F_{7,7,7,11}$	1.09	$F_{1,1,8,11}$	-0.00	$F_{1,2,8,11}$	0.00	$F_{2,2,8,11}$	0.01
$F_{1,3,8,11}$	-0.00	$F_{2,3,8,11}$	0.00	$F_{3,3,8,11}$	-0.00	$F_{1,4,8,11}$	0.00	$F_{2,4,8,11}$	-0.01
$F_{3,4,8,11}$	0.00	$F_{4,4,8,11}$	0.01	$F_{1,5,8,11}$	-0.00	$F_{2,5,8,11}$	-0.01	$F_{3,5,8,11}$	-0.00
$F_{4,5,8,11}$	-0.01	$F_{5,5,8,11}$	0.06	$F_{1,6,8,11}$	0.00	$F_{2,6,8,11}$	0.00	$F_{3,6,8,11}$	0.00
$F_{4,6,8,11}$	-0.00	$F_{5,6,8,11}$	-0.02	$F_{6,6,8,11}$	0.01	$F_{1,7,8,11}$	-0.00	$F_{2,7,8,11}$	0.01
$F_{3,7,8,11}$	-0.01	$F_{4,7,8,11}$	-0.02	$F_{5,7,8,11}$	0.06	$F_{6,7,8,11}$	0.01	$F_{7,7,8,11}$	0.35
$F_{1,8,8,11}$	0.00	$F_{2,8,8,11}$	0.01	$F_{3,8,8,11}$	0.00	$F_{4,8,8,11}$	-0.03	$F_{5,8,8,11}$	-0.06
$F_{6,8,8,11}$	-0.01	$F_{7,8,8,11}$	-0.69	$F_{8,8,8,11}$	0.23	$F_{1,1,9,11}$	-0.00	$F_{1,2,9,11}$	-0.00
$F_{2,2,9,11}$	0.00	$F_{1,3,9,11}$	0.00	$F_{2,3,9,11}$	0.00	$F_{3,3,9,11}$	-0.00	$F_{1,4,9,11}$	0.00
$F_{2,4,9,11}$	0.00	$F_{3,4,9,11}$	-0.00	$F_{4,4,9,11}$	0.01	$F_{1,5,9,11}$	-0.00	$F_{2,5,9,11}$	-0.00
$F_{3,5,9,11}$	-0.00	$F_{4,5,9,11}$	-0.02	$F_{5,5,9,11}$	-0.03	$F_{1,6,9,11}$	-0.00	$F_{2,6,9,11}$	-0.00
$F_{3,6,9,11}$	0.00	$F_{4,6,9,11}$	0.02	$F_{5,6,9,11}$	-0.02	$F_{6,6,9,11}$	0.04	$F_{1,7,9,11}$	-0.00
$F_{2,7,9,11}$	-0.01	$F_{3,7,9,11}$	0.00	$F_{4,7,9,11}$	0.01	$F_{5,7,9,11}$	0.06	$F_{6,7,9,11}$	-0.03
$F_{7,7,9,11}$	1.00	$F_{1,8,9,11}$	0.00	$F_{2,8,9,11}$	-0.00	$F_{3,8,9,11}$	-0.00	$F_{4,8,9,11}$	0.00
$F_{5,8,9,11}$	0.06	$F_{6,8,9,11}$	-0.02	$F_{7,8,9,11}$	0.24	$F_{8,8,9,11}$	-0.49	$F_{1,9,9,11}$	-0.00
$F_{2,9,9,11}$	-0.01	$F_{3,9,9,11}$	-0.00	$F_{4,9,9,11}$	-0.01	$F_{5,9,9,11}$	0.01	$F_{6,9,9,11}$	-0.07
$F_{7,9,9,11}$	0.58	$F_{8,9,9,11}$	0.17	$F_{9,9,9,11}$	0.13	$F_{1,1,10,11}$	-0.00	$F_{1,2,10,11}$	0.00
$F_{2,2,10,11}$	0.00	$F_{1,3,10,11}$	-0.00	$F_{2,3,10,11}$	-0.00	$F_{3,3,10,11}$	-0.00	$F_{1,4,10,11}$	-0.00
$F_{2,4,10,11}$	0.00	$F_{3,4,10,11}$	0.00	$F_{4,4,10,11}$	-0.00	$F_{1,5,10,11}$	-0.00	$F_{2,5,10,11}$	0.00
$F_{3,5,10,11}$	-0.00	$F_{4,5,10,11}$	0.01	$F_{5,5,10,11}$	-0.02	$F_{1,6,10,11}$	0.00	$F_{2,6,10,11}$	0.00
$F_{3,6,10,11}$	0.00	$F_{4,6,10,11}$	-0.00	$F_{5,6,10,11}$	0.03	$F_{6,6,10,11}$	-0.01	$F_{1,7,10,11}$	-0.00
$F_{2,7,10,11}$	-0.00	$F_{3,7,10,11}$	-0.00	$F_{4,7,10,11}$	0.02	$F_{5,7,10,11}$	-0.02	$F_{6,7,10,11}$	-0.01
$F_{7,7,10,11}$	-1.01	$F_{1,8,10,11}$	0.00	$F_{2,8,10,11}$	-0.00	$F_{3,8,10,11}$	0.01	$F_{4,8,10,11}$	0.01
$F_{5,8,10,11}$	-0.05	$F_{6,8,10,11}$	-0.00	$F_{7,8,10,11}$	-0.33	$F_{8,8,10,11}$	0.71	$F_{1,9,10,11}$	0.00
$F_{2,9,10,11}$	0.00	$F_{3,9,10,11}$	0.00	$F_{4,9,10,11}$	-0.02	$F_{5,9,10,11}$	-0.03	$F_{6,9,10,11}$	0.01
$F_{7,9,10,11}$	-1.00	$F_{8,9,10,11}$	-0.25	$F_{9,9,10,11}$	-0.57	$F_{1,10,10,11}$	0.00	$F_{2,10,10,11}$	0.00
$F_{3,10,10,11}$	0.00	$F_{4,10,10,11}$	-0.02	$F_{5,10,10,11}$	0.02	$F_{6,10,10,11}$	0.01	$F_{7,10,10,11}$	0.98
$F_{8,10,10,11}$	0.32	$F_{9,10,10,11}$	1.02	$F_{10,10,10,11}$	-0.97	$F_{1,1,11,11}$	0.00	$F_{1,2,11,11}$	0.00
$F_{2,2,11,11}$	-0.01	$F_{1,3,11,11}$	0.00	$F_{2,3,11,11}$	0.00	$F_{3,3,11,11}$	0.00	$F_{1,4,11,11}$	-0.00
$F_{2,4,11,11}$	0.01	$F_{3,4,11,11}$	-0.00	$F_{4,4,11,11}$	-0.04	$F_{1,5,11,11}$	-0.00	$F_{2,5,11,11}$	0.01
$F_{3,5,11,11}$	0.00	$F_{4,5,11,11}$	-0.03	$F_{5,5,11,11}$	0.06	$F_{1,6,11,11}$	-0.00	$F_{2,6,11,11}$	-0.00
$F_{3,6,11,11}$	-0.00	$F_{4,6,11,11}$	0.01	$F_{5,6,11,11}$	-0.02	$F_{6,6,11,11}$	-0.03	$F_{1,7,11,11}$	0.00
$F_{2,7,11,11}$	-0.01	$F_{3,7,11,11}$	0.00	$F_{4,7,11,11}$	0.06	$F_{5,7,11,11}$	-0.04	$F_{6,7,11,11}$	0.02

Table S9: The CcCR Force Constants for HOOH (cont.)

$F_{7,7,11,11}$	-0.42	$F_{1,8,11,11}$	0.00	$F_{2,8,11,11}$	-0.01	$F_{3,8,11,11}$	-0.00	$F_{4,8,11,11}$	0.05
$F_{5,8,11,11}$	0.00	$F_{6,8,11,11}$	0.03	$F_{7,8,11,11}$	0.62	$F_{8,8,11,11}$	-0.18	$F_{1,9,11,11}$	0.00
$F_{2,9,11,11}$	0.00	$F_{3,9,11,11}$	0.00	$F_{4,9,11,11}$	0.02	$F_{5,9,11,11}$	-0.03	$F_{6,9,11,11}$	0.04
$F_{7,9,11,11}$	-0.30	$F_{8,9,11,11}$	0.43	$F_{9,9,11,11}$	-0.18	$F_{1,10,11,11}$	-0.00	$F_{2,10,11,11}$	0.00
$F_{3,10,11,11}$	-0.00	$F_{4,10,11,11}$	-0.02	$F_{5,10,11,11}$	0.07	$F_{6,10,11,11}$	-0.03	$F_{7,10,11,11}$	0.35
$F_{8,10,11,11}$	-0.67	$F_{9,10,11,11}$	0.28	$F_{10,10,11,11}$	-0.34	$F_{1,11,11,11}$	0.00	$F_{2,11,11,11}$	0.01
$F_{3,11,11,11}$	-0.00	$F_{4,11,11,11}$	-0.03	$F_{5,11,11,11}$	-0.07	$F_{6,11,11,11}$	-0.00	$F_{7,11,11,11}$	-0.57
$F_{8,11,11,11}$	0.18	$F_{9,11,11,11}$	-0.40	$F_{10,11,11,11}$	0.59	$F_{11,11,11,11}$	-0.11	$F_{1,1,1,12}$	0.00
$F_{1,1,2,12}$	-0.00	$F_{1,2,2,12}$	0.00	$F_{2,2,2,12}$	0.00	$F_{1,1,3,12}$	0.00	$F_{1,2,3,12}$	-0.00
$F_{2,2,3,12}$	0.00	$F_{1,3,3,12}$	-0.00	$F_{2,3,3,12}$	0.00	$F_{3,3,3,12}$	-0.00	$F_{1,1,4,12}$	-0.00
$F_{1,2,4,12}$	0.00	$F_{2,2,4,12}$	-0.00	$F_{1,3,4,12}$	-0.00	$F_{2,3,4,12}$	-0.00	$F_{3,3,4,12}$	0.00
$F_{1,4,4,12}$	0.00	$F_{2,4,4,12}$	-0.00	$F_{3,4,4,12}$	0.01	$F_{4,4,4,12}$	0.00	$F_{1,1,5,12}$	0.00
$F_{1,2,5,12}$	-0.01	$F_{2,2,5,12}$	0.00	$F_{1,3,5,12}$	-0.00	$F_{2,3,5,12}$	0.00	$F_{3,3,5,12}$	-0.00
$F_{1,4,5,12}$	-0.00	$F_{2,4,5,12}$	0.01	$F_{3,4,5,12}$	0.00	$F_{4,4,5,12}$	0.01	$F_{1,5,5,12}$	0.02
$F_{2,5,5,12}$	-0.00	$F_{3,5,5,12}$	-0.01	$F_{4,5,5,12}$	-0.03	$F_{5,5,5,12}$	0.08	$F_{1,1,6,12}$	-0.00
$F_{1,2,6,12}$	0.00	$F_{2,2,6,12}$	0.00	$F_{1,3,6,12}$	0.00	$F_{2,3,6,12}$	-0.00	$F_{3,3,6,12}$	0.00
$F_{1,4,6,12}$	0.00	$F_{2,4,6,12}$	0.00	$F_{3,4,6,12}$	-0.00	$F_{4,4,6,12}$	-0.01	$F_{1,5,6,12}$	0.00
$F_{2,5,6,12}$	-0.00	$F_{3,5,6,12}$	0.00	$F_{4,5,6,12}$	-0.01	$F_{5,5,6,12}$	0.04	$F_{1,6,6,12}$	-0.00
$F_{2,6,6,12}$	0.00	$F_{3,6,6,12}$	-0.00	$F_{4,6,6,12}$	-0.00	$F_{5,6,6,12}$	-0.02	$F_{6,6,6,12}$	-0.01
$F_{1,1,7,12}$	-0.00	$F_{1,2,7,12}$	-0.00	$F_{2,2,7,12}$	0.00	$F_{1,3,7,12}$	0.00	$F_{2,3,7,12}$	0.00
$F_{3,3,7,12}$	-0.00	$F_{1,4,7,12}$	0.00	$F_{2,4,7,12}$	0.00	$F_{3,4,7,12}$	-0.01	$F_{4,4,7,12}$	-0.01
$F_{1,5,7,12}$	-0.01	$F_{2,5,7,12}$	-0.00	$F_{3,5,7,12}$	-0.00	$F_{4,5,7,12}$	-0.01	$F_{5,5,7,12}$	0.05
$F_{1,6,7,12}$	-0.00	$F_{2,6,7,12}$	-0.00	$F_{3,6,7,12}$	0.00	$F_{4,6,7,12}$	0.01	$F_{5,6,7,12}$	0.01
$F_{6,6,7,12}$	0.00	$F_{1,7,7,12}$	0.00	$F_{2,7,7,12}$	-0.00	$F_{3,7,7,12}$	0.01	$F_{4,7,7,12}$	0.04
$F_{5,7,7,12}$	-0.00	$F_{6,7,7,12}$	0.01	$F_{7,7,7,12}$	-2.33	$F_{1,1,8,12}$	-0.00	$F_{1,2,8,12}$	0.00
$F_{2,2,8,12}$	-0.00	$F_{1,3,8,12}$	0.00	$F_{2,3,8,12}$	0.00	$F_{3,3,8,12}$	0.00	$F_{1,4,8,12}$	-0.00
$F_{2,4,8,12}$	0.00	$F_{3,4,8,12}$	-0.00	$F_{4,4,8,12}$	-0.01	$F_{1,5,8,12}$	-0.01	$F_{2,5,8,12}$	0.00
$F_{3,5,8,12}$	0.00	$F_{4,5,8,12}$	-0.01	$F_{5,5,8,12}$	-0.06	$F_{1,6,8,12}$	-0.00	$F_{2,6,8,12}$	-0.00
$F_{3,6,8,12}$	-0.00	$F_{4,6,8,12}$	0.01	$F_{5,6,8,12}$	-0.02	$F_{6,6,8,12}$	0.02	$F_{1,7,8,12}$	0.01
$F_{2,7,8,12}$	-0.01	$F_{3,7,8,12}$	0.00	$F_{4,7,8,12}$	0.02	$F_{5,7,8,12}$	-0.01	$F_{6,7,8,12}$	-0.01
$F_{7,7,8,12}$	1.01	$F_{1,8,8,12}$	0.01	$F_{2,8,8,12}$	-0.00	$F_{3,8,8,12}$	-0.01	$F_{4,8,8,12}$	0.01
$F_{5,8,8,12}$	0.09	$F_{6,8,8,12}$	0.01	$F_{7,8,8,12}$	0.26	$F_{8,8,8,12}$	-0.58	$F_{1,1,9,12}$	0.00
$F_{1,2,9,12}$	0.00	$F_{2,2,9,12}$	-0.00	$F_{1,3,9,12}$	0.00	$F_{2,3,9,12}$	-0.00	$F_{3,3,9,12}$	0.00
$F_{1,4,9,12}$	-0.00	$F_{2,4,9,12}$	0.00	$F_{3,4,9,12}$	-0.00	$F_{4,4,9,12}$	0.00	$F_{1,5,9,12}$	0.00
$F_{2,5,9,12}$	0.00	$F_{3,5,9,12}$	0.00	$F_{4,5,9,12}$	0.00	$F_{5,5,9,12}$	-0.01	$F_{1,6,9,12}$	-0.00
$F_{2,6,9,12}$	-0.00	$F_{3,6,9,12}$	-0.00	$F_{4,6,9,12}$	0.02	$F_{5,6,9,12}$	0.02	$F_{6,6,9,12}$	0.02
$F_{1,7,9,12}$	0.00	$F_{2,7,9,12}$	-0.01	$F_{3,7,9,12}$	0.00	$F_{4,7,9,12}$	0.02	$F_{5,7,9,12}$	0.01
$F_{6,7,9,12}$	-0.03	$F_{7,7,9,12}$	-1.65	$F_{1,8,9,12}$	-0.00	$F_{2,8,9,12}$	-0.01	$F_{3,8,9,12}$	-0.00
$F_{4,8,9,12}$	-0.00	$F_{5,8,9,12}$	-0.00	$F_{6,8,9,12}$	-0.05	$F_{7,8,9,12}$	0.56	$F_{8,8,9,12}$	0.15
$F_{1,9,9,12}$	-0.00	$F_{2,9,9,12}$	0.01	$F_{3,9,9,12}$	-0.00	$F_{4,9,9,12}$	-0.04	$F_{5,9,9,12}$	0.02
$F_{6,9,9,12}$	-0.02	$F_{7,9,9,12}$	0.12	$F_{8,9,9,12}$	0.03	$F_{9,9,9,12}$	0.99	$F_{1,1,10,12}$	0.00
$F_{1,2,10,12}$	0.00	$F_{2,2,10,12}$	-0.00	$F_{1,3,10,12}$	-0.00	$F_{2,3,10,12}$	-0.00	$F_{3,3,10,12}$	0.00
$F_{1,4,10,12}$	-0.00	$F_{2,4,10,12}$	-0.00	$F_{3,4,10,12}$	0.00	$F_{4,4,10,12}$	0.01	$F_{1,5,10,12}$	0.00
$F_{2,5,10,12}$	0.00	$F_{3,5,10,12}$	0.00	$F_{4,5,10,12}$	-0.00	$F_{5,5,10,12}$	-0.04	$F_{1,6,10,12}$	0.00
$F_{2,6,10,12}$	0.00	$F_{3,6,10,12}$	-0.00	$F_{4,6,10,12}$	0.00	$F_{5,6,10,12}$	-0.00	$F_{6,6,10,12}$	-0.00
$F_{1,7,10,12}$	0.00	$F_{2,7,10,12}$	0.00	$F_{3,7,10,12}$	-0.00	$F_{4,7,10,12}$	-0.03	$F_{5,7,10,12}$	0.02
$F_{6,7,10,12}$	-0.01	$F_{7,7,10,12}$	2.29	$F_{1,8,10,12}$	-0.00	$F_{2,8,10,12}$	0.00	$F_{3,8,10,12}$	-0.00
$F_{4,8,10,12}$	-0.01	$F_{5,8,10,12}$	0.03	$F_{6,8,10,12}$	0.00	$F_{7,8,10,12}$	-1.04	$F_{8,8,10,12}$	-0.28
$F_{1,9,10,12}$	-0.00	$F_{2,9,10,12}$	0.00	$F_{3,9,10,12}$	-0.00	$F_{4,9,10,12}$	-0.01	$F_{5,9,10,12}$	-0.01

Table S10: The CcCR Force Constants for HOOH (cont.)

$F_{6,9,10,12}$	0.01	$F_{7,9,10,12}$	1.63	$F_{8,9,10,12}$	-0.55	$F_{9,9,10,12}$	-0.07	$F_{1,10,10,12}$	-0.00
$F_{2,10,10,12}$	-0.00	$F_{3,10,10,12}$	0.00	$F_{4,10,10,12}$	0.01	$F_{5,10,10,12}$	-0.02	$F_{6,10,10,12}$	0.01
$F_{7,10,10,12}$	-2.27	$F_{8,10,10,12}$	1.06	$F_{9,10,10,12}$	-1.61	$F_{10,10,10,12}$	2.25	$F_{1,1,11,12}$	0.00
$F_{1,2,11,12}$	0.00	$F_{2,2,11,12}$	-0.00	$F_{1,3,11,12}$	-0.00	$F_{2,3,11,12}$	-0.00	$F_{3,3,11,12}$	-0.00
$F_{1,4,11,12}$	0.00	$F_{2,4,11,12}$	-0.00	$F_{3,4,11,12}$	0.00	$F_{4,4,11,12}$	-0.01	$F_{1,5,11,12}$	-0.00
$F_{2,5,11,12}$	-0.00	$F_{3,5,11,12}$	0.00	$F_{4,5,11,12}$	0.03	$F_{5,5,11,12}$	-0.02	$F_{1,6,11,12}$	0.00
$F_{2,6,11,12}$	0.00	$F_{3,6,11,12}$	0.00	$F_{4,6,11,12}$	-0.01	$F_{5,6,11,12}$	-0.01	$F_{6,6,11,12}$	-0.00
$F_{1,7,11,12}$	0.00	$F_{2,7,11,12}$	0.00	$F_{3,7,11,12}$	-0.00	$F_{4,7,11,12}$	-0.01	$F_{5,7,11,12}$	-0.03
$F_{6,7,11,12}$	0.01	$F_{7,7,11,12}$	-1.01	$F_{1,8,11,12}$	-0.00	$F_{2,8,11,12}$	-0.00	$F_{3,8,11,12}$	0.00
$F_{4,8,11,12}$	-0.00	$F_{5,8,11,12}$	-0.03	$F_{6,8,11,12}$	0.01	$F_{7,8,11,12}$	-0.24	$F_{8,8,11,12}$	0.49
$F_{1,9,11,12}$	0.00	$F_{2,9,11,12}$	0.00	$F_{3,9,11,12}$	0.00	$F_{4,9,11,12}$	-0.00	$F_{5,9,11,12}$	0.01
$F_{6,9,11,12}$	0.03	$F_{7,9,11,12}$	-0.56	$F_{8,9,11,12}$	-0.15	$F_{9,9,11,12}$	-0.05	$F_{1,10,11,12}$	-0.00
$F_{2,10,11,12}$	-0.00	$F_{3,10,11,12}$	-0.00	$F_{4,10,11,12}$	0.02	$F_{5,10,11,12}$	0.00	$F_{6,10,11,12}$	-0.00
$F_{7,10,11,12}$	1.02	$F_{8,10,11,12}$	0.25	$F_{9,10,11,12}$	0.56	$F_{10,10,11,12}$	-1.04	$F_{1,11,11,12}$	0.00
$F_{2,11,11,12}$	0.00	$F_{3,11,11,12}$	0.00	$F_{4,11,11,12}$	-0.03	$F_{5,11,11,12}$	0.05	$F_{6,11,11,12}$	-0.00
$F_{7,11,11,12}$	0.27	$F_{8,11,11,12}$	-0.46	$F_{9,11,11,12}$	0.14	$F_{10,11,11,12}$	-0.25	$F_{11,11,11,12}$	0.40
$F_{1,1,12,12}$	-0.00	$F_{1,2,12,12}$	-0.00	$F_{2,2,12,12}$	0.00	$F_{1,3,12,12}$	-0.00	$F_{2,3,12,12}$	0.00
$F_{3,3,12,12}$	0.00	$F_{1,4,12,12}$	0.00	$F_{2,4,12,12}$	-0.00	$F_{3,4,12,12}$	0.00	$F_{4,4,12,12}$	0.01
$F_{1,5,12,12}$	0.00	$F_{2,5,12,12}$	-0.00	$F_{3,5,12,12}$	-0.00	$F_{4,5,12,12}$	0.00	$F_{5,5,12,12}$	-0.02
$F_{1,6,12,12}$	0.00	$F_{2,6,12,12}$	0.00	$F_{3,6,12,12}$	0.00	$F_{4,6,12,12}$	-0.02	$F_{5,6,12,12}$	0.00
$F_{6,6,12,12}$	-0.00	$F_{1,7,12,12}$	-0.00	$F_{2,7,12,12}$	0.01	$F_{3,7,12,12}$	-0.00	$F_{4,7,12,12}$	-0.02
$F_{5,7,12,12}$	-0.01	$F_{6,7,12,12}$	0.03	$F_{7,7,12,12}$	1.63	$F_{1,8,12,12}$	0.00	$F_{2,8,12,12}$	0.01
$F_{3,8,12,12}$	0.00	$F_{4,8,12,12}$	-0.01	$F_{5,8,12,12}$	0.02	$F_{6,8,12,12}$	0.02	$F_{7,8,12,12}$	-0.55
$F_{8,8,12,12}$	-0.16	$F_{1,9,12,12}$	0.00	$F_{2,9,12,12}$	-0.00	$F_{3,9,12,12}$	0.00	$F_{4,9,12,12}$	0.02
$F_{5,9,12,12}$	-0.03	$F_{6,9,12,12}$	0.01	$F_{7,9,12,12}$	-0.09	$F_{8,9,12,12}$	0.02	$F_{9,9,12,12}$	-0.97
$F_{1,10,12,12}$	0.00	$F_{2,10,12,12}$	-0.00	$F_{3,10,12,12}$	0.00	$F_{4,10,12,12}$	0.01	$F_{5,10,12,12}$	0.01
$F_{6,10,12,12}$	-0.01	$F_{7,10,12,12}$	-1.62	$F_{8,10,12,12}$	0.55	$F_{9,10,12,12}$	0.07	$F_{10,10,12,12}$	1.60
$F_{1,11,12,12}$	-0.00	$F_{2,11,12,12}$	-0.00	$F_{3,11,12,12}$	-0.00	$F_{4,11,12,12}$	0.01	$F_{5,11,12,12}$	0.01
$F_{6,11,12,12}$	-0.03	$F_{7,11,12,12}$	0.55	$F_{8,11,12,12}$	0.13	$F_{9,11,12,12}$	0.02	$F_{10,11,12,12}$	-0.56
$F_{11,11,12,12}$	-0.13	$F_{1,12,12,12}$	-0.00	$F_{2,12,12,12}$	0.00	$F_{3,12,12,12}$	-0.00	$F_{4,12,12,12}$	-0.00
$F_{5,12,12,12}$	0.03	$F_{6,12,12,12}$	-0.00	$F_{7,12,12,12}$	0.06	$F_{8,12,12,12}$	-0.04	$F_{9,12,12,12}$	0.96
$F_{10,12,12,12}$	-0.06	$F_{11,12,12,12}$	0.01	$F_{12,12,12,12}$	-0.95				

Table S11: The CcCR Force Constants for HOSH (cont.)

$F_{5,8,9}$	-0.0240	$F_{6,8,9}$	0.0387	$F_{7,8,9}$	0.0057	$F_{8,8,9}$	0.1844
$F_{1,9,9}$	0.0039	$F_{2,9,9}$	0.0017	$F_{3,9,9}$	0.0481	$F_{4,9,9}$	-0.0045
$F_{5,9,9}$	0.0457	$F_{6,9,9}$	0.7295	$F_{7,9,9}$	0.0020	$F_{8,9,9}$	-0.0133
$F_{9,9,9}$	-0.8199	$F_{1,1,10}$	0.0006	$F_{1,2,10}$	0.0020	$F_{2,2,10}$	-0.0007
$F_{1,3,10}$	-0.0004	$F_{2,3,10}$	0.0002	$F_{3,3,10}$	0.0000	$F_{1,4,10}$	-0.0010
$F_{2,4,10}$	-0.0032	$F_{3,4,10}$	0.0008	$F_{4,4,10}$	0.0012	$F_{1,5,10}$	-0.0022
$F_{2,5,10}$	0.0026	$F_{3,5,10}$	-0.0010	$F_{4,5,10}$	-0.0062	$F_{5,5,10}$	-0.0050
$F_{1,6,10}$	0.0002	$F_{2,6,10}$	0.0036	$F_{3,6,10}$	-0.0000	$F_{4,6,10}$	0.0055
$F_{5,6,10}$	-0.0052	$F_{6,6,10}$	0.0021	$F_{1,7,10}$	0.0004	$F_{2,7,10}$	0.0016
$F_{3,7,10}$	-0.0006	$F_{4,7,10}$	0.0000	$F_{5,7,10}$	0.0098	$F_{6,7,10}$	-0.0072
$F_{7,7,10}$	-0.0032	$F_{1,8,10}$	0.0004	$F_{2,8,10}$	-0.0027	$F_{3,8,10}$	0.0017
$F_{4,8,10}$	0.0127	$F_{5,8,10}$	0.0033	$F_{6,8,10}$	0.0016	$F_{7,8,10}$	-0.1155
$F_{8,8,10}$	0.0077	$F_{1,9,10}$	-0.0001	$F_{2,9,10}$	-0.0053	$F_{3,9,10}$	0.0023
$F_{4,9,10}$	0.0059	$F_{5,9,10}$	0.0076	$F_{6,9,10}$	-0.0025	$F_{7,9,10}$	0.0120
$F_{8,9,10}$	-0.0047	$F_{9,9,10}$	-0.0015	$F_{1,10,10}$	0.0001	$F_{2,10,10}$	-0.0004
$F_{3,10,10}$	0.0002	$F_{4,10,10}$	-0.0002	$F_{5,10,10}$	-0.0014	$F_{6,10,10}$	0.0015
$F_{7,10,10}$	0.0027	$F_{8,10,10}$	0.1024	$F_{9,10,10}$	-0.0178	$F_{10,10,10}$	-0.0026
$F_{1,1,11}$	0.0000	$F_{1,2,11}$	-0.0005	$F_{2,2,11}$	-0.0008	$F_{1,3,11}$	0.0001
$F_{2,3,11}$	-0.0008	$F_{3,3,11}$	0.0011	$F_{1,4,11}$	0.0001	$F_{2,4,11}$	0.0003
$F_{3,4,11}$	0.0002	$F_{4,4,11}$	-0.0012	$F_{1,5,11}$	0.0007	$F_{2,5,11}$	0.0009
$F_{3,5,11}$	0.0003	$F_{4,5,11}$	-0.0009	$F_{5,5,11}$	0.0007	$F_{1,6,11}$	-0.0015
$F_{2,6,11}$	-0.0002	$F_{3,6,11}$	-0.0009	$F_{4,6,11}$	0.0010	$F_{5,6,11}$	0.0049
$F_{6,6,11}$	0.0142	$F_{1,7,11}$	0.0001	$F_{2,7,11}$	-0.0006	$F_{3,7,11}$	0.0007
$F_{4,7,11}$	0.0044	$F_{5,7,11}$	0.0011	$F_{6,7,11}$	0.0005	$F_{7,7,11}$	-0.1087
$F_{1,8,11}$	-0.0015	$F_{2,8,11}$	-0.0007	$F_{3,8,11}$	0.0006	$F_{4,8,11}$	0.0016
$F_{5,8,11}$	-0.0066	$F_{6,8,11}$	0.0088	$F_{7,8,11}$	0.0082	$F_{8,8,11}$	0.7203
$F_{1,9,11}$	0.0009	$F_{2,9,11}$	0.0001	$F_{3,9,11}$	0.0007	$F_{4,9,11}$	-0.0002
$F_{5,9,11}$	-0.0176	$F_{6,9,11}$	-0.0104	$F_{7,9,11}$	-0.0031	$F_{8,9,11}$	-0.1481
$F_{9,9,11}$	-0.0342	$F_{1,10,11}$	-0.0002	$F_{2,10,11}$	0.0008	$F_{3,10,11}$	-0.0009
$F_{4,10,11}$	-0.0034	$F_{5,10,11}$	-0.0009	$F_{6,10,11}$	0.0001	$F_{7,10,11}$	0.1042
$F_{8,10,11}$	-0.0083	$F_{9,10,11}$	0.0024	$F_{10,10,11}$	-0.1006	$F_{1,11,11}$	0.0013
$F_{2,11,11}$	0.0005	$F_{3,11,11}$	-0.0002	$F_{4,11,11}$	-0.0010	$F_{5,11,11}$	0.0049
$F_{6,11,11}$	-0.0136	$F_{7,11,11}$	-0.0087	$F_{8,11,11}$	-0.7131	$F_{9,11,11}$	0.1657
$F_{10,11,11}$	0.0084	$F_{11,11,11}$	0.7077	$F_{1,1,12}$	0.0015	$F_{1,2,12}$	0.0017
$F_{2,2,12}$	0.0011	$F_{1,3,12}$	-0.0002	$F_{2,3,12}$	-0.0033	$F_{3,3,12}$	-0.0016
$F_{1,4,12}$	-0.0016	$F_{2,4,12}$	-0.0024	$F_{3,4,12}$	-0.0008	$F_{4,4,12}$	0.0054
$F_{1,5,12}$	-0.0013	$F_{2,5,12}$	-0.0013	$F_{3,5,12}$	0.0051	$F_{4,5,12}$	0.0014
$F_{5,5,12}$	0.0014	$F_{1,6,12}$	0.0013	$F_{2,6,12}$	0.0030	$F_{3,6,12}$	0.0011
$F_{4,6,12}$	0.0007	$F_{5,6,12}$	0.0264	$F_{6,6,12}$	-0.0333	$F_{1,7,12}$	-0.0001
$F_{2,7,12}$	-0.0008	$F_{3,7,12}$	0.0033	$F_{4,7,12}$	0.0084	$F_{5,7,12}$	0.0012
$F_{6,7,12}$	-0.0024	$F_{7,7,12}$	-0.0041	$F_{1,8,12}$	-0.0010	$F_{2,8,12}$	-0.0007
$F_{3,8,12}$	-0.0010	$F_{4,8,12}$	0.0019	$F_{5,8,12}$	-0.0124	$F_{6,8,12}$	-0.0264
$F_{7,8,12}$	-0.0024	$F_{8,8,12}$	-0.1256	$F_{1,9,12}$	-0.0020	$F_{2,9,12}$	0.0007
$F_{3,9,12}$	-0.0004	$F_{4,9,12}$	0.0019	$F_{5,9,12}$	-0.0170	$F_{6,9,12}$	0.0038
$F_{7,9,12}$	-0.0015	$F_{8,9,12}$	-0.0276	$F_{9,9,12}$	0.0423	$F_{1,10,12}$	0.0002
$F_{2,10,12}$	0.0015	$F_{3,10,12}$	-0.0023	$F_{4,10,12}$	-0.0122	$F_{5,10,12}$	-0.0013
$F_{6,10,12}$	0.0004	$F_{7,10,12}$	-0.0042	$F_{8,10,12}$	0.0014	$F_{9,10,12}$	0.0017
$F_{10,10,12}$	0.0161	$F_{1,11,12}$	0.0006	$F_{2,11,12}$	0.0009	$F_{3,11,12}$	-0.0009
$F_{4,11,12}$	-0.0009	$F_{5,11,12}$	0.0123	$F_{6,11,12}$	-0.0029	$F_{7,11,12}$	0.0020
$F_{8,11,12}$	0.1387	$F_{9,11,12}$	0.0439	$F_{10,11,12}$	-0.0016	$F_{11,11,12}$	-0.1519
$F_{1,12,12}$	0.0010	$F_{2,12,12}$	-0.0004	$F_{3,12,12}$	0.0008	$F_{4,12,12}$	-0.0019
$F_{5,12,12}$	-0.0146	$F_{6,12,12}$	0.0284	$F_{7,12,12}$	0.0007	$F_{8,12,12}$	0.0550
$F_{9,12,12}$	-0.0457	$F_{10,12,12}$	0.0002	$F_{11,12,12}$	-0.0401	$F_{12,12,12}$	0.0165
$F_{1,1,1,1}$	4.27	$F_{1,1,1,2}$	-0.12	$F_{1,1,2,2}$	-1.23	$F_{1,2,2,2}$	0.06
$F_{2,2,2,2}$	0.46	$F_{1,1,1,3}$	2.75	$F_{1,1,2,3}$	-0.05	$F_{1,2,2,3}$	-0.47
$F_{2,2,2,3}$	0.02	$F_{1,1,3,3}$	0.11	$F_{1,2,3,3}$	-0.00	$F_{2,2,3,3}$	-0.04
$F_{1,3,3,3}$	-0.81	$F_{2,3,3,3}$	0.01	$F_{3,3,3,3}$	-0.39	$F_{1,1,1,4}$	-4.29
$F_{1,1,2,4}$	0.12	$F_{1,2,2,4}$	1.24	$F_{2,2,2,4}$	-0.06	$F_{1,1,3,4}$	-2.76
$F_{1,2,3,4}$	0.05	$F_{2,2,3,4}$	0.45	$F_{1,3,3,4}$	-0.08	$F_{2,3,3,4}$	0.00
$F_{3,3,3,4}$	0.81	$F_{1,1,4,4}$	4.31	$F_{1,2,4,4}$	-0.12	$F_{2,2,4,4}$	-1.26
$F_{1,3,4,4}$	2.78	$F_{2,3,4,4}$	-0.06	$F_{3,3,4,4}$	0.03	$F_{1,4,4,4}$	-4.32
$F_{2,4,4,4}$	0.11	$F_{3,4,4,4}$	-2.84	$F_{4,4,4,4}$	4.35	$F_{1,1,1,5}$	0.12
$F_{1,1,2,5}$	1.24	$F_{1,2,2,5}$	-0.06	$F_{2,2,2,5}$	-0.46	$F_{1,1,3,5}$	0.05
$F_{1,2,3,5}$	0.45	$F_{2,2,3,5}$	-0.02	$F_{1,3,3,5}$	0.00	$F_{2,3,3,5}$	0.02
$F_{3,3,3,5}$	-0.01	$F_{1,1,4,5}$	-0.12	$F_{1,2,4,5}$	-1.25	$F_{2,2,4,5}$	0.06
$F_{1,3,4,5}$	-0.05	$F_{2,3,4,5}$	-0.44	$F_{3,3,4,5}$	-0.00	$F_{1,4,4,5}$	0.12
$F_{2,4,4,5}$	1.29	$F_{3,4,4,5}$	0.05	$F_{4,4,4,5}$	-0.11	$F_{1,1,5,5}$	-1.24
$F_{1,2,5,5}$	0.06	$F_{2,2,5,5}$	0.48	$F_{1,3,5,5}$	-0.43	$F_{2,3,5,5}$	0.02
$F_{3,3,5,5}$	-0.03	$F_{1,4,5,5}$	1.26	$F_{2,4,5,5}$	-0.06	$F_{3,4,5,5}$	0.41
$F_{4,4,5,5}$	-1.30	$F_{1,5,5,5}$	-0.07	$F_{2,5,5,5}$	-0.49	$F_{3,5,5,5}$	-0.03
$F_{4,5,5,5}$	0.07	$F_{5,5,5,5}$	0.53	$F_{1,1,1,6}$	-2.75	$F_{1,1,2,6}$	0.06
$F_{1,2,2,6}$	0.50	$F_{2,2,2,6}$	-0.02	$F_{1,1,3,6}$	-0.12	$F_{1,2,3,6}$	0.00
$F_{2,2,3,6}$	0.03	$F_{1,3,3,6}$	0.86	$F_{2,3,3,6}$	-0.02	$F_{3,3,3,6}$	0.44
$F_{1,1,4,6}$	2.76	$F_{1,2,4,6}$	-0.06	$F_{2,2,4,6}$	-0.49	$F_{1,3,4,6}$	0.09

Table S12: The CcCR Force Constants for HOSH (cont.)

$F_{2,3,4,6}$	-0.00	$F_{3,3,4,6}$	-0.84	$F_{1,4,4,6}$	-2.78	$F_{2,4,4,6}$	0.06
$F_{3,4,4,6}$	-0.07	$F_{4,4,4,6}$	2.83	$F_{1,1,5,6}$	-0.06	$F_{1,2,5,6}$	-0.48
$F_{2,2,5,6}$	0.03	$F_{1,3,5,6}$	-0.00	$F_{2,3,5,6}$	-0.03	$F_{3,3,5,6}$	0.02
$F_{1,4,5,6}$	0.06	$F_{2,4,5,6}$	0.47	$F_{3,4,5,6}$	0.00	$F_{4,4,5,6}$	-0.05
$F_{1,5,5,6}$	0.46	$F_{2,5,5,6}$	-0.04	$F_{3,5,5,6}$	0.04	$F_{4,5,5,6}$	-0.43
$F_{5,5,5,6}$	0.04	$F_{1,1,6,6}$	0.08	$F_{1,2,6,6}$	0.00	$F_{2,2,6,6}$	-0.03
$F_{1,3,6,6}$	-0.91	$F_{2,3,6,6}$	0.02	$F_{3,3,6,6}$	-0.47	$F_{1,4,6,6}$	-0.08
$F_{2,4,6,6}$	0.00	$F_{3,4,6,6}$	0.89	$F_{4,4,6,6}$	-0.20	$F_{1,5,6,6}$	-0.00
$F_{2,5,6,6}$	0.02	$F_{3,5,6,6}$	-0.02	$F_{4,5,6,6}$	-0.01	$F_{5,5,6,6}$	-0.25
$F_{1,6,6,6}$	0.99	$F_{2,6,6,6}$	-0.03	$F_{3,6,6,6}$	0.52	$F_{4,6,6,6}$	-1.06
$F_{5,6,6,6}$	0.07	$F_{6,6,6,6}$	1.25	$F_{1,1,1,7}$	0.02	$F_{1,1,2,7}$	-0.00
$F_{1,2,2,7}$	-0.01	$F_{2,2,2,7}$	0.01	$F_{1,1,3,7}$	0.01	$F_{1,2,3,7}$	-0.01
$F_{2,2,3,7}$	0.02	$F_{1,3,3,7}$	-0.03	$F_{2,3,3,7}$	-0.00	$F_{3,3,3,7}$	-0.00
$F_{1,1,4,7}$	-0.02	$F_{1,2,4,7}$	-0.00	$F_{2,2,4,7}$	0.03	$F_{1,3,4,7}$	-0.02
$F_{2,3,4,7}$	0.00	$F_{3,3,4,7}$	0.04	$F_{1,4,4,7}$	0.00	$F_{2,4,4,7}$	0.01
$F_{3,4,4,7}$	0.06	$F_{4,4,4,7}$	-0.03	$F_{1,1,5,7}$	0.00	$F_{1,2,5,7}$	0.02
$F_{2,2,5,7}$	-0.01	$F_{1,3,5,7}$	0.01	$F_{2,3,5,7}$	-0.01	$F_{3,3,5,7}$	0.00
$F_{1,4,5,7}$	0.00	$F_{2,4,5,7}$	-0.03	$F_{3,4,5,7}$	-0.00	$F_{4,4,5,7}$	-0.01
$F_{1,5,5,7}$	-0.02	$F_{2,5,5,7}$	0.01	$F_{3,5,5,7}$	0.02	$F_{4,5,5,7}$	0.04
$F_{5,5,5,7}$	-0.02	$F_{1,1,6,7}$	-0.01	$F_{1,2,6,7}$	0.00	$F_{2,2,6,7}$	-0.01
$F_{1,3,6,7}$	0.02	$F_{2,3,6,7}$	0.01	$F_{3,3,6,7}$	-0.01	$F_{1,4,6,7}$	0.02
$F_{2,4,6,7}$	0.00	$F_{3,4,6,7}$	-0.02	$F_{4,4,6,7}$	-0.06	$F_{1,5,6,7}$	-0.00
$F_{2,5,6,7}$	0.01	$F_{3,5,6,7}$	-0.01	$F_{4,5,6,7}$	0.01	$F_{5,5,6,7}$	-0.02
$F_{1,6,6,7}$	-0.00	$F_{2,6,6,7}$	-0.01	$F_{3,6,6,7}$	0.01	$F_{4,6,6,7}$	0.26
$F_{5,6,6,7}$	0.02	$F_{6,6,6,7}$	0.07	$F_{1,1,7,7}$	-0.00	$F_{1,2,7,7}$	0.01
$F_{2,2,7,7}$	-0.02	$F_{1,3,7,7}$	0.02	$F_{2,3,7,7}$	0.00	$F_{3,3,7,7}$	-0.01
$F_{1,4,7,7}$	0.02	$F_{2,4,7,7}$	-0.01	$F_{3,4,7,7}$	-0.03	$F_{4,4,7,7}$	0.02
$F_{1,5,7,7}$	-0.01	$F_{2,5,7,7}$	0.02	$F_{3,5,7,7}$	-0.00	$F_{4,5,7,7}$	0.01
$F_{5,5,7,7}$	-0.03	$F_{1,6,7,7}$	-0.02	$F_{2,6,7,7}$	-0.01	$F_{3,6,7,7}$	-0.01
$F_{4,6,7,7}$	0.03	$F_{5,6,7,7}$	-0.01	$F_{6,6,7,7}$	-0.24	$F_{1,7,7,7}$	-0.02
$F_{2,7,7,7}$	0.01	$F_{3,7,7,7}$	0.01	$F_{4,7,7,7}$	-0.05	$F_{5,7,7,7}$	-0.01
$F_{6,7,7,7}$	-0.01	$F_{7,7,7,7}$	0.21	$F_{1,1,1,8}$	0.00	$F_{1,1,2,8}$	-0.01
$F_{1,2,2,8}$	0.00	$F_{2,2,2,8}$	0.00	$F_{1,1,3,8}$	-0.00	$F_{1,2,3,8}$	0.02
$F_{2,2,3,8}$	-0.00	$F_{1,3,3,8}$	-0.00	$F_{2,3,3,8}$	0.02	$F_{3,3,3,8}$	-0.01
$F_{1,1,4,8}$	-0.00	$F_{1,2,4,8}$	0.01	$F_{2,2,4,8}$	-0.00	$F_{1,3,4,8}$	0.00
$F_{2,3,4,8}$	-0.02	$F_{3,3,4,8}$	-0.00	$F_{1,4,4,8}$	0.00	$F_{2,4,4,8}$	-0.02
$F_{3,4,4,8}$	-0.00	$F_{4,4,4,8}$	-0.00	$F_{1,1,5,8}$	-0.00	$F_{1,2,5,8}$	-0.01
$F_{2,2,5,8}$	-0.01	$F_{1,3,5,8}$	-0.02	$F_{2,3,5,8}$	0.00	$F_{3,3,5,8}$	0.01
$F_{1,4,5,8}$	-0.01	$F_{2,4,5,8}$	0.01	$F_{3,4,5,8}$	0.03	$F_{4,4,5,8}$	0.01
$F_{1,5,5,8}$	0.01	$F_{2,5,5,8}$	0.01	$F_{3,5,5,8}$	0.00	$F_{4,5,5,8}$	-0.01
$F_{5,5,5,8}$	-0.03	$F_{1,1,6,8}$	-0.00	$F_{1,2,6,8}$	-0.02	$F_{2,2,6,8}$	-0.00
$F_{1,3,6,8}$	0.01	$F_{2,3,6,8}$	-0.01	$F_{3,3,6,8}$	0.01	$F_{1,4,6,8}$	0.01
$F_{2,4,6,8}$	0.02	$F_{3,4,6,8}$	-0.00	$F_{4,4,6,8}$	-0.00	$F_{1,5,6,8}$	0.02
$F_{2,5,6,8}$	0.01	$F_{3,5,6,8}$	-0.00	$F_{4,5,6,8}$	-0.03	$F_{5,5,6,8}$	-0.00
$F_{1,6,6,8}$	-0.01	$F_{2,6,6,8}$	0.02	$F_{3,6,6,8}$	-0.01	$F_{4,6,6,8}$	0.01
$F_{5,6,6,8}$	0.23	$F_{6,6,6,8}$	-0.07	$F_{1,1,7,8}$	0.00	$F_{1,2,7,8}$	-0.01
$F_{2,2,7,8}$	0.00	$F_{1,3,7,8}$	-0.00	$F_{2,3,7,8}$	-0.00	$F_{3,3,7,8}$	0.00
$F_{1,4,7,8}$	-0.00	$F_{2,4,7,8}$	0.01	$F_{3,4,7,8}$	0.00	$F_{4,4,7,8}$	0.01
$F_{1,5,7,8}$	0.01	$F_{2,5,7,8}$	-0.00	$F_{3,5,7,8}$	-0.01	$F_{4,5,7,8}$	-0.01
$F_{5,5,7,8}$	0.01	$F_{1,6,7,8}$	-0.00	$F_{2,6,7,8}$	0.00	$F_{3,6,7,8}$	0.00
$F_{4,6,7,8}$	-0.01	$F_{5,6,7,8}$	0.01	$F_{6,6,7,8}$	-0.01	$F_{1,7,7,8}$	0.00
$F_{2,7,7,8}$	-0.00	$F_{3,7,7,8}$	0.00	$F_{4,7,7,8}$	-0.01	$F_{5,7,7,8}$	0.02
$F_{6,7,7,8}$	0.01	$F_{7,7,7,8}$	-0.00	$F_{1,1,8,8}$	0.01	$F_{1,2,8,8}$	0.01
$F_{2,2,8,8}$	0.01	$F_{1,3,8,8}$	0.01	$F_{2,3,8,8}$	-0.00	$F_{3,3,8,8}$	-0.03
$F_{1,4,8,8}$	-0.01	$F_{2,4,8,8}$	-0.01	$F_{3,4,8,8}$	-0.02	$F_{4,4,8,8}$	0.02
$F_{1,5,8,8}$	-0.01	$F_{2,5,8,8}$	-0.00	$F_{3,5,8,8}$	0.00	$F_{4,5,8,8}$	0.01
$F_{5,5,8,8}$	0.01	$F_{1,6,8,8}$	0.00	$F_{2,6,8,8}$	-0.01	$F_{3,6,8,8}$	0.01
$F_{4,6,8,8}$	0.01	$F_{5,6,8,8}$	0.01	$F_{6,6,8,8}$	-0.27	$F_{1,7,8,8}$	-0.01
$F_{2,7,8,8}$	-0.00	$F_{3,7,8,8}$	0.01	$F_{4,7,8,8}$	0.01	$F_{5,7,8,8}$	-0.00
$F_{6,7,8,8}$	-0.00	$F_{7,7,8,8}$	-0.40	$F_{1,8,8,8}$	0.00	$F_{2,8,8,8}$	-0.01
$F_{3,8,8,8}$	-0.00	$F_{4,8,8,8}$	-0.01	$F_{5,8,8,8}$	-0.01	$F_{6,8,8,8}$	-0.01
$F_{7,8,8,8}$	0.03	$F_{8,8,8,8}$	1.60	$F_{1,1,1,9}$	0.00	$F_{1,1,2,9}$	-0.00
$F_{1,2,2,9}$	-0.03	$F_{2,2,2,9}$	0.01	$F_{1,1,3,9}$	0.01	$F_{1,2,3,9}$	0.00
$F_{2,2,3,9}$	0.00	$F_{1,3,3,9}$	-0.05	$F_{2,3,3,9}$	0.00	$F_{3,3,3,9}$	-0.04
$F_{1,1,4,9}$	0.00	$F_{1,2,4,9}$	0.01	$F_{2,2,4,9}$	0.04	$F_{1,3,4,9}$	-0.02
$F_{2,3,4,9}$	-0.00	$F_{3,3,4,9}$	0.03	$F_{1,4,4,9}$	-0.01	$F_{2,4,4,9}$	-0.00
$F_{3,4,4,9}$	0.04	$F_{4,4,4,9}$	0.01	$F_{1,1,5,9}$	0.01	$F_{1,2,5,9}$	0.03
$F_{2,2,5,9}$	-0.01	$F_{1,3,5,9}$	-0.00	$F_{2,3,5,9}$	0.01	$F_{3,3,5,9}$	0.00
$F_{1,4,5,9}$	-0.01	$F_{2,4,5,9}$	-0.03	$F_{3,4,5,9}$	0.00	$F_{4,4,5,9}$	0.02
$F_{1,5,5,9}$	-0.04	$F_{2,5,5,9}$	0.02	$F_{3,5,5,9}$	-0.00	$F_{4,5,5,9}$	0.03
$F_{5,5,5,9}$	-0.00	$F_{1,1,6,9}$	0.04	$F_{1,2,6,9}$	-0.01	$F_{2,2,6,9}$	-0.01
$F_{1,3,6,9}$	0.05	$F_{2,3,6,9}$	-0.00	$F_{3,3,6,9}$	0.03	$F_{1,4,6,9}$	-0.02
$F_{2,4,6,9}$	0.00	$F_{3,4,6,9}$	-0.04	$F_{4,4,6,9}$	0.26	$F_{1,5,6,9}$	0.01
$F_{2,5,6,9}$	0.02	$F_{3,5,6,9}$	-0.00	$F_{4,5,6,9}$	-0.00	$F_{5,5,6,9}$	0.21

Table S13: The CcCR Force Constants for HOSH (cont.)

$F_{1,6,6,9}$	-0.08	$F_{2,6,6,9}$	0.00	$F_{3,6,6,9}$	-0.06	$F_{4,6,6,9}$	0.16
$F_{5,6,6,9}$	-0.08	$F_{6,6,6,9}$	-1.74	$F_{1,1,7,9}$	-0.00	$F_{1,2,7,9}$	0.00
$F_{2,2,7,9}$	-0.01	$F_{1,3,7,9}$	0.01	$F_{2,3,7,9}$	-0.00	$F_{3,3,7,9}$	0.02
$F_{1,4,7,9}$	0.00	$F_{2,4,7,9}$	-0.01	$F_{3,4,7,9}$	-0.02	$F_{4,4,7,9}$	0.00
$F_{1,5,7,9}$	-0.00	$F_{2,5,7,9}$	0.01	$F_{3,5,7,9}$	0.00	$F_{4,5,7,9}$	-0.01
$F_{5,5,7,9}$	-0.01	$F_{1,6,7,9}$	-0.02	$F_{2,6,7,9}$	0.01	$F_{3,6,7,9}$	0.00
$F_{4,6,7,9}$	-0.25	$F_{5,6,7,9}$	-0.02	$F_{6,6,7,9}$	-0.08	$F_{1,7,7,9}$	0.00
$F_{2,7,7,9}$	0.01	$F_{3,7,7,9}$	0.01	$F_{4,7,7,9}$	-0.00	$F_{5,7,7,9}$	0.03
$F_{6,7,7,9}$	0.26	$F_{7,7,7,9}$	0.00	$F_{1,1,8,9}$	0.00	$F_{1,2,8,9}$	-0.00
$F_{2,2,8,9}$	0.01	$F_{1,3,8,9}$	-0.00	$F_{2,3,8,9}$	-0.01	$F_{3,3,8,9}$	-0.01
$F_{1,4,8,9}$	-0.01	$F_{2,4,8,9}$	-0.01	$F_{3,4,8,9}$	0.00	$F_{4,4,8,9}$	-0.01
$F_{1,5,8,9}$	0.00	$F_{2,5,8,9}$	-0.01	$F_{3,5,8,9}$	-0.01	$F_{4,5,8,9}$	0.00
$F_{5,5,8,9}$	-0.02	$F_{1,6,8,9}$	0.00	$F_{2,6,8,9}$	-0.02	$F_{3,6,8,9}$	0.01
$F_{4,6,8,9}$	-0.01	$F_{5,6,8,9}$	-0.22	$F_{6,6,8,9}$	0.09	$F_{1,7,8,9}$	0.00
$F_{2,7,8,9}$	-0.00	$F_{3,7,8,9}$	0.00	$F_{4,7,8,9}$	0.04	$F_{5,7,8,9}$	0.01
$F_{6,7,8,9}$	0.01	$F_{7,7,8,9}$	0.01	$F_{1,8,8,9}$	-0.01	$F_{2,8,8,9}$	0.01
$F_{3,8,8,9}$	0.02	$F_{4,8,8,9}$	0.01	$F_{5,8,8,9}$	0.02	$F_{6,8,8,9}$	0.24
$F_{7,8,8,9}$	-0.01	$F_{8,8,8,9}$	-0.47	$F_{1,1,9,9}$	-0.04	$F_{1,2,9,9}$	0.01
$F_{2,2,9,9}$	0.00	$F_{1,3,9,9}$	0.01	$F_{2,3,9,9}$	0.00	$F_{3,3,9,9}$	0.01
$F_{1,4,9,9}$	0.03	$F_{2,4,9,9}$	-0.00	$F_{3,4,9,9}$	0.01	$F_{4,4,9,9}$	-0.31
$F_{1,5,9,9}$	-0.01	$F_{2,5,9,9}$	-0.03	$F_{3,5,9,9}$	-0.00	$F_{4,5,9,9}$	-0.00
$F_{5,5,9,9}$	-0.21	$F_{1,6,9,9}$	0.04	$F_{2,6,9,9}$	0.00	$F_{3,6,9,9}$	0.03
$F_{4,6,9,9}$	-0.12	$F_{5,6,9,9}$	0.10	$F_{6,6,9,9}$	1.80	$F_{1,7,9,9}$	0.01
$F_{2,7,9,9}$	-0.01	$F_{3,7,9,9}$	-0.02	$F_{4,7,9,9}$	0.28	$F_{5,7,9,9}$	0.02
$F_{6,7,9,9}$	0.08	$F_{7,7,9,9}$	-0.27	$F_{1,8,9,9}$	-0.00	$F_{2,8,9,9}$	0.03
$F_{3,8,9,9}$	-0.00	$F_{4,8,9,9}$	0.01	$F_{5,8,9,9}$	0.23	$F_{6,8,9,9}$	-0.12
$F_{7,8,9,9}$	-0.02	$F_{8,8,9,9}$	-0.47	$F_{1,9,9,9}$	-0.04	$F_{2,9,9,9}$	0.00
$F_{3,9,9,9}$	-0.03	$F_{4,9,9,9}$	0.11	$F_{5,9,9,9}$	-0.09	$F_{6,9,9,9}$	-1.83
$F_{7,9,9,9}$	-0.07	$F_{8,9,9,9}$	0.30	$F_{9,9,9,9}$	1.84	$F_{1,1,1,10}$	0.00
$F_{1,1,2,10}$	0.00	$F_{1,2,2,10}$	0.00	$F_{2,2,2,10}$	-0.01	$F_{1,1,3,10}$	0.00
$F_{1,2,3,10}$	0.01	$F_{2,2,3,10}$	-0.00	$F_{1,3,3,10}$	-0.00	$F_{2,3,3,10}$	0.00
$F_{3,3,3,10}$	0.00	$F_{1,1,4,10}$	-0.00	$F_{1,2,4,10}$	0.00	$F_{2,2,4,10}$	-0.00
$F_{1,3,4,10}$	-0.00	$F_{2,3,4,10}$	-0.00	$F_{3,3,4,10}$	0.00	$F_{1,4,4,10}$	0.00
$F_{2,4,4,10}$	-0.00	$F_{3,4,4,10}$	0.00	$F_{4,4,4,10}$	-0.01	$F_{1,1,5,10}$	-0.00
$F_{1,2,5,10}$	-0.00	$F_{2,2,5,10}$	0.01	$F_{1,3,5,10}$	-0.01	$F_{2,3,5,10}$	-0.00
$F_{3,3,5,10}$	-0.00	$F_{1,4,5,10}$	-0.00	$F_{2,4,5,10}$	0.00	$F_{3,4,5,10}$	0.00
$F_{4,4,5,10}$	0.01	$F_{1,5,5,10}$	0.00	$F_{2,5,5,10}$	-0.01	$F_{3,5,5,10}$	0.00
$F_{4,5,5,10}$	-0.00	$F_{5,5,5,10}$	0.01	$F_{1,1,6,10}$	0.00	$F_{1,2,6,10}$	-0.00
$F_{2,2,6,10}$	-0.00	$F_{1,3,6,10}$	0.00	$F_{2,3,6,10}$	-0.00	$F_{3,3,6,10}$	-0.00
$F_{1,4,6,10}$	-0.00	$F_{2,4,6,10}$	-0.00	$F_{3,4,6,10}$	-0.00	$F_{4,4,6,10}$	0.00
$F_{1,5,6,10}$	0.00	$F_{2,5,6,10}$	0.01	$F_{3,5,6,10}$	0.00	$F_{4,5,6,10}$	-0.01
$F_{5,5,6,10}$	-0.01	$F_{1,6,6,10}$	0.00	$F_{2,6,6,10}$	0.01	$F_{3,6,6,10}$	0.01
$F_{4,6,6,10}$	0.01	$F_{5,6,6,10}$	-0.01	$F_{6,6,6,10}$	-0.01	$F_{1,1,7,10}$	0.00
$F_{1,2,7,10}$	-0.00	$F_{2,2,7,10}$	0.00	$F_{1,3,7,10}$	-0.00	$F_{2,3,7,10}$	-0.00
$F_{3,3,7,10}$	-0.00	$F_{1,4,7,10}$	-0.00	$F_{2,4,7,10}$	0.01	$F_{3,4,7,10}$	0.00
$F_{4,4,7,10}$	0.00	$F_{1,5,7,10}$	0.00	$F_{2,5,7,10}$	-0.00	$F_{3,5,7,10}$	0.00
$F_{4,5,7,10}$	-0.01	$F_{5,5,7,10}$	0.01	$F_{1,6,7,10}$	0.00	$F_{2,6,7,10}$	0.00
$F_{3,6,7,10}$	0.00	$F_{4,6,7,10}$	0.00	$F_{5,6,7,10}$	0.00	$F_{6,6,7,10}$	-0.02
$F_{1,7,7,10}$	0.01	$F_{2,7,7,10}$	-0.01	$F_{3,7,7,10}$	0.00	$F_{4,7,7,10}$	0.01
$F_{5,7,7,10}$	0.01	$F_{6,7,7,10}$	-0.01	$F_{7,7,7,10}$	-0.14	$F_{1,1,8,10}$	0.00
$F_{1,2,8,10}$	0.00	$F_{2,2,8,10}$	-0.00	$F_{1,3,8,10}$	0.00	$F_{2,3,8,10}$	0.00
$F_{3,3,8,10}$	0.00	$F_{1,4,8,10}$	0.00	$F_{2,4,8,10}$	-0.00	$F_{3,4,8,10}$	-0.00
$F_{4,4,8,10}$	-0.00	$F_{1,5,8,10}$	-0.00	$F_{2,5,8,10}$	0.00	$F_{3,5,8,10}$	-0.00
$F_{4,5,8,10}$	0.01	$F_{5,5,8,10}$	-0.00	$F_{1,6,8,10}$	-0.00	$F_{2,6,8,10}$	-0.00
$F_{3,6,8,10}$	-0.00	$F_{4,6,8,10}$	0.01	$F_{5,6,8,10}$	0.01	$F_{6,6,8,10}$	0.01
$F_{1,7,8,10}$	-0.00	$F_{2,7,8,10}$	0.00	$F_{3,7,8,10}$	-0.00	$F_{4,7,8,10}$	0.01
$F_{5,7,8,10}$	-0.02	$F_{6,7,8,10}$	0.00	$F_{7,7,8,10}$	0.01	$F_{1,8,8,10}$	0.00
$F_{2,8,8,10}$	0.00	$F_{3,8,8,10}$	-0.00	$F_{4,8,8,10}$	-0.02	$F_{5,8,8,10}$	0.00
$F_{6,8,8,10}$	-0.01	$F_{7,8,8,10}$	0.40	$F_{8,8,8,10}$	-0.03	$F_{1,1,9,10}$	-0.00
$F_{1,2,9,10}$	-0.00	$F_{2,2,9,10}$	0.00	$F_{1,3,9,10}$	0.00	$F_{2,3,9,10}$	0.00
$F_{3,3,9,10}$	0.00	$F_{1,4,9,10}$	0.00	$F_{2,4,9,10}$	0.01	$F_{3,4,9,10}$	-0.00
$F_{4,4,9,10}$	-0.00	$F_{1,5,9,10}$	0.00	$F_{2,5,9,10}$	-0.01	$F_{3,5,9,10}$	-0.00
$F_{4,5,9,10}$	0.00	$F_{5,5,9,10}$	0.01	$F_{1,6,9,10}$	-0.00	$F_{2,6,9,10}$	-0.01
$F_{3,6,9,10}$	-0.00	$F_{4,6,9,10}$	0.00	$F_{5,6,9,10}$	0.01	$F_{6,6,9,10}$	0.00
$F_{1,7,9,10}$	-0.00	$F_{2,7,9,10}$	-0.00	$F_{3,7,9,10}$	-0.00	$F_{4,7,9,10}$	-0.00
$F_{5,7,9,10}$	-0.01	$F_{6,7,9,10}$	0.00	$F_{7,7,9,10}$	-0.00	$F_{1,8,9,10}$	0.00
$F_{2,8,9,10}$	0.01	$F_{3,8,9,10}$	0.00	$F_{4,8,9,10}$	-0.02	$F_{5,8,9,10}$	-0.01
$F_{6,8,9,10}$	-0.01	$F_{7,8,9,10}$	-0.05	$F_{8,8,9,10}$	0.01	$F_{1,9,9,10}$	0.00
$F_{2,9,9,10}$	0.01	$F_{3,9,9,10}$	-0.00	$F_{4,9,9,10}$	-0.01	$F_{5,9,9,10}$	-0.01
$F_{6,9,9,10}$	-0.00	$F_{7,9,9,10}$	-0.01	$F_{8,9,9,10}$	0.01	$F_{9,9,9,10}$	0.00
$F_{1,1,10,10}$	-0.00	$F_{1,2,10,10}$	0.00	$F_{2,2,10,10}$	-0.00	$F_{1,3,10,10}$	0.00
$F_{2,3,10,10}$	-0.00	$F_{3,3,10,10}$	0.00	$F_{1,4,10,10}$	0.00	$F_{2,4,10,10}$	-0.00
$F_{3,4,10,10}$	-0.00	$F_{4,4,10,10}$	0.00	$F_{1,5,10,10}$	-0.00	$F_{2,5,10,10}$	0.00
$F_{3,5,10,10}$	-0.00	$F_{4,5,10,10}$	0.00	$F_{5,5,10,10}$	-0.01	$F_{1,6,10,10}$	-0.00

Table S14: The CcCR Force Constants for HOSH (cont.)

$F_{2,6,10,10}$	-0.00	$F_{3,6,10,10}$	-0.00	$F_{4,6,10,10}$	0.00	$F_{5,6,10,10}$	0.01
$F_{6,6,10,10}$	-0.00	$F_{1,7,10,10}$	-0.00	$F_{2,7,10,10}$	0.00	$F_{3,7,10,10}$	-0.00
$F_{4,7,10,10}$	-0.01	$F_{5,7,10,10}$	-0.00	$F_{6,7,10,10}$	0.00	$F_{7,7,10,10}$	0.13
$F_{1,8,10,10}$	0.00	$F_{2,8,10,10}$	-0.00	$F_{3,8,10,10}$	-0.00	$F_{4,8,10,10}$	-0.00
$F_{5,8,10,10}$	0.01	$F_{6,8,10,10}$	-0.01	$F_{7,8,10,10}$	-0.01	$F_{8,8,10,10}$	-0.38
$F_{1,9,10,10}$	-0.00	$F_{2,9,10,10}$	0.00	$F_{3,9,10,10}$	0.00	$F_{4,9,10,10}$	0.00
$F_{5,9,10,10}$	0.00	$F_{6,9,10,10}$	-0.00	$F_{7,9,10,10}$	0.00	$F_{8,9,10,10}$	0.07
$F_{9,9,10,10}$	0.02	$F_{1,10,10,10}$	0.00	$F_{2,10,10,10}$	-0.00	$F_{3,10,10,10}$	0.00
$F_{4,10,10,10}$	0.00	$F_{5,10,10,10}$	0.00	$F_{6,10,10,10}$	-0.00	$F_{7,10,10,10}$	-0.12
$F_{8,10,10,10}$	0.01	$F_{9,10,10,10}$	-0.00	$F_{10,10,10,10}$	0.12	$F_{1,1,1,11}$	-0.00
$F_{1,1,2,11}$	-0.00	$F_{1,2,2,11}$	-0.00	$F_{2,2,2,11}$	0.00	$F_{1,1,3,11}$	0.00
$F_{1,2,3,11}$	-0.00	$F_{2,2,3,11}$	0.00	$F_{1,3,3,11}$	0.00	$F_{2,3,3,11}$	-0.00
$F_{3,3,3,11}$	0.00	$F_{1,1,4,11}$	0.00	$F_{1,2,4,11}$	0.00	$F_{2,2,4,11}$	0.00
$F_{1,3,4,11}$	-0.00	$F_{2,3,4,11}$	0.00	$F_{3,3,4,11}$	0.00	$F_{1,4,4,11}$	-0.00
$F_{2,4,4,11}$	-0.00	$F_{3,4,4,11}$	0.00	$F_{4,4,4,11}$	0.01	$F_{1,1,5,11}$	0.00
$F_{1,2,5,11}$	0.01	$F_{2,2,5,11}$	-0.00	$F_{1,3,5,11}$	0.00	$F_{2,3,5,11}$	-0.00
$F_{3,3,5,11}$	0.00	$F_{1,4,5,11}$	-0.00	$F_{2,4,5,11}$	-0.01	$F_{3,4,5,11}$	-0.00
$F_{4,4,5,11}$	-0.00	$F_{1,5,5,11}$	-0.01	$F_{2,5,5,11}$	0.00	$F_{3,5,5,11}$	0.00
$F_{4,5,5,11}$	0.01	$F_{5,5,5,11}$	-0.01	$F_{1,1,6,11}$	0.00	$F_{1,2,6,11}$	0.00
$F_{2,2,6,11}$	-0.00	$F_{1,3,6,11}$	-0.01	$F_{2,3,6,11}$	0.00	$F_{3,3,6,11}$	-0.01
$F_{1,4,6,11}$	-0.00	$F_{2,4,6,11}$	0.00	$F_{3,4,6,11}$	0.00	$F_{4,4,6,11}$	0.00
$F_{1,5,6,11}$	0.00	$F_{2,5,6,11}$	0.00	$F_{3,5,6,11}$	-0.00	$F_{4,5,6,11}$	-0.00
$F_{5,5,6,11}$	-0.00	$F_{1,6,6,11}$	0.01	$F_{2,6,6,11}$	-0.01	$F_{3,6,6,11}$	0.01
$F_{4,6,6,11}$	-0.01	$F_{5,6,6,11}$	0.00	$F_{6,6,6,11}$	0.03	$F_{1,1,7,11}$	-0.00
$F_{1,2,7,11}$	0.00	$F_{2,2,7,11}$	-0.00	$F_{1,3,7,11}$	0.00	$F_{2,3,7,11}$	-0.00
$F_{3,3,7,11}$	-0.00	$F_{1,4,7,11}$	0.00	$F_{2,4,7,11}$	-0.00	$F_{3,4,7,11}$	-0.00
$F_{4,4,7,11}$	-0.00	$F_{1,5,7,11}$	-0.00	$F_{2,5,7,11}$	0.00	$F_{3,5,7,11}$	0.00
$F_{4,5,7,11}$	0.01	$F_{5,5,7,11}$	-0.00	$F_{1,6,7,11}$	0.00	$F_{2,6,7,11}$	-0.00
$F_{3,6,7,11}$	-0.00	$F_{4,6,7,11}$	0.00	$F_{5,6,7,11}$	0.00	$F_{6,6,7,11}$	0.00
$F_{1,7,7,11}$	-0.00	$F_{2,7,7,11}$	0.00	$F_{3,7,7,11}$	0.00	$F_{4,7,7,11}$	0.00
$F_{5,7,7,11}$	-0.02	$F_{6,7,7,11}$	0.01	$F_{7,7,7,11}$	0.01	$F_{1,1,8,11}$	-0.00
$F_{1,2,8,11}$	-0.00	$F_{2,2,8,11}$	0.00	$F_{1,3,8,11}$	0.00	$F_{2,3,8,11}$	0.00
$F_{3,3,8,11}$	0.00	$F_{1,4,8,11}$	0.00	$F_{2,4,8,11}$	0.00	$F_{3,4,8,11}$	0.00
$F_{4,4,8,11}$	-0.01	$F_{1,5,8,11}$	0.00	$F_{2,5,8,11}$	-0.00	$F_{3,5,8,11}$	-0.00
$F_{4,5,8,11}$	-0.01	$F_{5,5,8,11}$	0.01	$F_{1,6,8,11}$	-0.00	$F_{2,6,8,11}$	0.00
$F_{3,6,8,11}$	0.00	$F_{4,6,8,11}$	0.00	$F_{5,6,8,11}$	-0.01	$F_{6,6,8,11}$	0.02
$F_{1,7,8,11}$	0.00	$F_{2,7,8,11}$	0.00	$F_{3,7,8,11}$	-0.00	$F_{4,7,8,11}$	-0.01
$F_{5,7,8,11}$	0.00	$F_{6,7,8,11}$	-0.00	$F_{7,7,8,11}$	0.39	$F_{1,8,8,11}$	-0.00
$F_{2,8,8,11}$	0.00	$F_{3,8,8,11}$	0.00	$F_{4,8,8,11}$	0.00	$F_{5,8,8,11}$	-0.01
$F_{6,8,8,11}$	0.01	$F_{7,8,8,11}$	-0.03	$F_{8,8,8,11}$	-1.58	$F_{1,1,9,11}$	-0.01
$F_{1,2,9,11}$	-0.00	$F_{2,2,9,11}$	-0.00	$F_{1,3,9,11}$	0.00	$F_{2,3,9,11}$	-0.00
$F_{3,3,9,11}$	0.00	$F_{1,4,9,11}$	0.01	$F_{2,4,9,11}$	0.00	$F_{3,4,9,11}$	-0.00
$F_{4,4,9,11}$	-0.01	$F_{1,5,9,11}$	0.00	$F_{2,5,9,11}$	0.00	$F_{3,5,9,11}$	-0.00
$F_{4,5,9,11}$	0.00	$F_{5,5,9,11}$	-0.00	$F_{1,6,9,11}$	-0.00	$F_{2,6,9,11}$	0.00
$F_{3,6,9,11}$	-0.00	$F_{4,6,9,11}$	0.00	$F_{5,6,9,11}$	-0.01	$F_{6,6,9,11}$	-0.01
$F_{1,7,9,11}$	-0.00	$F_{2,7,9,11}$	0.00	$F_{3,7,9,11}$	-0.00	$F_{4,7,9,11}$	-0.01
$F_{5,7,9,11}$	-0.00	$F_{6,7,9,11}$	-0.00	$F_{7,7,9,11}$	-0.05	$F_{1,8,9,11}$	0.00
$F_{2,8,9,11}$	-0.00	$F_{3,8,9,11}$	-0.00	$F_{4,8,9,11}$	-0.00	$F_{5,8,9,11}$	0.01
$F_{6,8,9,11}$	-0.00	$F_{7,8,9,11}$	0.01	$F_{8,8,9,11}$	0.45	$F_{1,9,9,11}$	0.00
$F_{2,9,9,11}$	-0.00	$F_{3,9,9,11}$	0.00	$F_{4,9,9,11}$	-0.00	$F_{5,9,9,11}$	0.01
$F_{6,9,9,11}$	0.02	$F_{7,9,9,11}$	0.00	$F_{8,9,9,11}$	0.21	$F_{9,9,9,11}$	-0.21
$F_{1,1,10,11}$	-0.00	$F_{1,2,10,11}$	-0.00	$F_{2,2,10,11}$	0.00	$F_{1,3,10,11}$	-0.00
$F_{2,3,10,11}$	0.00	$F_{3,3,10,11}$	-0.00	$F_{1,4,10,11}$	-0.00	$F_{2,4,10,11}$	0.00
$F_{3,4,10,11}$	0.00	$F_{4,4,10,11}$	0.00	$F_{1,5,10,11}$	0.00	$F_{2,5,10,11}$	-0.00
$F_{3,5,10,11}$	-0.00	$F_{4,5,10,11}$	-0.01	$F_{5,5,10,11}$	-0.00	$F_{1,6,10,11}$	0.00
$F_{2,6,10,11}$	0.00	$F_{3,6,10,11}$	0.00	$F_{4,6,10,11}$	0.00	$F_{5,6,10,11}$	-0.00
$F_{6,6,10,11}$	-0.00	$F_{1,7,10,11}$	0.00	$F_{2,7,10,11}$	-0.00	$F_{3,7,10,11}$	-0.00
$F_{4,7,10,11}$	-0.00	$F_{5,7,10,11}$	0.01	$F_{6,7,10,11}$	-0.01	$F_{7,7,10,11}$	-0.01
$F_{1,8,10,11}$	-0.00	$F_{2,8,10,11}$	-0.00	$F_{3,8,10,11}$	0.00	$F_{4,8,10,11}$	0.01
$F_{5,8,10,11}$	0.00	$F_{6,8,10,11}$	0.00	$F_{7,8,10,11}$	-0.38	$F_{8,8,10,11}$	0.03
$F_{1,9,10,11}$	-0.00	$F_{2,9,10,11}$	-0.00	$F_{3,9,10,11}$	0.00	$F_{4,9,10,11}$	0.01
$F_{5,9,10,11}$	0.00	$F_{6,9,10,11}$	0.00	$F_{7,9,10,11}$	0.07	$F_{8,9,10,11}$	-0.01
$F_{9,9,10,11}$	-0.00	$F_{1,10,10,11}$	-0.00	$F_{2,10,10,11}$	0.00	$F_{3,10,10,11}$	0.00
$F_{4,10,10,11}$	0.00	$F_{5,10,10,11}$	-0.00	$F_{6,10,10,11}$	0.01	$F_{7,10,10,11}$	0.01
$F_{8,10,10,11}$	0.37	$F_{9,10,10,11}$	-0.08	$F_{10,10,10,11}$	-0.01	$F_{1,1,11,11}$	0.00
$F_{1,2,11,11}$	0.00	$F_{2,2,11,11}$	-0.00	$F_{1,3,11,11}$	-0.00	$F_{2,3,11,11}$	0.00
$F_{3,3,11,11}$	-0.00	$F_{1,4,11,11}$	-0.00	$F_{2,4,11,11}$	-0.00	$F_{3,4,11,11}$	-0.00
$F_{4,4,11,11}$	0.01	$F_{1,5,11,11}$	-0.00	$F_{2,5,11,11}$	0.00	$F_{3,5,11,11}$	0.00
$F_{4,5,11,11}$	0.00	$F_{5,5,11,11}$	-0.00	$F_{1,6,11,11}$	0.00	$F_{2,6,11,11}$	-0.00
$F_{3,6,11,11}$	-0.00	$F_{4,6,11,11}$	-0.00	$F_{5,6,11,11}$	0.01	$F_{6,6,11,11}$	-0.01
$F_{1,7,11,11}$	-0.00	$F_{2,7,11,11}$	-0.00	$F_{3,7,11,11}$	0.00	$F_{4,7,11,11}$	0.00
$F_{5,7,11,11}$	0.00	$F_{6,7,11,11}$	0.00	$F_{7,7,11,11}$	-0.37	$F_{1,8,11,11}$	0.00
$F_{2,8,11,11}$	-0.00	$F_{3,8,11,11}$	-0.00	$F_{4,8,11,11}$	-0.00	$F_{5,8,11,11}$	0.00
$F_{6,8,11,11}$	-0.00	$F_{7,8,11,11}$	0.03	$F_{8,8,11,11}$	1.59	$F_{1,9,11,11}$	-0.00

Table S15: The CcCR Force Constants for HOSH (cont.)

$F_{2,9,11,11}$	0.00	$F_{3,9,11,11}$	0.00	$F_{4,9,11,11}$	0.00	$F_{5,9,11,11}$	-0.02
$F_{6,9,11,11}$	0.01	$F_{7,9,11,11}$	-0.01	$F_{8,9,11,11}$	-0.46	$F_{9,9,11,11}$	-0.23
$F_{1,10,11,11}$	0.00	$F_{2,10,11,11}$	0.00	$F_{3,10,11,11}$	-0.00	$F_{4,10,11,11}$	-0.01
$F_{5,10,11,11}$	-0.00	$F_{6,10,11,11}$	-0.00	$F_{7,10,11,11}$	0.37	$F_{8,10,11,11}$	-0.03
$F_{9,10,11,11}$	0.01	$F_{10,10,11,11}$	-0.36	$F_{1,11,11,11}$	-0.00	$F_{2,11,11,11}$	0.00
$F_{3,11,11,11}$	0.00	$F_{4,11,11,11}$	0.00	$F_{5,11,11,11}$	-0.00	$F_{6,11,11,11}$	-0.00
$F_{7,11,11,11}$	-0.03	$F_{8,11,11,11}$	-1.59	$F_{9,11,11,11}$	0.48	$F_{10,11,11,11}$	0.03
$F_{11,11,11,11}$	1.59	$F_{1,1,1,12}$	0.00	$F_{1,1,2,12}$	0.00	$F_{1,2,2,12}$	0.00
$F_{2,2,2,12}$	-0.00	$F_{1,1,3,12}$	-0.00	$F_{1,2,3,12}$	-0.00	$F_{2,2,3,12}$	-0.00
$F_{1,3,3,12}$	-0.00	$F_{2,3,3,12}$	-0.00	$F_{3,3,3,12}$	0.00	$F_{1,1,4,12}$	-0.00
$F_{1,2,4,12}$	-0.00	$F_{2,2,4,12}$	-0.00	$F_{1,3,4,12}$	0.00	$F_{2,3,4,12}$	0.00
$F_{3,3,4,12}$	0.00	$F_{1,4,4,12}$	0.00	$F_{2,4,4,12}$	0.00	$F_{3,4,4,12}$	-0.00
$F_{4,4,4,12}$	-0.00	$F_{1,1,5,12}$	-0.00	$F_{1,2,5,12}$	0.00	$F_{2,2,5,12}$	0.00
$F_{1,3,5,12}$	0.00	$F_{2,3,5,12}$	0.00	$F_{3,3,5,12}$	-0.00	$F_{1,4,5,12}$	0.00
$F_{2,4,5,12}$	0.00	$F_{3,4,5,12}$	-0.01	$F_{4,4,5,12}$	-0.02	$F_{1,5,5,12}$	-0.00
$F_{2,5,5,12}$	-0.01	$F_{3,5,5,12}$	-0.00	$F_{4,5,5,12}$	-0.00	$F_{5,5,5,12}$	-0.02
$F_{1,1,6,12}$	0.00	$F_{1,2,6,12}$	0.00	$F_{2,2,6,12}$	0.00	$F_{1,3,6,12}$	0.00
$F_{2,3,6,12}$	-0.00	$F_{3,3,6,12}$	-0.00	$F_{1,4,6,12}$	-0.00	$F_{2,4,6,12}$	-0.00
$F_{3,4,6,12}$	-0.00	$F_{4,4,6,12}$	0.01	$F_{1,5,6,12}$	-0.00	$F_{2,5,6,12}$	-0.01
$F_{3,5,6,12}$	0.00	$F_{4,5,6,12}$	0.00	$F_{5,5,6,12}$	0.01	$F_{1,6,6,12}$	-0.00
$F_{2,6,6,12}$	0.00	$F_{3,6,6,12}$	0.00	$F_{4,6,6,12}$	0.01	$F_{5,6,6,12}$	0.03
$F_{6,6,6,12}$	-0.03	$F_{1,1,7,12}$	-0.00	$F_{1,2,7,12}$	-0.00	$F_{2,2,7,12}$	0.00
$F_{1,3,7,12}$	0.00	$F_{2,3,7,12}$	-0.00	$F_{3,3,7,12}$	-0.00	$F_{1,4,7,12}$	0.00
$F_{2,4,7,12}$	0.00	$F_{3,4,7,12}$	-0.00	$F_{4,4,7,12}$	-0.00	$F_{1,5,7,12}$	-0.00
$F_{2,5,7,12}$	-0.00	$F_{3,5,7,12}$	0.00	$F_{4,5,7,12}$	0.01	$F_{5,5,7,12}$	0.01
$F_{1,6,7,12}$	-0.00	$F_{2,6,7,12}$	-0.00	$F_{3,6,7,12}$	0.00	$F_{4,6,7,12}$	0.00
$F_{5,6,7,12}$	0.01	$F_{6,6,7,12}$	-0.00	$F_{1,7,7,12}$	-0.00	$F_{2,7,7,12}$	-0.00
$F_{3,7,7,12}$	0.00	$F_{4,7,7,12}$	0.00	$F_{5,7,7,12}$	-0.02	$F_{6,7,7,12}$	-0.01
$F_{7,7,7,12}$	-0.00	$F_{1,1,8,12}$	-0.00	$F_{1,2,8,12}$	-0.00	$F_{2,2,8,12}$	-0.00
$F_{1,3,8,12}$	-0.00	$F_{2,3,8,12}$	0.00	$F_{3,3,8,12}$	0.00	$F_{1,4,8,12}$	0.00
$F_{2,4,8,12}$	0.00	$F_{3,4,8,12}$	0.00	$F_{4,4,8,12}$	0.01	$F_{1,5,8,12}$	0.00
$F_{2,5,8,12}$	0.00	$F_{3,5,8,12}$	-0.00	$F_{4,5,8,12}$	-0.00	$F_{5,5,8,12}$	0.02
$F_{1,6,8,12}$	-0.00	$F_{2,6,8,12}$	0.00	$F_{3,6,8,12}$	-0.01	$F_{4,6,8,12}$	-0.00
$F_{5,6,8,12}$	-0.01	$F_{6,6,8,12}$	-0.01	$F_{1,7,8,12}$	0.00	$F_{2,7,8,12}$	0.00
$F_{3,7,8,12}$	-0.00	$F_{4,7,8,12}$	-0.02	$F_{5,7,8,12}$	-0.00	$F_{6,7,8,12}$	0.00
$F_{7,7,8,12}$	-0.03	$F_{1,8,8,12}$	-0.00	$F_{2,8,8,12}$	0.00	$F_{3,8,8,12}$	0.00
$F_{4,8,8,12}$	0.00	$F_{5,8,8,12}$	-0.02	$F_{6,8,8,12}$	0.03	$F_{7,8,8,12}$	0.00
$F_{8,8,8,12}$	0.49	$F_{1,1,9,12}$	-0.00	$F_{1,2,9,12}$	-0.00	$F_{2,2,9,12}$	-0.00
$F_{1,3,9,12}$	-0.00	$F_{2,3,9,12}$	0.00	$F_{3,3,9,12}$	0.00	$F_{1,4,9,12}$	0.00
$F_{2,4,9,12}$	-0.00	$F_{3,4,9,12}$	0.00	$F_{4,4,9,12}$	0.01	$F_{1,5,9,12}$	0.00
$F_{2,5,9,12}$	0.00	$F_{3,5,9,12}$	0.00	$F_{4,5,9,12}$	0.00	$F_{5,5,9,12}$	0.00
$F_{1,6,9,12}$	0.00	$F_{2,6,9,12}$	0.00	$F_{3,6,9,12}$	-0.00	$F_{4,6,9,12}$	-0.00
$F_{5,6,9,12}$	-0.02	$F_{6,6,9,12}$	0.00	$F_{1,7,9,12}$	0.00	$F_{2,7,9,12}$	0.00
$F_{3,7,9,12}$	-0.00	$F_{4,7,9,12}$	-0.01	$F_{5,7,9,12}$	-0.01	$F_{6,7,9,12}$	-0.00
$F_{7,7,9,12}$	-0.00	$F_{1,8,9,12}$	0.00	$F_{2,8,9,12}$	-0.00	$F_{3,8,9,12}$	0.00
$F_{4,8,9,12}$	-0.00	$F_{5,8,9,12}$	-0.00	$F_{6,8,9,12}$	0.02	$F_{7,8,9,12}$	0.01
$F_{8,8,9,12}$	0.22	$F_{1,9,9,12}$	-0.00	$F_{2,9,9,12}$	-0.00	$F_{3,9,9,12}$	-0.01
$F_{4,9,9,12}$	-0.00	$F_{5,9,9,12}$	-0.01	$F_{6,9,9,12}$	-0.00	$F_{7,9,9,12}$	0.00
$F_{8,9,9,12}$	-0.18	$F_{9,9,9,12}$	0.02	$F_{1,1,10,12}$	0.00	$F_{1,2,10,12}$	0.00
$F_{2,2,10,12}$	0.00	$F_{1,3,10,12}$	-0.00	$F_{2,3,10,12}$	-0.00	$F_{3,3,10,12}$	0.00
$F_{1,4,10,12}$	-0.00	$F_{2,4,10,12}$	-0.00	$F_{3,4,10,12}$	0.00	$F_{4,4,10,12}$	0.00
$F_{1,5,10,12}$	-0.00	$F_{2,5,10,12}$	0.00	$F_{3,5,10,12}$	0.00	$F_{4,5,10,12}$	0.00
$F_{5,5,10,12}$	-0.00	$F_{1,6,10,12}$	0.00	$F_{2,6,10,12}$	0.00	$F_{3,6,10,12}$	-0.00
$F_{4,6,10,12}$	-0.01	$F_{5,6,10,12}$	-0.01	$F_{6,6,10,12}$	-0.00	$F_{1,7,10,12}$	0.00
$F_{2,7,10,12}$	0.00	$F_{3,7,10,12}$	0.00	$F_{4,7,10,12}$	-0.00	$F_{5,7,10,12}$	0.01
$F_{6,7,10,12}$	0.01	$F_{7,7,10,12}$	0.00	$F_{1,8,10,12}$	-0.00	$F_{2,8,10,12}$	-0.00
$F_{3,8,10,12}$	0.00	$F_{4,8,10,12}$	0.01	$F_{5,8,10,12}$	0.00	$F_{6,8,10,12}$	0.00
$F_{7,8,10,12}$	0.05	$F_{8,8,10,12}$	-0.00	$F_{1,9,10,12}$	-0.00	$F_{2,9,10,12}$	-0.00
$F_{3,9,10,12}$	0.00	$F_{4,9,10,12}$	0.01	$F_{5,9,10,12}$	0.00	$F_{6,9,10,12}$	0.00
$F_{7,9,10,12}$	0.01	$F_{8,9,10,12}$	-0.00	$F_{9,9,10,12}$	-0.00	$F_{1,10,10,12}$	-0.00
$F_{2,10,10,12}$	-0.00	$F_{3,10,10,12}$	-0.00	$F_{4,10,10,12}$	-0.00	$F_{5,10,10,12}$	-0.01
$F_{6,10,10,12}$	0.00	$F_{7,10,10,12}$	-0.00	$F_{8,10,10,12}$	-0.06	$F_{9,10,10,12}$	-0.02
$F_{10,10,10,12}$	0.00	$F_{1,1,11,12}$	0.00	$F_{1,2,11,12}$	0.00	$F_{2,2,11,12}$	0.00
$F_{1,3,11,12}$	-0.00	$F_{2,3,11,12}$	-0.00	$F_{3,3,11,12}$	-0.00	$F_{1,4,11,12}$	-0.00
$F_{2,4,11,12}$	-0.00	$F_{3,4,11,12}$	-0.00	$F_{4,4,11,12}$	0.00	$F_{1,5,11,12}$	-0.00
$F_{2,5,11,12}$	-0.00	$F_{3,5,11,12}$	0.00	$F_{4,5,11,12}$	0.00	$F_{5,5,11,12}$	-0.00
$F_{1,6,11,12}$	0.00	$F_{2,6,11,12}$	0.00	$F_{3,6,11,12}$	0.00	$F_{4,6,11,12}$	0.00
$F_{5,6,11,12}$	0.01	$F_{6,6,11,12}$	-0.03	$F_{1,7,11,12}$	-0.00	$F_{2,7,11,12}$	-0.00
$F_{3,7,11,12}$	0.00	$F_{4,7,11,12}$	0.01	$F_{5,7,11,12}$	0.00	$F_{6,7,11,12}$	-0.00
$F_{7,7,11,12}$	0.05	$F_{1,8,11,12}$	-0.00	$F_{2,8,11,12}$	-0.00	$F_{3,8,11,12}$	-0.00
$F_{4,8,11,12}$	0.00	$F_{5,8,11,12}$	-0.00	$F_{6,8,11,12}$	-0.02	$F_{7,8,11,12}$	-0.01
$F_{8,8,11,12}$	-0.47	$F_{1,9,11,12}$	-0.00	$F_{2,9,11,12}$	-0.00	$F_{3,9,11,12}$	-0.00
$F_{4,9,11,12}$	0.00	$F_{5,9,11,12}$	-0.00	$F_{6,9,11,12}$	-0.00	$F_{7,9,11,12}$	-0.00
$F_{8,9,11,12}$	-0.21	$F_{9,9,11,12}$	0.19	$F_{1,10,11,12}$	0.00	$F_{2,10,11,12}$	0.00

Table S16: The CcCR Force Constants for HOSH (cont.)

$F_{3,10,11,12}$	-0.00	$F_{4,10,11,12}$	-0.01	$F_{5,10,11,12}$	-0.00	$F_{6,10,11,12}$	0.00
$F_{7,10,11,12}$	-0.06	$F_{8,10,11,12}$	0.01	$F_{9,10,11,12}$	0.00	$F_{10,10,11,12}$	0.07
$F_{1,11,11,12}$	-0.00	$F_{2,11,11,12}$	0.00	$F_{3,11,11,12}$	0.00	$F_{4,11,11,12}$	0.00
$F_{5,11,11,12}$	0.00	$F_{6,11,11,12}$	0.01	$F_{7,11,11,12}$	0.01	$F_{8,11,11,12}$	0.47
$F_{9,11,11,12}$	0.22	$F_{10,11,11,12}$	-0.01	$F_{11,11,11,12}$	-0.47	$F_{1,1,12,12}$	-0.00
$F_{1,2,12,12}$	0.00	$F_{2,2,12,12}$	-0.00	$F_{1,3,12,12}$	0.00	$F_{2,3,12,12}$	0.00
$F_{3,3,12,12}$	-0.00	$F_{1,4,12,12}$	0.00	$F_{2,4,12,12}$	0.00	$F_{3,4,12,12}$	-0.00
$F_{4,4,12,12}$	-0.02	$F_{1,5,12,12}$	-0.00	$F_{2,5,12,12}$	0.00	$F_{3,5,12,12}$	-0.00
$F_{4,5,12,12}$	0.00	$F_{5,5,12,12}$	-0.01	$F_{1,6,12,12}$	-0.00	$F_{2,6,12,12}$	-0.00
$F_{3,6,12,12}$	-0.00	$F_{4,6,12,12}$	-0.00	$F_{5,6,12,12}$	-0.02	$F_{6,6,12,12}$	0.02
$F_{1,7,12,12}$	-0.00	$F_{2,7,12,12}$	-0.00	$F_{3,7,12,12}$	0.00	$F_{4,7,12,12}$	0.01
$F_{5,7,12,12}$	0.00	$F_{6,7,12,12}$	0.00	$F_{7,7,12,12}$	0.01	$F_{1,8,12,12}$	0.00
$F_{2,8,12,12}$	-0.00	$F_{3,8,12,12}$	-0.00	$F_{4,8,12,12}$	-0.00	$F_{5,8,12,12}$	0.02
$F_{6,8,12,12}$	-0.01	$F_{7,8,12,12}$	-0.00	$F_{8,8,12,12}$	-0.25	$F_{1,9,12,12}$	0.00
$F_{2,9,12,12}$	0.00	$F_{3,9,12,12}$	0.00	$F_{4,9,12,12}$	0.00	$F_{5,9,12,12}$	0.02
$F_{6,9,12,12}$	0.00	$F_{7,9,12,12}$	-0.00	$F_{8,9,12,12}$	0.16	$F_{9,9,12,12}$	-0.02
$F_{1,10,12,12}$	0.00	$F_{2,10,12,12}$	-0.00	$F_{3,10,12,12}$	0.00	$F_{4,10,12,12}$	0.01
$F_{5,10,12,12}$	0.00	$F_{6,10,12,12}$	-0.00	$F_{7,10,12,12}$	-0.02	$F_{8,10,12,12}$	0.00
$F_{9,10,12,12}$	-0.00	$F_{10,10,12,12}$	0.02	$F_{1,11,12,12}$	0.00	$F_{2,11,12,12}$	0.00
$F_{3,11,12,12}$	0.00	$F_{4,11,12,12}$	-0.00	$F_{5,11,12,12}$	-0.01	$F_{6,11,12,12}$	0.03
$F_{7,11,12,12}$	0.00	$F_{8,11,12,12}$	0.23	$F_{9,11,12,12}$	-0.18	$F_{10,11,12,12}$	-0.00
$F_{11,11,12,12}$	-0.22	$F_{1,12,12,12}$	-0.00	$F_{2,12,12,12}$	-0.00	$F_{3,12,12,12}$	0.00
$F_{4,12,12,12}$	0.00	$F_{5,12,12,12}$	-0.01	$F_{6,12,12,12}$	-0.02	$F_{7,12,12,12}$	-0.00
$F_{8,12,12,12}$	-0.15	$F_{9,12,12,12}$	0.01	$F_{10,12,12,12}$	0.00	$F_{11,12,12,12}$	0.16
$F_{12,12,12,12}$	0.01						

Table S17: The CcCR Force Constants for HSSH

$F_{1,1}$	0.035389	$F_{1,2}$	0.025097	$F_{1,3}$	-0.024657	$F_{1,4}$	-0.020648
$F_{1,5}$	-0.012017	$F_{1,6}$	0.011507	$F_{1,7}$	-0.014332	$F_{1,8}$	-0.012646
$F_{1,9}$	0.012252	$F_{1,10}$	-0.000408	$F_{1,11}$	-0.000435	$F_{1,12}$	0.000898
$F_{1,13}$	0.025097	$F_{2,1}$	0.132705	$F_{2,2}$	-0.128366	$F_{2,3}$	-0.025124
$F_{2,4}$	-0.135663	$F_{2,5}$	0.131307	$F_{2,6}$	0.000462	$F_{2,7}$	0.004713
$F_{2,8}$	-0.004840	$F_{2,9}$	-0.000435	$F_{2,10}$	-0.001755	$F_{2,11}$	0.001899
$F_{2,12}$	-0.024657	$F_{2,13}$	-0.128366	$F_{3,1}$	0.131327	$F_{3,2}$	0.024639
$F_{3,3}$	0.131267	$F_{3,4}$	-0.133790	$F_{3,5}$	0.000916	$F_{3,6}$	-0.001001
$F_{3,7}$	0.000755	$F_{3,8}$	-0.000898	$F_{3,9}$	-0.001899	$F_{3,10}$	0.001708
$F_{3,11}$	-0.020648	$F_{3,12}$	-0.025124	$F_{3,13}$	0.024639	$F_{4,1}$	0.189553
$F_{4,2}$	0.019266	$F_{4,3}$	-0.011489	$F_{4,4}$	-0.154572	$F_{4,5}$	0.005396
$F_{4,6}$	-0.012234	$F_{4,7}$	-0.014332	$F_{4,8}$	0.000462	$F_{4,9}$	-0.000916
$F_{4,10}$	-0.012017	$F_{4,11}$	-0.135663	$F_{4,12}$	0.131267	$F_{4,13}$	0.019266
$F_{5,1}$	0.150976	$F_{5,2}$	-0.134207	$F_{5,3}$	0.005396	$F_{5,4}$	-0.020025
$F_{5,5}$	0.001939	$F_{5,6}$	-0.012646	$F_{5,7}$	0.004713	$F_{5,8}$	0.001001
$F_{5,9}$	0.011507	$F_{5,10}$	0.131307	$F_{5,11}$	-0.133790	$F_{5,12}$	-0.011489
$F_{5,13}$	-0.134207	$F_{6,1}$	0.148767	$F_{6,2}$	0.012234	$F_{6,3}$	-0.001939
$F_{6,4}$	-0.015732	$F_{6,5}$	-0.012252	$F_{6,6}$	0.004840	$F_{6,7}$	0.000755
$F_{6,8}$	-0.014332	$F_{6,9}$	0.000462	$F_{6,10}$	0.000916	$F_{6,11}$	-0.154572
$F_{6,12}$	0.005396	$F_{6,13}$	0.012234	$F_{7,1}$	0.189553	$F_{7,2}$	0.019266
$F_{7,3}$	0.011489	$F_{7,4}$	-0.020648	$F_{7,5}$	-0.025124	$F_{7,6}$	-0.024639
$F_{7,7}$	-0.012646	$F_{7,8}$	0.004713	$F_{7,9}$	-0.001001	$F_{7,10}$	0.005396
$F_{7,11}$	-0.020025	$F_{7,12}$	-0.001939	$F_{7,13}$	0.019266	$F_{8,1}$	0.150976
$F_{8,2}$	0.134207	$F_{8,3}$	-0.012017	$F_{8,4}$	-0.135663	$F_{8,5}$	-0.131267
$F_{8,6}$	0.012252	$F_{8,7}$	-0.004840	$F_{8,8}$	0.000755	$F_{8,9}$	-0.012234
$F_{8,10}$	0.001939	$F_{8,11}$	-0.015732	$F_{8,12}$	0.011489	$F_{8,13}$	0.134207
$F_{9,1}$	0.148767	$F_{9,2}$	-0.011507	$F_{9,3}$	-0.131307	$F_{9,4}$	-0.133790
$F_{9,5}$	-0.000408	$F_{9,6}$	-0.000435	$F_{9,7}$	-0.000898	$F_{9,8}$	-0.014332
$F_{9,9}$	-0.012646	$F_{9,10}$	-0.012252	$F_{9,11}$	-0.020648	$F_{9,12}$	-0.012017
$F_{9,13}$	-0.011507	$F_{10,1}$	0.035389	$F_{10,2}$	0.025097	$F_{10,3}$	0.024657
$F_{10,4}$	-0.000435	$F_{10,5}$	-0.001755	$F_{10,6}$	-0.001899	$F_{10,7}$	0.000462
$F_{10,8}$	0.004713	$F_{10,9}$	0.004840	$F_{10,10}$	-0.025124	$F_{10,11}$	-0.135663
$F_{10,12}$	-0.131307	$F_{10,13}$	0.025097	$F_{11,1}$	0.132705	$F_{11,2}$	0.128366
$F_{11,3}$	0.000898	$F_{11,4}$	0.001899	$F_{11,5}$	0.001708	$F_{11,6}$	-0.000916
$F_{11,7}$	0.001001	$F_{11,8}$	0.000755	$F_{11,9}$	-0.024639	$F_{11,10}$	-0.131267
$F_{11,11}$	-0.133790	$F_{11,12}$	0.024657	$F_{11,13}$	0.128366	$F_{12,1}$	0.131327
$F_{1,1,1}$	0.0248	$F_{1,1,2}$	0.0380	$F_{1,2,2}$	-0.0661	$F_{2,2,2}$	-0.1493
$F_{1,1,3}$	-0.0370	$F_{1,2,3}$	0.0812	$F_{2,2,3}$	0.2918	$F_{1,3,3}$	-0.0651
$F_{2,3,3}$	-0.2902	$F_{3,3,3}$	0.1459	$F_{1,1,4}$	-0.0440	$F_{1,2,4}$	-0.0369
$F_{2,2,4}$	0.0696	$F_{1,3,4}$	0.0367	$F_{2,3,4}$	-0.0888	$F_{3,3,4}$	0.0692
$F_{1,4,4}$	0.0372	$F_{2,4,4}$	0.0303	$F_{3,4,4}$	-0.0301	$F_{4,4,4}$	-0.4571
$F_{1,1,5}$	-0.0433	$F_{1,2,5}$	0.0659	$F_{2,2,5}$	0.1483	$F_{1,3,5}$	-0.0731
$F_{2,3,5}$	-0.2924	$F_{3,3,5}$	0.2925	$F_{1,4,5}$	0.0308	$F_{2,4,5}$	-0.0642
$F_{3,4,5}$	0.0805	$F_{4,4,5}$	-0.0238	$F_{1,5,5}$	-0.0636	$F_{2,5,5}$	-0.1442
$F_{3,5,5}$	0.2968	$F_{4,5,5}$	0.0899	$F_{5,5,5}$	0.1407	$F_{1,1,6}$	0.0423
$F_{1,2,6}$	-0.0736	$F_{2,2,6}$	-0.2935	$F_{1,3,6}$	0.0651	$F_{2,3,6}$	0.2914
$F_{3,3,6}$	-0.1441	$F_{1,4,6}$	-0.0297	$F_{2,4,6}$	0.0807	$F_{3,4,6}$	-0.0655
$F_{4,4,6}$	0.0110	$F_{1,5,6}$	0.0659	$F_{2,5,6}$	0.2944	$F_{3,5,6}$	-0.2939
$F_{4,5,6}$	-0.0728	$F_{5,5,6}$	-0.2991	$F_{1,6,6}$	-0.0631	$F_{2,6,6}$	-0.2962
$F_{3,6,6}$	0.1387	$F_{4,6,6}$	0.0919	$F_{5,6,6}$	0.3005	$F_{6,6,6}$	-0.1296
$F_{1,1,7}$	0.0186	$F_{1,2,7}$	-0.0001	$F_{2,2,7}$	-0.0034	$F_{1,3,7}$	0.0021
$F_{2,3,7}$	0.0080	$F_{3,3,7}$	-0.0027	$F_{1,4,7}$	0.0074	$F_{2,4,7}$	0.0057
$F_{3,4,7}$	-0.0086	$F_{4,4,7}$	0.3933	$F_{1,5,7}$	0.0105	$F_{2,5,7}$	-0.0017
$F_{3,5,7}$	-0.0073	$F_{4,5,7}$	-0.0166	$F_{5,5,7}$	-0.0242	$F_{1,6,7}$	-0.0143
$F_{2,6,7}$	-0.0074	$F_{3,6,7}$	-0.0012	$F_{4,6,7}$	0.0086	$F_{5,6,7}$	0.0063
$F_{6,6,7}$	-0.0252	$F_{1,7,7}$	-0.0267	$F_{2,7,7}$	-0.0059	$F_{3,7,7}$	0.0074
$F_{4,7,7}$	-0.3933	$F_{5,7,7}$	0.0124	$F_{6,7,7}$	0.0117	$F_{7,7,7}$	0.4571
$F_{1,1,8}$	0.0052	$F_{1,2,8}$	0.0004	$F_{2,2,8}$	-0.0000	$F_{1,3,8}$	-0.0074
$F_{2,3,8}$	0.0005	$F_{3,3,8}$	-0.0014	$F_{1,4,8}$	0.0063	$F_{2,4,8}$	-0.0075
$F_{3,4,8}$	0.0054	$F_{4,4,8}$	-0.0124	$F_{1,5,8}$	-0.0025	$F_{2,5,8}$	-0.0024
$F_{3,5,8}$	-0.0034	$F_{4,5,8}$	-0.0183	$F_{5,5,8}$	0.0041	$F_{1,6,8}$	0.0070
$F_{2,6,8}$	-0.0008	$F_{3,6,8}$	0.0016	$F_{4,6,8}$	-0.0044	$F_{5,6,8}$	0.0034
$F_{6,6,8}$	-0.0069	$F_{1,7,8}$	-0.0095	$F_{2,7,8}$	0.0073	$F_{3,7,8}$	0.0021
$F_{4,7,8}$	0.0166	$F_{5,7,8}$	0.0183	$F_{6,7,8}$	-0.0099	$F_{7,7,8}$	0.0238
$F_{1,8,8}$	0.0021	$F_{2,8,8}$	0.0007	$F_{3,8,8}$	0.0010	$F_{4,8,8}$	0.0242
$F_{5,8,8}$	-0.0041	$F_{6,8,8}$	0.0012	$F_{7,8,8}$	-0.0899	$F_{8,8,8}$	-0.1407
$F_{1,1,9}$	-0.0058	$F_{1,2,9}$	-0.0082	$F_{2,2,9}$	0.0026	$F_{1,3,9}$	-0.0010
$F_{2,3,9}$	-0.0009	$F_{3,3,9}$	-0.0025	$F_{1,4,9}$	-0.0060	$F_{2,4,9}$	0.0110
$F_{3,4,9}$	-0.0013	$F_{4,4,9}$	0.0117	$F_{1,5,9}$	0.0073	$F_{2,5,9}$	-0.0038
$F_{3,5,9}$	0.0002	$F_{4,5,9}$	-0.0099	$F_{5,5,9}$	0.0012	$F_{1,6,9}$	-0.0011
$F_{2,6,9}$	0.0044	$F_{3,6,9}$	0.0062	$F_{4,6,9}$	-0.0251	$F_{5,6,9}$	-0.0055
$F_{6,6,9}$	-0.0062	$F_{1,7,9}$	0.0101	$F_{2,7,9}$	-0.0035	$F_{3,7,9}$	0.0014
$F_{4,7,9}$	0.0086	$F_{5,7,9}$	-0.0044	$F_{6,7,9}$	0.0251	$F_{7,7,9}$	0.0110
$F_{1,8,9}$	0.0005	$F_{2,8,9}$	0.0013	$F_{3,8,9}$	0.0011	$F_{4,8,9}$	0.0063

Table S18: The CcCR Force Constants for HSSH (cont.)

$F_{5,8,9}$	0.0034	$F_{6,8,9}$	0.0055	$F_{7,8,9}$	-0.0728	$F_{8,8,9}$	-0.2991
$F_{1,9,9}$	0.0036	$F_{2,9,9}$	-0.0026	$F_{3,9,9}$	-0.0029	$F_{4,9,9}$	0.0252
$F_{5,9,9}$	0.0069	$F_{6,9,9}$	-0.0062	$F_{7,9,9}$	-0.0919	$F_{8,9,9}$	-0.3005
$F_{9,9,9}$	-0.1296	$F_{1,1,10}$	0.0006	$F_{1,2,10}$	-0.0010	$F_{2,2,10}$	-0.0001
$F_{1,3,10}$	-0.0017	$F_{2,3,10}$	-0.0004	$F_{3,3,10}$	-0.0014	$F_{1,4,10}$	-0.0006
$F_{2,4,10}$	0.0009	$F_{3,4,10}$	0.0021	$F_{4,4,10}$	0.0267	$F_{1,5,10}$	0.0020
$F_{2,5,10}$	0.0001	$F_{3,5,10}$	-0.0001	$F_{4,5,10}$	0.0095	$F_{5,5,10}$	-0.0021
$F_{1,6,10}$	0.0017	$F_{2,6,10}$	0.0003	$F_{3,6,10}$	0.0015	$F_{4,6,10}$	0.0101
$F_{5,6,10}$	0.0005	$F_{6,6,10}$	-0.0036	$F_{1,7,10}$	0.0006	$F_{2,7,10}$	0.0003
$F_{3,7,10}$	-0.0009	$F_{4,7,10}$	-0.0074	$F_{5,7,10}$	-0.0063	$F_{6,7,10}$	-0.0060
$F_{7,7,10}$	-0.0372	$F_{1,8,10}$	-0.0020	$F_{2,8,10}$	-0.0002	$F_{3,8,10}$	-0.0002
$F_{4,8,10}$	-0.0105	$F_{5,8,10}$	0.0025	$F_{6,8,10}$	0.0073	$F_{7,8,10}$	-0.0308
$F_{8,8,10}$	0.0636	$F_{1,9,10}$	0.0017	$F_{2,9,10}$	0.0007	$F_{3,9,10}$	0.0009
$F_{4,9,10}$	-0.0143	$F_{5,9,10}$	0.0070	$F_{6,9,10}$	0.0011	$F_{7,9,10}$	-0.0297
$F_{8,9,10}$	0.0659	$F_{9,9,10}$	0.0631	$F_{1,10,10}$	-0.0006	$F_{2,10,10}$	-0.0001
$F_{3,10,10}$	0.0005	$F_{4,10,10}$	-0.0186	$F_{5,10,10}$	-0.0052	$F_{6,10,10}$	-0.0058
$F_{7,10,10}$	0.0440	$F_{8,10,10}$	0.0433	$F_{9,10,10}$	0.0423	$F_{10,10,10}$	-0.0248
$F_{1,1,11}$	0.0001	$F_{1,2,11}$	-0.0002	$F_{2,2,11}$	0.0010	$F_{1,3,11}$	-0.0007
$F_{2,3,11}$	0.0000	$F_{3,3,11}$	-0.0008	$F_{1,4,11}$	-0.0003	$F_{2,4,11}$	0.0022
$F_{3,4,11}$	0.0029	$F_{4,4,11}$	0.0059	$F_{1,5,11}$	0.0002	$F_{2,5,11}$	-0.0018
$F_{3,5,11}$	-0.0010	$F_{4,5,11}$	-0.0073	$F_{5,5,11}$	-0.0007	$F_{1,6,11}$	0.0007
$F_{2,6,11}$	-0.0000	$F_{3,6,11}$	0.0009	$F_{4,6,11}$	-0.0035	$F_{5,6,11}$	0.0013
$F_{6,6,11}$	0.0026	$F_{1,7,11}$	-0.0009	$F_{2,7,11}$	-0.0022	$F_{3,7,11}$	-0.0028
$F_{4,7,11}$	-0.0057	$F_{5,7,11}$	0.0075	$F_{6,7,11}$	0.0110	$F_{7,7,11}$	-0.0303
$F_{1,8,11}$	-0.0001	$F_{2,8,11}$	0.0018	$F_{3,8,11}$	0.0018	$F_{4,8,11}$	0.0017
$F_{5,8,11}$	0.0024	$F_{6,8,11}$	-0.0038	$F_{7,8,11}$	0.0642	$F_{8,8,11}$	0.1442
$F_{1,9,11}$	0.0003	$F_{2,9,11}$	-0.0000	$F_{3,9,11}$	-0.0003	$F_{4,9,11}$	-0.0074
$F_{5,9,11}$	-0.0008	$F_{6,9,11}$	-0.0044	$F_{7,9,11}$	0.0807	$F_{8,9,11}$	0.2944
$F_{9,9,11}$	0.2962	$F_{1,10,11}$	0.0010	$F_{2,10,11}$	0.0002	$F_{3,10,11}$	0.0006
$F_{4,10,11}$	0.0001	$F_{5,10,11}$	-0.0004	$F_{6,10,11}$	-0.0082	$F_{7,10,11}$	0.0369
$F_{8,10,11}$	-0.0659	$F_{9,10,11}$	-0.0736	$F_{10,10,11}$	-0.0380	$F_{1,11,11}$	0.0001
$F_{2,11,11}$	-0.0010	$F_{3,11,11}$	-0.0009	$F_{4,11,11}$	0.0034	$F_{5,11,11}$	0.0000
$F_{6,11,11}$	0.0026	$F_{7,11,11}$	-0.0696	$F_{8,11,11}$	-0.1483	$F_{9,11,11}$	-0.2935
$F_{10,11,11}$	0.0661	$F_{11,11,11}$	0.1493	$F_{1,1,12}$	0.0005	$F_{1,2,12}$	0.0006
$F_{2,2,12}$	-0.0009	$F_{1,3,12}$	0.0009	$F_{2,3,12}$	-0.0003	$F_{3,3,12}$	0.0008
$F_{1,4,12}$	-0.0009	$F_{2,4,12}$	-0.0028	$F_{3,4,12}$	-0.0025	$F_{4,4,12}$	0.0074
$F_{1,5,12}$	-0.0002	$F_{2,5,12}$	0.0018	$F_{3,5,12}$	0.0013	$F_{4,5,12}$	0.0021
$F_{5,5,12}$	0.0010	$F_{1,6,12}$	-0.0009	$F_{2,6,12}$	0.0003	$F_{3,6,12}$	-0.0008
$F_{4,6,12}$	-0.0014	$F_{5,6,12}$	-0.0011	$F_{6,6,12}$	-0.0029	$F_{1,7,12}$	0.0021
$F_{2,7,12}$	0.0029	$F_{3,7,12}$	0.0025	$F_{4,7,12}$	-0.0086	$F_{5,7,12}$	0.0054
$F_{6,7,12}$	0.0013	$F_{7,7,12}$	-0.0301	$F_{1,8,12}$	-0.0001	$F_{2,8,12}$	-0.0010
$F_{3,8,12}$	-0.0013	$F_{4,8,12}$	-0.0073	$F_{5,8,12}$	-0.0034	$F_{6,8,12}$	-0.0002
$F_{7,8,12}$	0.0805	$F_{8,8,12}$	0.2968	$F_{1,9,12}$	-0.0015	$F_{2,9,12}$	-0.0009
$F_{3,9,12}$	-0.0008	$F_{4,9,12}$	0.0012	$F_{5,9,12}$	-0.0016	$F_{6,9,12}$	0.0062
$F_{7,9,12}$	0.0655	$F_{8,9,12}$	0.2939	$F_{9,9,12}$	0.1387	$F_{1,10,12}$	-0.0017
$F_{2,10,12}$	-0.0007	$F_{3,10,12}$	-0.0009	$F_{4,10,12}$	0.0021	$F_{5,10,12}$	-0.0074
$F_{6,10,12}$	0.0010	$F_{7,10,12}$	0.0367	$F_{8,10,12}$	-0.0731	$F_{9,10,12}$	-0.0651
$F_{10,10,12}$	-0.0370	$F_{1,11,12}$	-0.0004	$F_{2,11,12}$	0.0000	$F_{3,11,12}$	0.0003
$F_{4,11,12}$	0.0080	$F_{5,11,12}$	0.0005	$F_{6,11,12}$	0.0009	$F_{7,11,12}$	-0.0888
$F_{8,11,12}$	-0.2924	$F_{9,11,12}$	-0.2914	$F_{10,11,12}$	0.0812	$F_{11,11,12}$	0.2918
$F_{1,12,12}$	0.0014	$F_{2,12,12}$	0.0008	$F_{3,12,12}$	0.0008	$F_{4,12,12}$	0.0027
$F_{5,12,12}$	0.0014	$F_{6,12,12}$	-0.0025	$F_{7,12,12}$	-0.0692	$F_{8,12,12}$	-0.2925
$F_{9,12,12}$	-0.1441	$F_{10,12,12}$	0.0651	$F_{11,12,12}$	0.2902	$F_{12,12,12}$	0.1459
$F_{1,1,1,1}$	0.02	$F_{1,1,1,2}$	-0.12	$F_{1,1,2,2}$	-0.12	$F_{1,2,2,2}$	0.09
$F_{2,2,2,2}$	-0.12	$F_{1,1,1,3}$	0.11	$F_{1,1,2,3}$	0.14	$F_{1,2,2,3}$	-0.18
$F_{2,2,2,3}$	-0.38	$F_{1,1,3,3}$	-0.12	$F_{1,2,3,3}$	0.18	$F_{2,2,3,3}$	0.62
$F_{1,3,3,3}$	-0.09	$F_{2,3,3,3}$	-0.37	$F_{3,3,3,3}$	-0.12	$F_{1,1,1,4}$	-0.01
$F_{1,1,2,4}$	0.13	$F_{1,2,2,4}$	0.11	$F_{2,2,2,4}$	-0.08	$F_{1,1,3,4}$	-0.13
$F_{1,2,3,4}$	-0.14	$F_{2,2,3,4}$	0.18	$F_{1,3,3,4}$	0.12	$F_{2,3,3,4}$	-0.18
$F_{3,3,3,4}$	0.08	$F_{1,1,4,4}$	0.01	$F_{1,2,4,4}$	-0.14	$F_{2,2,4,4}$	-0.12
$F_{1,3,4,4}$	0.14	$F_{2,3,4,4}$	0.14	$F_{3,3,4,4}$	-0.12	$F_{1,4,4,4}$	0.01
$F_{2,4,4,4}$	0.16	$F_{3,4,4,4}$	-0.16	$F_{4,4,4,4}$	0.81	$F_{1,1,1,5}$	0.11
$F_{1,1,2,5}$	0.12	$F_{1,2,2,5}$	-0.10	$F_{2,2,2,5}$	0.12	$F_{1,1,3,5}$	-0.14
$F_{1,2,3,5}$	0.18	$F_{2,2,3,5}$	0.38	$F_{1,3,3,5}$	-0.18	$F_{2,3,3,5}$	-0.62
$F_{3,3,3,5}$	0.37	$F_{1,1,4,5}$	-0.12	$F_{1,2,4,5}$	-0.11	$F_{2,2,4,5}$	0.08
$F_{1,3,4,5}$	0.13	$F_{2,3,4,5}$	-0.18	$F_{3,3,4,5}$	0.18	$F_{1,4,4,5}$	0.13
$F_{2,4,4,5}$	0.11	$F_{3,4,4,5}$	-0.14	$F_{4,4,4,5}$	-0.17	$F_{1,1,5,5}$	-0.13
$F_{1,2,5,5}$	0.10	$F_{2,2,5,5}$	-0.12	$F_{1,3,5,5}$	-0.18	$F_{2,3,5,5}$	-0.38
$F_{3,3,5,5}$	0.62	$F_{1,4,5,5}$	0.12	$F_{2,4,5,5}$	-0.09	$F_{3,4,5,5}$	0.17
$F_{4,4,5,5}$	-0.22	$F_{1,5,5,5}$	-0.12	$F_{2,5,5,5}$	0.13	$F_{3,5,5,5}$	0.38
$F_{4,5,5,5}$	0.10	$F_{5,5,5,5}$	-0.14	$F_{1,1,1,6}$	-0.11	$F_{1,1,2,6}$	-0.14
$F_{1,2,2,6}$	0.18	$F_{2,2,2,6}$	0.38	$F_{1,1,3,6}$	0.12	$F_{1,2,3,6}$	-0.18
$F_{2,2,3,6}$	-0.62	$F_{1,3,3,6}$	0.10	$F_{2,3,3,6}$	0.37	$F_{3,3,3,6}$	0.12
$F_{1,1,4,6}$	0.12	$F_{1,2,4,6}$	0.13	$F_{2,2,4,6}$	-0.18	$F_{1,3,4,6}$	-0.12

Table S19: The CcCR Force Constants for HSSH (cont.)

$F_{2,3,4,6}$	0.18	$F_{3,3,4,6}$	-0.09	$F_{1,4,4,6}$	-0.13	$F_{2,4,4,6}$	-0.14
$F_{3,4,4,6}$	0.12	$F_{4,4,4,6}$	0.18	$F_{1,1,5,6}$	0.14	$F_{1,2,5,6}$	-0.18
$F_{2,2,5,6}$	-0.38	$F_{1,3,5,6}$	0.17	$F_{2,3,5,6}$	0.62	$F_{3,3,5,6}$	-0.37
$F_{1,4,5,6}$	-0.13	$F_{2,4,5,6}$	0.17	$F_{3,4,5,6}$	-0.17	$F_{4,4,5,6}$	0.13
$F_{1,5,5,6}$	0.18	$F_{2,5,5,6}$	0.37	$F_{3,5,5,6}$	-0.63	$F_{4,5,5,6}$	-0.17
$F_{5,5,5,6}$	-0.37	$F_{1,1,6,6}$	-0.12	$F_{1,2,6,6}$	0.18	$F_{2,2,6,6}$	0.63
$F_{1,3,6,6}$	-0.10	$F_{2,3,6,6}$	-0.38	$F_{3,3,6,6}$	-0.13	$F_{1,4,6,6}$	0.12
$F_{2,4,6,6}$	-0.18	$F_{3,4,6,6}$	0.10	$F_{4,4,6,6}$	-0.23	$F_{1,5,6,6}$	-0.17
$F_{2,5,6,6}$	-0.63	$F_{3,5,6,6}$	0.37	$F_{4,5,6,6}$	0.17	$F_{5,5,6,6}$	0.64
$F_{1,6,6,6}$	0.12	$F_{2,6,6,6}$	0.38	$F_{3,6,6,6}$	0.14	$F_{4,6,6,6}$	-0.13
$F_{5,6,6,6}$	-0.37	$F_{6,6,6,6}$	-0.14	$F_{1,1,1,7}$	-0.02	$F_{1,1,2,7}$	-0.01
$F_{1,2,2,7}$	0.00	$F_{2,2,2,7}$	-0.01	$F_{1,1,3,7}$	0.01	$F_{1,2,3,7}$	-0.00
$F_{2,2,3,7}$	-0.00	$F_{1,3,3,7}$	0.00	$F_{2,3,3,7}$	0.00	$F_{3,3,3,7}$	0.01
$F_{1,1,4,7}$	0.00	$F_{1,2,4,7}$	0.01	$F_{2,2,4,7}$	0.01	$F_{1,3,4,7}$	-0.01
$F_{2,3,4,7}$	-0.01	$F_{3,3,4,7}$	0.00	$F_{1,4,4,7}$	-0.02	$F_{2,4,4,7}$	-0.03
$F_{3,4,4,7}$	0.03	$F_{4,4,4,7}$	-0.79	$F_{1,1,5,7}$	0.00	$F_{1,2,5,7}$	-0.00
$F_{2,2,5,7}$	0.02	$F_{1,3,5,7}$	0.01	$F_{2,3,5,7}$	-0.00	$F_{3,3,5,7}$	0.00
$F_{1,4,5,7}$	-0.01	$F_{2,4,5,7}$	0.00	$F_{3,4,5,7}$	0.00	$F_{4,4,5,7}$	0.05
$F_{1,5,5,7}$	0.01	$F_{2,5,5,7}$	-0.02	$F_{3,5,5,7}$	0.00	$F_{4,5,5,7}$	0.10
$F_{5,5,5,7}$	0.01	$F_{1,1,6,7}$	-0.01	$F_{1,2,6,7}$	0.01	$F_{2,2,6,7}$	0.00
$F_{1,3,6,7}$	-0.00	$F_{2,3,6,7}$	-0.00	$F_{3,3,6,7}$	-0.01	$F_{1,4,6,7}$	0.02
$F_{2,4,6,7}$	0.00	$F_{3,4,6,7}$	0.00	$F_{4,4,6,7}$	-0.04	$F_{1,5,6,7}$	-0.01
$F_{2,5,6,7}$	0.00	$F_{3,5,6,7}$	-0.00	$F_{4,5,6,7}$	0.00	$F_{5,5,6,7}$	-0.01
$F_{1,6,6,7}$	0.01	$F_{2,6,6,7}$	0.00	$F_{3,6,6,7}$	0.00	$F_{4,6,6,7}$	0.10
$F_{5,6,6,7}$	0.00	$F_{6,6,6,7}$	-0.00	$F_{1,1,7,7}$	0.01	$F_{1,2,7,7}$	0.00
$F_{2,2,7,7}$	-0.00	$F_{1,3,7,7}$	-0.00	$F_{2,3,7,7}$	0.01	$F_{3,3,7,7}$	-0.01
$F_{1,4,7,7}$	0.02	$F_{2,4,7,7}$	0.02	$F_{3,4,7,7}$	-0.02	$F_{4,4,7,7}$	0.79
$F_{1,5,7,7}$	0.01	$F_{2,5,7,7}$	-0.01	$F_{3,5,7,7}$	-0.01	$F_{4,5,7,7}$	-0.04
$F_{5,5,7,7}$	-0.10	$F_{1,6,7,7}$	-0.01	$F_{2,6,7,7}$	-0.01	$F_{3,6,7,7}$	0.00
$F_{4,6,7,7}$	0.01	$F_{5,6,7,7}$	0.00	$F_{6,6,7,7}$	-0.11	$F_{1,7,7,7}$	-0.04
$F_{2,7,7,7}$	-0.02	$F_{3,7,7,7}$	0.03	$F_{4,7,7,7}$	-0.79	$F_{5,7,7,7}$	0.03
$F_{6,7,7,7}$	-0.01	$F_{7,7,7,7}$	0.81	$F_{1,1,1,8}$	0.01	$F_{1,1,2,8}$	-0.00
$F_{1,2,2,8}$	0.01	$F_{2,2,2,8}$	-0.00	$F_{1,1,3,8}$	0.00	$F_{1,2,3,8}$	0.00
$F_{2,2,3,8}$	-0.00	$F_{1,3,3,8}$	-0.01	$F_{2,3,3,8}$	-0.00	$F_{3,3,3,8}$	0.00
$F_{1,1,4,8}$	-0.01	$F_{1,2,4,8}$	-0.00	$F_{2,2,4,8}$	-0.00	$F_{1,3,4,8}$	0.00
$F_{2,3,4,8}$	-0.00	$F_{3,3,4,8}$	0.01	$F_{1,4,4,8}$	0.01	$F_{2,4,4,8}$	0.01
$F_{3,4,4,8}$	-0.00	$F_{4,4,4,8}$	0.03	$F_{1,1,5,8}$	0.01	$F_{1,2,5,8}$	-0.01
$F_{2,2,5,8}$	0.01	$F_{1,3,5,8}$	-0.00	$F_{2,3,5,8}$	0.00	$F_{3,3,5,8}$	-0.00
$F_{1,4,5,8}$	0.00	$F_{2,4,5,8}$	0.01	$F_{3,4,5,8}$	0.00	$F_{4,4,5,8}$	0.10
$F_{1,5,5,8}$	0.02	$F_{2,5,5,8}$	-0.01	$F_{3,5,5,8}$	-0.00	$F_{4,5,5,8}$	-0.02
$F_{5,5,5,8}$	0.01	$F_{1,1,6,8}$	-0.00	$F_{1,2,6,8}$	-0.00	$F_{2,2,6,8}$	-0.00
$F_{1,3,6,8}$	0.01	$F_{2,3,6,8}$	0.00	$F_{3,3,6,8}$	-0.00	$F_{1,4,6,8}$	-0.00
$F_{2,4,6,8}$	0.00	$F_{3,4,6,8}$	-0.01	$F_{4,4,6,8}$	0.00	$F_{1,5,6,8}$	-0.00
$F_{2,5,6,8}$	0.00	$F_{3,5,6,8}$	0.00	$F_{4,5,6,8}$	-0.00	$F_{5,5,6,8}$	0.00
$F_{1,6,6,8}$	-0.00	$F_{2,6,6,8}$	-0.00	$F_{3,6,6,8}$	0.00	$F_{4,6,6,8}$	0.01
$F_{5,6,6,8}$	-0.01	$F_{6,6,6,8}$	-0.00	$F_{1,1,7,8}$	0.01	$F_{1,2,7,8}$	0.00
$F_{2,2,7,8}$	-0.00	$F_{1,3,7,8}$	-0.01	$F_{2,3,7,8}$	0.00	$F_{3,3,7,8}$	-0.00
$F_{1,4,7,8}$	0.00	$F_{2,4,7,8}$	-0.01	$F_{3,4,7,8}$	0.00	$F_{4,4,7,8}$	-0.04
$F_{1,5,7,8}$	-0.01	$F_{2,5,7,8}$	0.00	$F_{3,5,7,8}$	-0.00	$F_{4,5,7,8}$	-0.09
$F_{5,5,7,8}$	0.01	$F_{1,6,7,8}$	0.00	$F_{2,6,7,8}$	-0.00	$F_{3,6,7,8}$	0.00
$F_{4,6,7,8}$	0.00	$F_{5,6,7,8}$	0.00	$F_{6,6,7,8}$	-0.00	$F_{1,7,7,8}$	-0.01
$F_{2,7,7,8}$	0.01	$F_{3,7,7,8}$	0.00	$F_{4,7,7,8}$	0.05	$F_{5,7,7,8}$	0.10
$F_{6,7,7,8}$	-0.01	$F_{7,7,7,8}$	-0.17	$F_{1,1,8,8}$	-0.01	$F_{1,2,8,8}$	0.00
$F_{2,2,8,8}$	-0.00	$F_{1,3,8,8}$	-0.00	$F_{2,3,8,8}$	-0.00	$F_{3,3,8,8}$	0.00
$F_{1,4,8,8}$	-0.01	$F_{2,4,8,8}$	-0.00	$F_{3,4,8,8}$	0.01	$F_{4,4,8,8}$	-0.10
$F_{1,5,8,8}$	-0.01	$F_{2,5,8,8}$	0.01	$F_{3,5,8,8}$	0.00	$F_{4,5,8,8}$	0.01
$F_{5,5,8,8}$	-0.01	$F_{1,6,8,8}$	0.01	$F_{2,6,8,8}$	0.00	$F_{3,6,8,8}$	-0.00
$F_{4,6,8,8}$	-0.01	$F_{5,6,8,8}$	-0.01	$F_{6,6,8,8}$	0.01	$F_{1,7,8,8}$	0.01
$F_{2,7,8,8}$	0.00	$F_{3,7,8,8}$	-0.00	$F_{4,7,8,8}$	0.10	$F_{5,7,8,8}$	-0.02
$F_{6,7,8,8}$	0.01	$F_{7,7,8,8}$	-0.22	$F_{1,8,8,8}$	0.01	$F_{2,8,8,8}$	-0.01
$F_{3,8,8,8}$	0.00	$F_{4,8,8,8}$	0.01	$F_{5,8,8,8}$	0.01	$F_{6,8,8,8}$	0.01
$F_{7,8,8,8}$	0.10	$F_{8,8,8,8}$	-0.14	$F_{1,1,1,9}$	-0.00	$F_{1,1,2,9}$	0.01
$F_{1,2,2,9}$	0.00	$F_{2,2,2,9}$	0.00	$F_{1,1,3,9}$	0.00	$F_{1,2,3,9}$	-0.00
$F_{2,2,3,9}$	0.00	$F_{1,3,3,9}$	-0.01	$F_{2,3,3,9}$	0.00	$F_{3,3,3,9}$	-0.01
$F_{1,1,4,9}$	0.01	$F_{1,2,4,9}$	0.00	$F_{2,2,4,9}$	-0.01	$F_{1,3,4,9}$	-0.00
$F_{2,3,4,9}$	0.00	$F_{3,3,4,9}$	0.01	$F_{1,4,4,9}$	-0.01	$F_{2,4,4,9}$	-0.01
$F_{3,4,4,9}$	0.01	$F_{4,4,4,9}$	0.01	$F_{1,1,5,9}$	-0.00	$F_{1,2,5,9}$	-0.00
$F_{2,2,5,9}$	-0.00	$F_{1,3,5,9}$	0.00	$F_{2,3,5,9}$	-0.00	$F_{3,3,5,9}$	-0.01
$F_{1,4,5,9}$	-0.00	$F_{2,4,5,9}$	0.01	$F_{3,4,5,9}$	-0.00	$F_{4,4,5,9}$	0.01
$F_{1,5,5,9}$	0.00	$F_{2,5,5,9}$	0.01	$F_{3,5,5,9}$	0.01	$F_{4,5,5,9}$	-0.01
$F_{5,5,5,9}$	-0.01	$F_{1,1,6,9}$	0.01	$F_{1,2,6,9}$	0.00	$F_{2,2,6,9}$	-0.01
$F_{1,3,6,9}$	0.01	$F_{2,3,6,9}$	0.00	$F_{3,3,6,9}$	0.01	$F_{1,4,6,9}$	-0.00
$F_{2,4,6,9}$	-0.00	$F_{3,4,6,9}$	-0.01	$F_{4,4,6,9}$	0.11	$F_{1,5,6,9}$	0.00
$F_{2,5,6,9}$	0.01	$F_{3,5,6,9}$	0.00	$F_{4,5,6,9}$	0.00	$F_{5,5,6,9}$	-0.01

Table S20: The CcCR Force Constants for HSSH (cont.)

$F_{1,6,6,9}$	-0.02	$F_{2,6,6,9}$	-0.00	$F_{3,6,6,9}$	-0.01	$F_{4,6,6,9}$	0.02
$F_{5,6,6,9}$	-0.00	$F_{6,6,6,9}$	0.00	$F_{1,1,7,9}$	-0.01	$F_{1,2,7,9}$	-0.01
$F_{2,2,7,9}$	0.00	$F_{1,3,7,9}$	0.00	$F_{2,3,7,9}$	0.00	$F_{3,3,7,9}$	-0.00
$F_{1,4,7,9}$	-0.01	$F_{2,4,7,9}$	0.01	$F_{3,4,7,9}$	-0.00	$F_{4,4,7,9}$	-0.01
$F_{1,5,7,9}$	0.01	$F_{2,5,7,9}$	-0.01	$F_{3,5,7,9}$	-0.00	$F_{4,5,7,9}$	-0.00
$F_{5,5,7,9}$	0.01	$F_{1,6,7,9}$	-0.01	$F_{2,6,7,9}$	0.00	$F_{3,6,7,9}$	0.01
$F_{4,6,7,9}$	-0.10	$F_{5,6,7,9}$	-0.00	$F_{6,6,7,9}$	0.00	$F_{1,7,7,9}$	0.01
$F_{2,7,7,9}$	-0.00	$F_{3,7,7,9}$	0.00	$F_{4,7,7,9}$	0.04	$F_{5,7,7,9}$	-0.00
$F_{6,7,7,9}$	0.11	$F_{7,7,7,9}$	-0.18	$F_{1,1,8,9}$	0.00	$F_{1,2,8,9}$	0.00
$F_{2,2,8,9}$	0.00	$F_{1,3,8,9}$	-0.00	$F_{2,3,8,9}$	0.00	$F_{3,3,8,9}$	0.00
$F_{1,4,8,9}$	-0.00	$F_{2,4,8,9}$	-0.00	$F_{3,4,8,9}$	0.00	$F_{4,4,8,9}$	-0.00
$F_{1,5,8,9}$	0.00	$F_{2,5,8,9}$	-0.01	$F_{3,5,8,9}$	-0.00	$F_{4,5,8,9}$	-0.00
$F_{5,5,8,9}$	0.01	$F_{1,6,8,9}$	-0.00	$F_{2,6,8,9}$	-0.00	$F_{3,6,8,9}$	-0.00
$F_{4,6,8,9}$	-0.00	$F_{5,6,8,9}$	0.01	$F_{6,6,8,9}$	0.00	$F_{1,7,8,9}$	0.00
$F_{2,7,8,9}$	0.00	$F_{3,7,8,9}$	-0.00	$F_{4,7,8,9}$	-0.00	$F_{5,7,8,9}$	0.00
$F_{6,7,8,9}$	0.00	$F_{7,7,8,9}$	-0.13	$F_{1,8,8,9}$	0.00	$F_{2,8,8,9}$	0.00
$F_{3,8,8,9}$	-0.00	$F_{4,8,8,9}$	0.01	$F_{5,8,8,9}$	-0.00	$F_{6,8,8,9}$	-0.01
$F_{7,8,8,9}$	0.17	$F_{8,8,8,9}$	0.37	$F_{1,1,9,9}$	-0.01	$F_{1,2,9,9}$	0.00
$F_{2,2,9,9}$	0.00	$F_{1,3,9,9}$	0.00	$F_{2,3,9,9}$	-0.00	$F_{3,3,9,9}$	-0.00
$F_{1,4,9,9}$	0.00	$F_{2,4,9,9}$	-0.00	$F_{3,4,9,9}$	0.00	$F_{4,4,9,9}$	-0.11
$F_{1,5,9,9}$	-0.00	$F_{2,5,9,9}$	0.00	$F_{3,5,9,9}$	0.00	$F_{4,5,9,9}$	-0.00
$F_{5,5,9,9}$	0.01	$F_{1,6,9,9}$	0.01	$F_{2,6,9,9}$	-0.00	$F_{3,6,9,9}$	0.00
$F_{4,6,9,9}$	-0.00	$F_{5,6,9,9}$	-0.00	$F_{6,6,9,9}$	0.01	$F_{1,7,9,9}$	0.01
$F_{2,7,9,9}$	-0.00	$F_{3,7,9,9}$	-0.01	$F_{4,7,9,9}$	0.10	$F_{5,7,9,9}$	0.01
$F_{6,7,9,9}$	-0.02	$F_{7,7,9,9}$	-0.23	$F_{1,8,9,9}$	0.00	$F_{2,8,9,9}$	-0.00
$F_{3,8,9,9}$	-0.00	$F_{4,8,9,9}$	0.00	$F_{5,8,9,9}$	-0.01	$F_{6,8,9,9}$	0.00
$F_{7,8,9,9}$	0.17	$F_{8,8,9,9}$	0.64	$F_{1,9,9,9}$	-0.01	$F_{2,9,9,9}$	0.00
$F_{3,9,9,9}$	-0.00	$F_{4,9,9,9}$	0.00	$F_{5,9,9,9}$	0.00	$F_{6,9,9,9}$	0.00
$F_{7,9,9,9}$	0.13	$F_{8,9,9,9}$	0.37	$F_{9,9,9,9}$	-0.14	$F_{1,1,1,10}$	0.00
$F_{1,1,2,10}$	0.00	$F_{1,2,2,10}$	0.00	$F_{2,2,2,10}$	0.00	$F_{1,1,3,10}$	0.00
$F_{1,2,3,10}$	-0.00	$F_{2,2,3,10}$	0.00	$F_{1,3,3,10}$	-0.00	$F_{2,3,3,10}$	-0.00
$F_{3,3,3,10}$	-0.00	$F_{1,1,4,10}$	-0.00	$F_{1,2,4,10}$	-0.00	$F_{2,2,4,10}$	0.00
$F_{1,3,4,10}$	0.00	$F_{2,3,4,10}$	-0.00	$F_{3,3,4,10}$	0.00	$F_{1,4,4,10}$	0.00
$F_{2,4,4,10}$	0.00	$F_{3,4,4,10}$	-0.00	$F_{4,4,4,10}$	-0.04	$F_{1,1,5,10}$	0.00
$F_{1,2,5,10}$	-0.00	$F_{2,2,5,10}$	-0.00	$F_{1,3,5,10}$	0.00	$F_{2,3,5,10}$	-0.00
$F_{3,3,5,10}$	-0.00	$F_{1,4,5,10}$	-0.00	$F_{2,4,5,10}$	-0.00	$F_{3,4,5,10}$	0.00
$F_{4,4,5,10}$	-0.01	$F_{1,5,5,10}$	0.00	$F_{2,5,5,10}$	0.00	$F_{3,5,5,10}$	0.00
$F_{4,5,5,10}$	0.01	$F_{5,5,5,10}$	0.01	$F_{1,1,6,10}$	0.00	$F_{1,2,6,10}$	-0.00
$F_{2,2,6,10}$	-0.00	$F_{1,3,6,10}$	0.00	$F_{2,3,6,10}$	0.00	$F_{3,3,6,10}$	0.00
$F_{1,4,6,10}$	-0.00	$F_{2,4,6,10}$	0.00	$F_{3,4,6,10}$	-0.00	$F_{4,4,6,10}$	-0.01
$F_{1,5,6,10}$	-0.00	$F_{2,5,6,10}$	0.00	$F_{3,5,6,10}$	-0.00	$F_{4,5,6,10}$	-0.00
$F_{5,5,6,10}$	-0.00	$F_{1,6,6,10}$	-0.00	$F_{2,6,6,10}$	-0.00	$F_{3,6,6,10}$	-0.00
$F_{4,6,6,10}$	0.01	$F_{5,6,6,10}$	0.00	$F_{6,6,6,10}$	0.01	$F_{1,1,7,10}$	0.00
$F_{1,2,7,10}$	0.00	$F_{2,2,7,10}$	-0.00	$F_{1,3,7,10}$	-0.00	$F_{2,3,7,10}$	0.00
$F_{3,3,7,10}$	-0.00	$F_{1,4,7,10}$	-0.01	$F_{2,4,7,10}$	-0.00	$F_{3,4,7,10}$	0.00
$F_{4,4,7,10}$	0.02	$F_{1,5,7,10}$	0.00	$F_{2,5,7,10}$	0.01	$F_{3,5,7,10}$	-0.00
$F_{4,5,7,10}$	0.00	$F_{5,5,7,10}$	-0.01	$F_{1,6,7,10}$	-0.00	$F_{2,6,7,10}$	-0.00
$F_{3,6,7,10}$	0.00	$F_{4,6,7,10}$	0.01	$F_{5,6,7,10}$	0.00	$F_{6,6,7,10}$	0.00
$F_{1,7,7,10}$	0.00	$F_{2,7,7,10}$	0.00	$F_{3,7,7,10}$	-0.00	$F_{4,7,7,10}$	-0.02
$F_{5,7,7,10}$	0.01	$F_{6,7,7,10}$	0.01	$F_{7,7,7,10}$	0.01	$F_{1,1,8,10}$	-0.00
$F_{1,2,8,10}$	0.00	$F_{2,2,8,10}$	0.00	$F_{1,3,8,10}$	-0.00	$F_{2,3,8,10}$	-0.00
$F_{3,3,8,10}$	0.00	$F_{1,4,8,10}$	0.00	$F_{2,4,8,10}$	0.00	$F_{3,4,8,10}$	0.00
$F_{4,4,8,10}$	0.01	$F_{1,5,8,10}$	-0.00	$F_{2,5,8,10}$	-0.00	$F_{3,5,8,10}$	0.00
$F_{4,5,8,10}$	-0.01	$F_{5,5,8,10}$	-0.01	$F_{1,6,8,10}$	0.00	$F_{2,6,8,10}$	0.00
$F_{3,6,8,10}$	0.00	$F_{4,6,8,10}$	-0.01	$F_{5,6,8,10}$	-0.00	$F_{6,6,8,10}$	-0.00
$F_{1,7,8,10}$	-0.00	$F_{2,7,8,10}$	-0.00	$F_{3,7,8,10}$	0.00	$F_{4,7,8,10}$	-0.01
$F_{5,7,8,10}$	0.00	$F_{6,7,8,10}$	0.00	$F_{7,7,8,10}$	0.13	$F_{1,8,8,10}$	0.00
$F_{2,8,8,10}$	0.00	$F_{3,8,8,10}$	0.00	$F_{4,8,8,10}$	0.01	$F_{5,8,8,10}$	0.02
$F_{6,8,8,10}$	-0.00	$F_{7,8,8,10}$	0.12	$F_{8,8,8,10}$	-0.12	$F_{1,1,9,10}$	-0.00
$F_{1,2,9,10}$	0.00	$F_{2,2,9,10}$	0.00	$F_{1,3,9,10}$	0.00	$F_{2,3,9,10}$	-0.00
$F_{3,3,9,10}$	0.00	$F_{1,4,9,10}$	0.00	$F_{2,4,9,10}$	-0.00	$F_{3,4,9,10}$	-0.00
$F_{4,4,9,10}$	0.01	$F_{1,5,9,10}$	-0.00	$F_{2,5,9,10}$	0.00	$F_{3,5,9,10}$	0.00
$F_{4,5,9,10}$	-0.00	$F_{5,5,9,10}$	-0.01	$F_{1,6,9,10}$	0.00	$F_{2,6,9,10}$	0.00
$F_{3,6,9,10}$	-0.00	$F_{4,6,9,10}$	-0.01	$F_{5,6,9,10}$	-0.00	$F_{6,6,9,10}$	-0.01
$F_{1,7,9,10}$	0.00	$F_{2,7,9,10}$	0.00	$F_{3,7,9,10}$	-0.00	$F_{4,7,9,10}$	-0.02
$F_{5,7,9,10}$	0.00	$F_{6,7,9,10}$	-0.00	$F_{7,7,9,10}$	0.13	$F_{1,8,9,10}$	0.00
$F_{2,8,9,10}$	0.00	$F_{3,8,9,10}$	-0.00	$F_{4,8,9,10}$	0.01	$F_{5,8,9,10}$	0.00
$F_{6,8,9,10}$	0.00	$F_{7,8,9,10}$	0.13	$F_{8,8,9,10}$	-0.18	$F_{1,9,9,10}$	-0.00
$F_{2,9,9,10}$	0.00	$F_{3,9,9,10}$	-0.00	$F_{4,9,9,10}$	0.01	$F_{5,9,9,10}$	-0.00
$F_{6,9,9,10}$	0.02	$F_{7,9,9,10}$	0.12	$F_{8,9,9,10}$	-0.17	$F_{9,9,9,10}$	-0.12
$F_{1,1,10,10}$	-0.00	$F_{1,2,10,10}$	-0.00	$F_{2,2,10,10}$	0.00	$F_{1,3,10,10}$	-0.00
$F_{2,3,10,10}$	-0.00	$F_{3,3,10,10}$	0.00	$F_{1,4,10,10}$	0.00	$F_{2,4,10,10}$	0.00
$F_{3,4,10,10}$	-0.00	$F_{4,4,10,10}$	0.01	$F_{1,5,10,10}$	-0.00	$F_{2,5,10,10}$	-0.00
$F_{3,5,10,10}$	0.00	$F_{4,5,10,10}$	0.01	$F_{5,5,10,10}$	-0.01	$F_{1,6,10,10}$	0.00

Table S21: The CcCR Force Constants for HSSH (cont.)

$F_{2,6,10,10}$	0.00	$F_{3,6,10,10}$	0.00	$F_{4,6,10,10}$	0.01	$F_{5,6,10,10}$	-0.00
$F_{6,6,10,10}$	-0.01	$F_{1,7,10,10}$	-0.00	$F_{2,7,10,10}$	-0.00	$F_{3,7,10,10}$	0.00
$F_{4,7,10,10}$	0.00	$F_{5,7,10,10}$	-0.01	$F_{6,7,10,10}$	-0.01	$F_{7,7,10,10}$	0.01
$F_{1,8,10,10}$	0.00	$F_{2,8,10,10}$	0.00	$F_{3,8,10,10}$	-0.00	$F_{4,8,10,10}$	0.00
$F_{5,8,10,10}$	0.01	$F_{6,8,10,10}$	0.00	$F_{7,8,10,10}$	-0.12	$F_{8,8,10,10}$	-0.13
$F_{1,9,10,10}$	-0.00	$F_{2,9,10,10}$	0.00	$F_{3,9,10,10}$	0.00	$F_{4,9,10,10}$	0.01
$F_{5,9,10,10}$	0.00	$F_{6,9,10,10}$	0.01	$F_{7,9,10,10}$	-0.12	$F_{8,9,10,10}$	-0.14
$F_{9,9,10,10}$	-0.12	$F_{1,10,10,10}$	0.00	$F_{2,10,10,10}$	0.00	$F_{3,10,10,10}$	0.00
$F_{4,10,10,10}$	-0.02	$F_{5,10,10,10}$	0.01	$F_{6,10,10,10}$	0.00	$F_{7,10,10,10}$	-0.01
$F_{8,10,10,10}$	0.11	$F_{9,10,10,10}$	0.11	$F_{10,10,10,10}$	0.02	$F_{1,1,1,11}$	0.00
$F_{1,1,2,11}$	0.00	$F_{1,2,2,11}$	0.00	$F_{2,2,2,11}$	0.00	$F_{1,1,3,11}$	0.00
$F_{1,2,3,11}$	-0.00	$F_{2,2,3,11}$	0.00	$F_{1,3,3,11}$	0.00	$F_{2,3,3,11}$	0.00
$F_{3,3,3,11}$	-0.00	$F_{1,1,4,11}$	-0.00	$F_{1,2,4,11}$	0.00	$F_{2,2,4,11}$	-0.00
$F_{1,3,4,11}$	0.00	$F_{2,3,4,11}$	0.00	$F_{3,3,4,11}$	-0.00	$F_{1,4,4,11}$	0.00
$F_{2,4,4,11}$	-0.00	$F_{3,4,4,11}$	-0.00	$F_{4,4,4,11}$	-0.02	$F_{1,1,5,11}$	0.00
$F_{1,2,5,11}$	0.00	$F_{2,2,5,11}$	-0.00	$F_{1,3,5,11}$	-0.00	$F_{2,3,5,11}$	-0.00
$F_{3,3,5,11}$	-0.00	$F_{1,4,5,11}$	-0.00	$F_{2,4,5,11}$	0.00	$F_{3,4,5,11}$	0.00
$F_{4,4,5,11}$	0.01	$F_{1,5,5,11}$	0.00	$F_{2,5,5,11}$	0.00	$F_{3,5,5,11}$	0.00
$F_{4,5,5,11}$	0.00	$F_{5,5,5,11}$	-0.01	$F_{1,1,6,11}$	-0.00	$F_{1,2,6,11}$	0.00
$F_{2,2,6,11}$	-0.00	$F_{1,3,6,11}$	-0.00	$F_{2,3,6,11}$	-0.00	$F_{3,3,6,11}$	0.00
$F_{1,4,6,11}$	-0.00	$F_{2,4,6,11}$	-0.00	$F_{3,4,6,11}$	0.00	$F_{4,4,6,11}$	0.00
$F_{1,5,6,11}$	-0.00	$F_{2,5,6,11}$	0.00	$F_{3,5,6,11}$	0.00	$F_{4,5,6,11}$	-0.00
$F_{5,5,6,11}$	-0.00	$F_{1,6,6,11}$	0.00	$F_{2,6,6,11}$	0.00	$F_{3,6,6,11}$	0.00
$F_{4,6,6,11}$	-0.00	$F_{5,6,6,11}$	-0.00	$F_{6,6,6,11}$	-0.00	$F_{1,1,7,11}$	0.00
$F_{1,2,7,11}$	0.00	$F_{2,2,7,11}$	0.00	$F_{1,3,7,11}$	-0.00	$F_{2,3,7,11}$	-0.00
$F_{3,3,7,11}$	0.00	$F_{1,4,7,11}$	-0.00	$F_{2,4,7,11}$	0.00	$F_{3,4,7,11}$	0.00
$F_{4,4,7,11}$	0.02	$F_{1,5,7,11}$	0.00	$F_{2,5,7,11}$	-0.00	$F_{3,5,7,11}$	-0.00
$F_{4,5,7,11}$	-0.01	$F_{5,5,7,11}$	-0.00	$F_{1,6,7,11}$	0.00	$F_{2,6,7,11}$	0.00
$F_{3,6,7,11}$	0.00	$F_{4,6,7,11}$	-0.01	$F_{5,6,7,11}$	0.00	$F_{6,6,7,11}$	-0.00
$F_{1,7,7,11}$	0.00	$F_{2,7,7,11}$	-0.00	$F_{3,7,7,11}$	-0.00	$F_{4,7,7,11}$	-0.03
$F_{5,7,7,11}$	0.01	$F_{6,7,7,11}$	0.01	$F_{7,7,7,11}$	0.16	$F_{1,1,8,11}$	-0.00
$F_{1,2,8,11}$	-0.00	$F_{2,2,8,11}$	0.00	$F_{1,3,8,11}$	0.00	$F_{2,3,8,11}$	0.00
$F_{3,3,8,11}$	0.00	$F_{1,4,8,11}$	0.01	$F_{2,4,8,11}$	-0.00	$F_{3,4,8,11}$	-0.01
$F_{4,4,8,11}$	-0.01	$F_{1,5,8,11}$	-0.00	$F_{2,5,8,11}$	-0.00	$F_{3,5,8,11}$	-0.00
$F_{4,5,8,11}$	0.00	$F_{5,5,8,11}$	0.01	$F_{1,6,8,11}$	-0.00	$F_{2,6,8,11}$	-0.00
$F_{3,6,8,11}$	-0.00	$F_{4,6,8,11}$	0.01	$F_{5,6,8,11}$	0.01	$F_{6,6,8,11}$	0.00
$F_{1,7,8,11}$	-0.00	$F_{2,7,8,11}$	0.00	$F_{3,7,8,11}$	0.00	$F_{4,7,8,11}$	0.00
$F_{5,7,8,11}$	0.01	$F_{6,7,8,11}$	-0.01	$F_{7,7,8,11}$	0.11	$F_{1,8,8,11}$	0.00
$F_{2,8,8,11}$	0.00	$F_{3,8,8,11}$	-0.00	$F_{4,8,8,11}$	-0.02	$F_{5,8,8,11}$	-0.01
$F_{6,8,8,11}$	-0.01	$F_{7,8,8,11}$	-0.09	$F_{8,8,8,11}$	0.13	$F_{1,1,9,11}$	-0.00
$F_{1,2,9,11}$	0.00	$F_{2,2,9,11}$	-0.00	$F_{1,3,9,11}$	-0.00	$F_{2,3,9,11}$	-0.00
$F_{3,3,9,11}$	0.00	$F_{1,4,9,11}$	0.00	$F_{2,4,9,11}$	-0.00	$F_{3,4,9,11}$	0.00
$F_{4,4,9,11}$	0.01	$F_{1,5,9,11}$	-0.00	$F_{2,5,9,11}$	0.00	$F_{3,5,9,11}$	0.00
$F_{4,5,9,11}$	0.00	$F_{5,5,9,11}$	-0.00	$F_{1,6,9,11}$	0.00	$F_{2,6,9,11}$	0.00
$F_{3,6,9,11}$	-0.00	$F_{4,6,9,11}$	0.00	$F_{5,6,9,11}$	-0.00	$F_{6,6,9,11}$	0.00
$F_{1,7,9,11}$	-0.00	$F_{2,7,9,11}$	0.00	$F_{3,7,9,11}$	-0.00	$F_{4,7,9,11}$	-0.00
$F_{5,7,9,11}$	-0.00	$F_{6,7,9,11}$	-0.00	$F_{7,7,9,11}$	0.14	$F_{1,8,9,11}$	-0.00
$F_{2,8,9,11}$	-0.00	$F_{3,8,9,11}$	-0.00	$F_{4,8,9,11}$	-0.00	$F_{5,8,9,11}$	-0.00
$F_{6,8,9,11}$	0.01	$F_{7,8,9,11}$	-0.17	$F_{8,8,9,11}$	-0.37	$F_{1,9,9,11}$	-0.00
$F_{2,9,9,11}$	0.00	$F_{3,9,9,11}$	0.00	$F_{4,9,9,11}$	0.00	$F_{5,9,9,11}$	-0.00
$F_{6,9,9,11}$	0.00	$F_{7,9,9,11}$	-0.18	$F_{8,9,9,11}$	-0.63	$F_{9,9,9,11}$	-0.38
$F_{1,1,10,11}$	-0.00	$F_{1,2,10,11}$	-0.00	$F_{2,2,10,11}$	-0.00	$F_{1,3,10,11}$	0.00
$F_{2,3,10,11}$	0.00	$F_{3,3,10,11}$	0.00	$F_{1,4,10,11}$	0.00	$F_{2,4,10,11}$	0.00
$F_{3,4,10,11}$	-0.00	$F_{4,4,10,11}$	0.00	$F_{1,5,10,11}$	0.00	$F_{2,5,10,11}$	-0.00
$F_{3,5,10,11}$	-0.00	$F_{4,5,10,11}$	0.00	$F_{5,5,10,11}$	0.00	$F_{1,6,10,11}$	-0.00
$F_{2,6,10,11}$	-0.00	$F_{3,6,10,11}$	-0.00	$F_{4,6,10,11}$	0.01	$F_{5,6,10,11}$	-0.00
$F_{6,6,10,11}$	0.00	$F_{1,7,10,11}$	-0.00	$F_{2,7,10,11}$	0.00	$F_{3,7,10,11}$	-0.00
$F_{4,7,10,11}$	0.01	$F_{5,7,10,11}$	-0.00	$F_{6,7,10,11}$	-0.00	$F_{7,7,10,11}$	-0.14
$F_{1,8,10,11}$	-0.00	$F_{2,8,10,11}$	0.00	$F_{3,8,10,11}$	-0.00	$F_{4,8,10,11}$	-0.00
$F_{5,8,10,11}$	-0.01	$F_{6,8,10,11}$	0.00	$F_{7,8,10,11}$	-0.11	$F_{8,8,10,11}$	0.10
$F_{1,9,10,11}$	0.00	$F_{2,9,10,11}$	-0.00	$F_{3,9,10,11}$	0.00	$F_{4,9,10,11}$	-0.01
$F_{5,9,10,11}$	0.00	$F_{6,9,10,11}$	0.00	$F_{7,9,10,11}$	-0.13	$F_{8,9,10,11}$	0.18
$F_{9,9,10,11}$	0.18	$F_{1,10,10,11}$	0.00	$F_{2,10,10,11}$	0.00	$F_{3,10,10,11}$	0.00
$F_{4,10,10,11}$	-0.01	$F_{5,10,10,11}$	-0.00	$F_{6,10,10,11}$	-0.01	$F_{7,10,10,11}$	0.13
$F_{8,10,10,11}$	0.12	$F_{9,10,10,11}$	0.14	$F_{10,10,10,11}$	-0.12	$F_{1,1,11,11}$	0.00
$F_{1,2,11,11}$	-0.00	$F_{2,2,11,11}$	-0.00	$F_{1,3,11,11}$	-0.00	$F_{2,3,11,11}$	-0.00
$F_{3,3,11,11}$	-0.00	$F_{1,4,11,11}$	-0.00	$F_{2,4,11,11}$	0.00	$F_{3,4,11,11}$	0.00
$F_{4,4,11,11}$	-0.00	$F_{1,5,11,11}$	0.00	$F_{2,5,11,11}$	0.00	$F_{3,5,11,11}$	0.00
$F_{4,5,11,11}$	-0.00	$F_{5,5,11,11}$	-0.00	$F_{1,6,11,11}$	-0.00	$F_{2,6,11,11}$	0.00
$F_{3,6,11,11}$	0.00	$F_{4,6,11,11}$	-0.00	$F_{5,6,11,11}$	-0.00	$F_{6,6,11,11}$	0.00
$F_{1,7,11,11}$	0.00	$F_{2,7,11,11}$	-0.00	$F_{3,7,11,11}$	-0.00	$F_{4,7,11,11}$	0.01
$F_{5,7,11,11}$	-0.00	$F_{6,7,11,11}$	0.01	$F_{7,7,11,11}$	-0.12	$F_{1,8,11,11}$	-0.00
$F_{2,8,11,11}$	-0.00	$F_{3,8,11,11}$	0.00	$F_{4,8,11,11}$	0.02	$F_{5,8,11,11}$	0.01
$F_{6,8,11,11}$	0.00	$F_{7,8,11,11}$	0.08	$F_{8,8,11,11}$	-0.12	$F_{1,9,11,11}$	0.00

Table S22: The CcCR Force Constants for HSSH (cont.)

$F_{2,9,11,11}$	0.00	$F_{3,9,11,11}$	0.00	$F_{4,9,11,11}$	-0.00	$F_{5,9,11,11}$	0.00
$F_{6,9,11,11}$	-0.01	$F_{7,9,11,11}$	0.18	$F_{8,9,11,11}$	0.38	$F_{9,9,11,11}$	0.63
$F_{1,10,11,11}$	0.00	$F_{2,10,11,11}$	0.00	$F_{3,10,11,11}$	0.00	$F_{4,10,11,11}$	0.00
$F_{5,10,11,11}$	0.01	$F_{6,10,11,11}$	-0.00	$F_{7,10,11,11}$	0.11	$F_{8,10,11,11}$	-0.10
$F_{9,10,11,11}$	-0.18	$F_{10,10,11,11}$	-0.12	$F_{1,11,11,11}$	0.00	$F_{2,11,11,11}$	0.00
$F_{3,11,11,11}$	0.00	$F_{4,11,11,11}$	-0.01	$F_{5,11,11,11}$	-0.00	$F_{6,11,11,11}$	-0.00
$F_{7,11,11,11}$	-0.08	$F_{8,11,11,11}$	0.12	$F_{9,11,11,11}$	-0.38	$F_{10,11,11,11}$	0.09
$F_{11,11,11,11}$	-0.12	$F_{1,1,1,12}$	-0.00	$F_{1,1,2,12}$	-0.00	$F_{1,2,2,12}$	-0.00
$F_{2,2,2,12}$	-0.00	$F_{1,1,3,12}$	-0.00	$F_{1,2,3,12}$	0.00	$F_{2,2,3,12}$	-0.00
$F_{1,3,3,12}$	0.00	$F_{2,3,3,12}$	-0.00	$F_{3,3,3,12}$	0.00	$F_{1,1,4,12}$	-0.00
$F_{1,2,4,12}$	0.00	$F_{2,2,4,12}$	0.00	$F_{1,3,4,12}$	0.00	$F_{2,3,4,12}$	-0.00
$F_{3,3,4,12}$	-0.00	$F_{1,4,4,12}$	0.00	$F_{2,4,4,12}$	0.00	$F_{3,4,4,12}$	-0.00
$F_{4,4,4,12}$	-0.03	$F_{1,1,5,12}$	0.00	$F_{1,2,5,12}$	0.00	$F_{2,2,5,12}$	-0.00
$F_{1,3,5,12}$	0.00	$F_{2,3,5,12}$	0.00	$F_{3,3,5,12}$	0.00	$F_{1,4,5,12}$	-0.00
$F_{2,4,5,12}$	-0.00	$F_{3,4,5,12}$	0.00	$F_{4,4,5,12}$	-0.00	$F_{1,5,5,12}$	-0.00
$F_{2,5,5,12}$	0.00	$F_{3,5,5,12}$	-0.00	$F_{4,5,5,12}$	0.00	$F_{5,5,5,12}$	-0.00
$F_{1,1,6,12}$	0.00	$F_{1,2,6,12}$	0.00	$F_{2,2,6,12}$	0.00	$F_{1,3,6,12}$	-0.00
$F_{2,3,6,12}$	0.00	$F_{3,3,6,12}$	-0.00	$F_{1,4,6,12}$	-0.00	$F_{2,4,6,12}$	-0.00
$F_{3,4,6,12}$	0.00	$F_{4,4,6,12}$	0.00	$F_{1,5,6,12}$	-0.00	$F_{2,5,6,12}$	-0.00
$F_{3,5,6,12}$	-0.00	$F_{4,5,6,12}$	-0.00	$F_{5,5,6,12}$	-0.00	$F_{1,6,6,12}$	0.00
$F_{2,6,6,12}$	-0.00	$F_{3,6,6,12}$	-0.00	$F_{4,6,6,12}$	0.01	$F_{5,6,6,12}$	0.00
$F_{6,6,6,12}$	-0.00	$F_{1,1,7,12}$	0.00	$F_{1,2,7,12}$	0.00	$F_{2,2,7,12}$	-0.00
$F_{1,3,7,12}$	0.00	$F_{2,3,7,12}$	-0.00	$F_{3,3,7,12}$	0.00	$F_{1,4,7,12}$	-0.00
$F_{2,4,7,12}$	-0.00	$F_{3,4,7,12}$	-0.00	$F_{4,4,7,12}$	0.02	$F_{1,5,7,12}$	-0.00
$F_{2,5,7,12}$	0.01	$F_{3,5,7,12}$	0.00	$F_{4,5,7,12}$	-0.00	$F_{5,5,7,12}$	-0.01
$F_{1,6,7,12}$	-0.00	$F_{2,6,7,12}$	0.00	$F_{3,6,7,12}$	-0.00	$F_{4,6,7,12}$	-0.00
$F_{5,6,7,12}$	0.00	$F_{6,6,7,12}$	-0.00	$F_{1,7,7,12}$	0.00	$F_{2,7,7,12}$	0.00
$F_{3,7,7,12}$	-0.00	$F_{4,7,7,12}$	-0.03	$F_{5,7,7,12}$	0.00	$F_{6,7,7,12}$	0.01
$F_{7,7,7,12}$	0.16	$F_{1,1,8,12}$	-0.00	$F_{1,2,8,12}$	0.00	$F_{2,2,8,12}$	-0.00
$F_{1,3,8,12}$	-0.00	$F_{2,3,8,12}$	-0.00	$F_{3,3,8,12}$	-0.00	$F_{1,4,8,12}$	0.00
$F_{2,4,8,12}$	0.00	$F_{3,4,8,12}$	0.00	$F_{4,4,8,12}$	0.01	$F_{1,5,8,12}$	-0.00
$F_{2,5,8,12}$	0.00	$F_{3,5,8,12}$	0.00	$F_{4,5,8,12}$	0.00	$F_{5,5,8,12}$	-0.00
$F_{1,6,8,12}$	0.00	$F_{2,6,8,12}$	0.00	$F_{3,6,8,12}$	0.00	$F_{4,6,8,12}$	-0.00
$F_{5,6,8,12}$	-0.00	$F_{6,6,8,12}$	-0.00	$F_{1,7,8,12}$	-0.00	$F_{2,7,8,12}$	-0.00
$F_{3,7,8,12}$	0.00	$F_{4,7,8,12}$	-0.00	$F_{5,7,8,12}$	-0.00	$F_{6,7,8,12}$	-0.00
$F_{7,7,8,12}$	0.14	$F_{1,8,8,12}$	-0.00	$F_{2,8,8,12}$	-0.00	$F_{3,8,8,12}$	-0.00
$F_{4,8,8,12}$	-0.00	$F_{5,8,8,12}$	0.00	$F_{6,8,8,12}$	0.01	$F_{7,8,8,12}$	-0.17
$F_{8,8,8,12}$	-0.38	$F_{1,1,9,12}$	0.00	$F_{1,2,9,12}$	-0.00	$F_{2,2,9,12}$	0.00
$F_{1,3,9,12}$	-0.00	$F_{2,3,9,12}$	0.00	$F_{3,3,9,12}$	-0.00	$F_{1,4,9,12}$	0.00
$F_{2,4,9,12}$	0.00	$F_{3,4,9,12}$	0.00	$F_{4,4,9,12}$	0.00	$F_{1,5,9,12}$	0.00
$F_{2,5,9,12}$	-0.00	$F_{3,5,9,12}$	-0.00	$F_{4,5,9,12}$	0.00	$F_{5,5,9,12}$	-0.00
$F_{1,6,9,12}$	0.00	$F_{2,6,9,12}$	0.00	$F_{3,6,9,12}$	0.00	$F_{4,6,9,12}$	-0.01
$F_{5,6,9,12}$	0.00	$F_{6,6,9,12}$	0.00	$F_{1,7,9,12}$	-0.00	$F_{2,7,9,12}$	0.00
$F_{3,7,9,12}$	-0.00	$F_{4,7,9,12}$	0.00	$F_{5,7,9,12}$	-0.01	$F_{6,7,9,12}$	0.01
$F_{7,7,9,12}$	0.12	$F_{1,8,9,12}$	-0.00	$F_{2,8,9,12}$	0.00	$F_{3,8,9,12}$	0.00
$F_{4,8,9,12}$	-0.00	$F_{5,8,9,12}$	0.00	$F_{6,8,9,12}$	-0.00	$F_{7,8,9,12}$	-0.17
$F_{8,8,9,12}$	-0.63	$F_{1,9,9,12}$	0.00	$F_{2,9,9,12}$	-0.00	$F_{3,9,9,12}$	-0.00
$F_{4,9,9,12}$	-0.00	$F_{5,9,9,12}$	-0.00	$F_{6,9,9,12}$	-0.01	$F_{7,9,9,12}$	-0.10
$F_{8,9,9,12}$	-0.37	$F_{9,9,9,12}$	0.14	$F_{1,1,10,12}$	0.00	$F_{1,2,10,12}$	-0.00
$F_{2,2,10,12}$	0.00	$F_{1,3,10,12}$	-0.00	$F_{2,3,10,12}$	0.00	$F_{3,3,10,12}$	-0.00
$F_{1,4,10,12}$	0.00	$F_{2,4,10,12}$	0.00	$F_{3,4,10,12}$	0.00	$F_{4,4,10,12}$	0.00
$F_{1,5,10,12}$	0.00	$F_{2,5,10,12}$	-0.00	$F_{3,5,10,12}$	-0.00	$F_{4,5,10,12}$	0.01
$F_{5,5,10,12}$	0.00	$F_{1,6,10,12}$	0.00	$F_{2,6,10,12}$	-0.00	$F_{3,6,10,12}$	0.00
$F_{4,6,10,12}$	0.00	$F_{5,6,10,12}$	-0.00	$F_{6,6,10,12}$	-0.00	$F_{1,7,10,12}$	-0.00
$F_{2,7,10,12}$	-0.00	$F_{3,7,10,12}$	0.00	$F_{4,7,10,12}$	0.01	$F_{5,7,10,12}$	-0.00
$F_{6,7,10,12}$	-0.00	$F_{7,7,10,12}$	-0.14	$F_{1,8,10,12}$	-0.00	$F_{2,8,10,12}$	0.00
$F_{3,8,10,12}$	0.00	$F_{4,8,10,12}$	-0.01	$F_{5,8,10,12}$	0.00	$F_{6,8,10,12}$	0.00
$F_{7,8,10,12}$	-0.13	$F_{8,8,10,12}$	0.18	$F_{1,9,10,12}$	0.00	$F_{2,9,10,12}$	-0.00
$F_{3,9,10,12}$	0.00	$F_{4,9,10,12}$	-0.00	$F_{5,9,10,12}$	0.01	$F_{6,9,10,12}$	-0.01
$F_{7,9,10,12}$	-0.12	$F_{8,9,10,12}$	0.17	$F_{9,9,10,12}$	0.10	$F_{1,10,10,12}$	-0.00
$F_{2,10,10,12}$	-0.00	$F_{3,10,10,12}$	-0.00	$F_{4,10,10,12}$	-0.01	$F_{5,10,10,12}$	-0.00
$F_{6,10,10,12}$	0.00	$F_{7,10,10,12}$	0.13	$F_{8,10,10,12}$	0.14	$F_{9,10,10,12}$	0.12
$F_{10,10,10,12}$	-0.11	$F_{1,1,11,12}$	0.00	$F_{1,2,11,12}$	-0.00	$F_{2,2,11,12}$	0.00
$F_{1,3,11,12}$	0.00	$F_{2,3,11,12}$	0.00	$F_{3,3,11,12}$	0.00	$F_{1,4,11,12}$	-0.00
$F_{2,4,11,12}$	0.00	$F_{3,4,11,12}$	-0.00	$F_{4,4,11,12}$	-0.01	$F_{1,5,11,12}$	0.00
$F_{2,5,11,12}$	-0.00	$F_{3,5,11,12}$	-0.00	$F_{4,5,11,12}$	-0.00	$F_{5,5,11,12}$	0.00
$F_{1,6,11,12}$	-0.00	$F_{2,6,11,12}$	-0.00	$F_{3,6,11,12}$	-0.00	$F_{4,6,11,12}$	0.00
$F_{5,6,11,12}$	0.00	$F_{6,6,11,12}$	0.00	$F_{1,7,11,12}$	0.00	$F_{2,7,11,12}$	-0.00
$F_{3,7,11,12}$	-0.00	$F_{4,7,11,12}$	0.01	$F_{5,7,11,12}$	0.00	$F_{6,7,11,12}$	0.00
$F_{7,7,11,12}$	-0.14	$F_{1,8,11,12}$	0.00	$F_{2,8,11,12}$	0.00	$F_{3,8,11,12}$	0.00
$F_{4,8,11,12}$	0.00	$F_{5,8,11,12}$	-0.00	$F_{6,8,11,12}$	-0.00	$F_{7,8,11,12}$	0.18
$F_{8,8,11,12}$	0.38	$F_{1,9,11,12}$	0.00	$F_{2,9,11,12}$	-0.00	$F_{3,9,11,12}$	-0.00
$F_{4,9,11,12}$	-0.00	$F_{5,9,11,12}$	0.00	$F_{6,9,11,12}$	-0.00	$F_{7,9,11,12}$	0.18
$F_{8,9,11,12}$	0.62	$F_{9,9,11,12}$	0.38	$F_{1,10,11,12}$	0.00	$F_{2,10,11,12}$	0.00

Table S23: The CcCR Force Constants for HSSH (cont.)

$F_{3,10,11,12}$	0.00	$F_{4,10,11,12}$	0.00	$F_{5,10,11,12}$	-0.00	$F_{6,10,11,12}$	-0.00
$F_{7,10,11,12}$	0.14	$F_{8,10,11,12}$	-0.18	$F_{9,10,11,12}$	-0.18	$F_{10,10,11,12}$	-0.14
$F_{1,11,11,12}$	-0.00	$F_{2,11,11,12}$	-0.00	$F_{3,11,11,12}$	-0.00	$F_{4,11,11,12}$	0.00
$F_{5,11,11,12}$	0.00	$F_{6,11,11,12}$	0.00	$F_{7,11,11,12}$	-0.18	$F_{8,11,11,12}$	-0.38
$F_{9,11,11,12}$	-0.62	$F_{10,11,11,12}$	0.18	$F_{11,11,11,12}$	0.38	$F_{1,1,12,12}$	0.00
$F_{1,2,12,12}$	0.00	$F_{2,2,12,12}$	-0.00	$F_{1,3,12,12}$	0.00	$F_{2,3,12,12}$	-0.00
$F_{3,3,12,12}$	0.00	$F_{1,4,12,12}$	-0.00	$F_{2,4,12,12}$	0.00	$F_{3,4,12,12}$	-0.00
$F_{4,4,12,12}$	-0.01	$F_{1,5,12,12}$	0.00	$F_{2,5,12,12}$	0.00	$F_{3,5,12,12}$	0.00
$F_{4,5,12,12}$	-0.00	$F_{5,5,12,12}$	0.00	$F_{1,6,12,12}$	-0.00	$F_{2,6,12,12}$	-0.00
$F_{3,6,12,12}$	-0.00	$F_{4,6,12,12}$	0.00	$F_{5,6,12,12}$	-0.00	$F_{6,6,12,12}$	-0.00
$F_{1,7,12,12}$	0.00	$F_{2,7,12,12}$	-0.00	$F_{3,7,12,12}$	0.00	$F_{4,7,12,12}$	0.00
$F_{5,7,12,12}$	0.01	$F_{6,7,12,12}$	-0.01	$F_{7,7,12,12}$	-0.12	$F_{1,8,12,12}$	-0.00
$F_{2,8,12,12}$	-0.00	$F_{3,8,12,12}$	-0.00	$F_{4,8,12,12}$	0.00	$F_{5,8,12,12}$	-0.00
$F_{6,8,12,12}$	0.01	$F_{7,8,12,12}$	0.18	$F_{8,8,12,12}$	0.62	$F_{1,9,12,12}$	-0.00
$F_{2,9,12,12}$	-0.00	$F_{3,9,12,12}$	-0.00	$F_{4,9,12,12}$	0.01	$F_{5,9,12,12}$	0.00
$F_{6,9,12,12}$	0.01	$F_{7,9,12,12}$	0.09	$F_{8,9,12,12}$	0.37	$F_{9,9,12,12}$	-0.13
$F_{1,10,12,12}$	-0.00	$F_{2,10,12,12}$	0.00	$F_{3,10,12,12}$	-0.00	$F_{4,10,12,12}$	0.00
$F_{5,10,12,12}$	-0.01	$F_{6,10,12,12}$	0.01	$F_{7,10,12,12}$	0.12	$F_{8,10,12,12}$	-0.18
$F_{9,10,12,12}$	-0.10	$F_{10,10,12,12}$	-0.12	$F_{1,11,12,12}$	-0.00	$F_{2,11,12,12}$	0.00
$F_{3,11,12,12}$	0.00	$F_{4,11,12,12}$	0.00	$F_{5,11,12,12}$	-0.00	$F_{6,11,12,12}$	-0.00
$F_{7,11,12,12}$	-0.18	$F_{8,11,12,12}$	-0.62	$F_{9,11,12,12}$	-0.37	$F_{10,11,12,12}$	0.18
$F_{11,11,12,12}$	0.62	$F_{1,12,12,12}$	0.00	$F_{2,12,12,12}$	0.00	$F_{3,12,12,12}$	0.00
$F_{4,12,12,12}$	-0.01	$F_{5,12,12,12}$	-0.00	$F_{6,12,12,12}$	-0.01	$F_{7,12,12,12}$	-0.08
$F_{8,12,12,12}$	-0.37	$F_{9,12,12,12}$	0.12	$F_{10,12,12,12}$	0.09	$F_{11,12,12,12}$	0.37
$F_{12,12,12,12}$	-0.12						

Table S24: The CcCR Vibrational Frequencies (cm^{-1}) for the Isotopologues of HOOH

Mode	$H^{18}OOH$	DOOH	DOOD	$D^{18}O^{18}OD$
ω_1	3805.6	3805.6	2774.7	2756.4
ω_2	3792.8	2773.1	2771.3	2753.9
ω_3	1438.5	1392.0	1056.3	1048.6
ω_4	1329.8	1017.1	986.5	977.0
ω_5	889.1	913.7	912.7	860.9
ω_6	379.5	333.3	278.2	276.3
ν_1	3609.7	3609.2	2670.1	2653.1
ν_2	3598.1	2669.5	2668.1	2652.0
ν_3	1395.2	1375.0	1030.3	1023.2
ν_4	1275.6	986.9	953.1	945.0
ν_5	854.0	879.6	880.9	832.1
ν_6	314.2	282.6	242.7	241.4
ZPT	5717.7	5038.4	4333.5	4281.6

Table S25: The CcCR Geometrical Parameters and Spectroscopic Constants for the Isotopologues of HOOH

	Units	$H^{18}OOH$	DOOH	DOOD	$D^{18}O^{18}OH$
$R_e(H_1\text{-}O_1)$	Å	0.96198	0.96198	0.96198	0.96198
$R_e(O_1\text{-}O_2)$	Å	1.44803	1.44803	1.44803	1.44803
$R_e(O_2\text{-}H_2)$	Å	0.96198	0.96198	0.96198	0.96198
$\angle_e(O_1\text{-}H_1\text{-}O_2)$	°	100.15	100.15	100.15	100.15
$\angle_e(O_1\text{-}H_1\text{-}H_2)$	°	100.15	100.15	100.15	100.15
A_e	MHz	303372.3	214618.4	166303.9	163164.1
B_e	MHz	25260.8	25186.5	23592.5	21519.7
C_e	MHz	24500.5	23899.2	22466.1	20550.5
$R_0(H_1\text{-}O_1)$	Å	0.96232	0.96615	0.96248	0.96242
$R_0(O_1\text{-}O_2)$	Å	1.46144	1.46086	1.46001	1.45955
$R_0(O_2\text{-}H_2)$	Å	0.96218	0.95638	0.96248	0.96242
$\angle_0(O_1\text{-}H_1\text{-}O_2)$	°	100.06	99.92	100.02	100.03
$\angle_0(O_1\text{-}H_1\text{-}H_2)$	°	100.06	100.17	100.02	100.03
A_0	MHz	300658.7	213127.0	165402.4	162243.3
B_0	MHz	24975.6	24934.3	23402.6	21346.0
C_0	MHz	24073.8	23515.9	22119.0	20244.6
A_1	MHz	295067.1	210320.8	163191.4	160092.0
B_1	MHz	24939.6	24886.4	23347.2	21302.0
C_1	MHz	24071.7	23523.2	22105.1	20234.2
A_2	MHz	294991.1	209441.7	163165.0	160048.6
B_2	MHz	24942.3	24893.6	23342.3	21297.1
C_2	MHz	24073.3	23477.8	22103.4	20233.1
A_3	MHz	304196.9	214844.4	166783.4	163610.8
B_3	MHz	24803.7	24784.5	23271.2	21229.2
C_3	MHz	23999.5	23426.4	22167.4	20263.9
A_4	MHz	304871.3	215974.7	167278.3	164013.5
B_4	MHz	24910.9	24916.9	23462.9	21374.3
C_4	MHz	23846.0	23405.7	21924.9	20076.4
A_5	MHz	300056.7	212650.6	164940.0	161855.4
B_5	MHz	24680.1	24646.8	23118.6	21108.9
C_5	MHz	23773.8	23191.6	21822.2	19996.1
A_6	MHz	299342.1	212547.0	165253.7	161998.4
B_6	MHz	24982.7	24969.3	23473.0	21400.0
C_6	MHz	23849.2	23308.5	21916.9	20069.3
Δ_J	kHz	87.665	79.369	78.605	64.488
Δ_K	MHz	10.882	6.325	3.696	3.427
Δ_{JK}	kHz	1.057 (MHz)	921.464	641.912	566.125
δ_J	Hz	-25.078	1.513 (kHz)	-642.400	-322.349
δ_K	MHz	6.164	1.283	5.306	4.411
Φ_j	mHz	-7.464	-72.613	158.252	90.267
Φ_k	kHz	1.659	1.463	772.462 (Hz)	586.836 (Hz)
Φ_{jk}	Hz	64.402	226.725	89.392	62.262
Φ_{kj}	Hz	-175.364	-795.880	-417.549	-278.660
ϕ_j	mHz	894.134 (μHz)	85.821	19.711	13.179
ϕ_{jk}	Hz	95.243	-33.217	94.518	67.211
ϕ_k	kHz	-47.806	49.734	-26.123	-20.668

Table S26: The CcCR Vibrational Frequencies (cm^{-1}) for the Isotopologues of HOSH

Mode	$H^{18}OSH$	$HO^{34}SH$	DOSH	DOSD	$H^{18}O^{34}SH$
ω_1	3812.7	3825.5	2786.5	2786.5	3812.7
ω_2	2661.9	2659.5	2661.9	1912.7	2659.5
ω_3	1207.5	1210.2	1032.6	885.6	1206.8
ω_4	1030.7	1032.7	885.5	791.0	1029.8
ω_5	757.1	777.9	783.3	749.6	748.8
ω_6	478.2	479.6	397.1	350.7	478.0
ν_1	3617.2	3628.6	2682.3	2682.1	3617.2
ν_2	2546.7	2544.5	2546.2	1853.7	2544.5
ν_3	1171.7	1174.1	1008.2	865.2	1171.0
ν_4	1006.7	1008.4	864.5	774.3	1005.8
ν_5	736.9	756.3	768.7	737.3	729.2
ν_6	436.7	437.8	368.5	327.9	436.6
ZPT	4898.2	4916.4	4219.5	3696.7	4892.1

Table S27: The CcCR Geometrical Parameters and Spectroscopic Constants for the Isotopologues of HOSH

	Units	$H^{18}OSH$	$HO^{34}SH$	DOSH	DOSD	$H^{18}O^{34}SH$
$R_e(H_1\text{-}O)$	Å	0.96012	0.96012	0.9601	0.96012	0.96012
$R_e(O\text{-}S)$	Å	1.66051	1.66051	1.6605	1.66051	1.66051
$R_e(S\text{-}H_2)$	Å	1.34273	1.34273	1.3427	1.34273	1.34273
$\angle_e(O\text{-}H_1\text{-}S)$	°	107.21	107.21	107.2	107.21	107.21
$\angle_e(S\text{-}O\text{-}H_2)$	°	98.45	98.45	98.4	98.45	98.45
A_e	MHz	202735.5	203009.3	160886.3	107317.0	202408.2
B_e	MHz	14405.9	15134.4	14256.6	13970.6	14120.2
C_e	MHz	14037.2	14723.1	14147.5	13294.0	13764.1
$R_0(H_1\text{-}O)$	Å	0.95807	0.95803	0.96175	0.95875	0.95805
$R_0(O\text{-}S)$	Å	1.66903	1.66919	1.66873	1.66840	1.66897
$R_0(S\text{-}H_2)$	Å	1.35220	1.35226	1.34914	1.34992	1.35219
$\angle_0(O\text{-}H_1\text{-}S)$	°	107.51	107.51	107.32	107.40	107.51
$\angle_0(S\text{-}O\text{-}H_2)$	°	98.51	98.51	98.56	98.46	98.51
A_0	MHz	201383.8	201658.1	159997.7	106836.0	201056.8
B_0	MHz	14295.3	15016.5	14139.0	13879.6	14012.2
C_0	MHz	13913.7	14591.1	14044.4	13187.5	13643.8
A_1	MHz	198955.8	199241.9	157995.9	105943.8	198636.4
B_1	MHz	14274.3	14992.7	14108.6	13850.0	13991.9
C_1	MHz	13904.2	14580.0	14028.1	13172.6	13634.6
A_2	MHz	197367.4	197622.3	157472.0	105310.3	197044.2
B_2	MHz	14313.0	15035.3	14155.4	13886.0	14029.9
C_2	MHz	13914.3	14590.9	14044.0	13173.5	13645.0
A_3	MHz	204379.6	204672.2	161452.2	108096.8	204043.2
B_3	MHz	14256.7	14975.4	14126.0	13855.6	13974.5
C_3	MHz	13913.3	14591.7	13988.6	13200.4	13642.9
A_4	MHz	203693.4	203968.7	162636.1	106801.3	203356.3
B_4	MHz	14280.5	15002.3	14113.6	13768.9	13997.2
C_4	MHz	13854.7	14529.8	14059.9	13146.7	13586.1
A_5	MHz	201193.8	201463.2	159727.0	107600.3	200869.8
B_5	MHz	14176.4	14889.1	14012.9	13876.9	13897.0
C_5	MHz	13797.4	14465.2	13925.1	13075.6	13531.0
A_6	MHz	200009.2	200278.2	158925.6	106301.8	199688.2
B_6	MHz	14252.8	14972.3	14101.0	13860.6	13970.2
C_6	MHz	13848.2	14521.7	13996.2	13140.4	13579.6
Δ_J	kHz	20.814	22.740	19.434	17.060	20.059
Δ_K	MHz	5.472	5.474	3.848	1.514	5.453
Δ_{JK}	kHz	345.443	373.986	291.004	254.383	334.341
δ_J	Hz	562.175	645.367	154.134	828.194	532.699
δ_K	MHz	-0.739	-0.790	-4.578	-0.567	-0.707
Φ_j	mHz	-11.755	-13.644	-9.287	-6.770	-11.073
Φ_k	Hz	431.584	429.231	409.595	64.083	427.003
Φ_{jk}	Hz	-7.034	-8.407	-10.995	-9.819	-6.485
Φ_{kj}	Hz	49.217	55.355	40.910	37.261	46.653
ϕ_j	μHz	387.635	378.666	-26.850	203.398	359.204
ϕ_{jk}	mHz	-428.852	-624.312	-8.519 (Hz)	-1.905 (Hz)	-364.575
ϕ_k	kHz	-7.280	-7.642	-400.589	-2.523	-6.946

Table S28: The CcCR Vibrational Frequencies (cm^{-1}) for the Isotopologues of HSSH

Mode	$H^{34}SSH$	DSSH	DSSD	$D^{34}S^{34}SD$
ω_1	2682.4	2681.8	1927.4	1923.9
ω_2	2678.8	1926.5	1925.6	1922.2
ω_3	907.8	907.5	660.3	658.1
ω_4	906.6	656.1	651.3	650.2
ω_5	522.1	529.7	529.5	513.7
ω_6	443.4	386.8	319.8	319.1
ν_1	2568.9	2567.3	1869.0	1865.7
ν_2	2564.8	1868.3	1866.8	1863.5
ν_3	886.8	884.5	647.6	645.6
ν_4	883.4	642.4	646.4	645.0
ν_5	510.6	518.8	519.0	503.9
ν_6	405.6	357.7	299.7	299.1
ZPT	4014.4	3500.9	2977.1	2964.0

Table S29: The CcCR Geometrical Parameters and Spectroscopic Constants for the Isotopologues of HSSH

	Units	$H^{34}SSH$	DSSH	DSSD	$D^{34}S^{34}SH$
$R_e(H_1\text{-}S_1)$	Å	1.34066	1.34066	1.34066	1.34066
$R_e(S_1\text{-}S_2)$	Å	2.05016	2.05016	2.05016	2.05016
$R_e(S_2\text{-}H_2)$	Å	2.60447	2.60447	2.60447	2.60447
$\angle_e(S_1\text{-}H_1\text{-}S_2)$	°	98.19	98.19	98.19	98.19
$\angle_e(S_1\text{-}H_1\text{-}H_2)$	°	97.36	97.36	97.36	97.36
$\angle_e(S_1\text{-}S_2\text{-}H_1)$	°	30.63	30.63	30.63	30.63
A_e	MHz	147710.1	101104.7	76807.7	76473.5
B_e	MHz	6830.8	6872.4	6586.1	6244.7
C_e	MHz	6829.3	6727.6	6584.3	6243.8
$R_0(H_1\text{-}S_1)$	Å	1.34588	1.34676	1.34457	1.34454
$R_0(S_1\text{-}S_2)$	Å	2.05876	2.05830	2.05772	2.05758
$R_0(S_2\text{-}H_2)$	Å	2.61586	2.61411	2.61355	2.61345
$\angle_0(S_1\text{-}H_1\text{-}S_2)$	°	98.23	98.15	98.19	98.20
$\angle_0(S_1\text{-}H_1\text{-}H_2)$	°	97.46	97.39	97.43	97.43
$\angle_0(S_1\text{-}S_2\text{-}H_1)$	°	30.61	30.54	30.61	30.61
A_0	MHz	146591.3	100493.2	76402.5	76069.3
B_0	MHz	6811.4	6831.0	6591.1	6264.8
C_0	MHz	6754.2	6683.0	6503.6	6152.8
A_1	MHz	144529.4	99522.0	75650.1	75319.7
B_1	MHz	6816.8	6835.0	6592.5	6266.4
C_1	MHz	6760.9	6690.7	6503.9	6153.4
A_2	MHz	144479.6	99162.5	75621.4	75291.9
B_2	MHz	6818.0	6834.9	6592.9	6266.8
C_2	MHz	6760.8	6681.5	6504.2	6153.7
A_3	MHz	148835.0	101151.1	77173.2	76830.5
B_3	MHz	6797.9	6805.8	6575.2	6249.8
C_3	MHz	6741.2	6671.9	6499.0	6147.8
A_4	MHz	147207.7	101469.1	76743.4	76408.6
B_4	MHz	6786.7	6829.8	6589.6	6262.3
C_4	MHz	6731.1	6665.1	6487.9	6137.8
A_5	MHz	146523.7	100439.3	76357.8	76027.6
B_5	MHz	6772.1	6791.5	6552.0	6229.1
C_5	MHz	6715.9	6642.9	6464.7	6117.1
A_6	MHz	145735.0	99992.6	76058.4	75729.4
B_6	MHz	6783.7	6807.1	6567.4	6242.3
C_6	MHz	6720.2	6655.6	6487.2	6137.0
Δ_J	kHz	5.058	4.736	4.529	4.079
Δ_K	MHz	2.264	1.177	0.602	0.598
Δ_{JK}	kHz	81.461	79.432	62.643	57.252
δ_J	Hz	-7.487	97.988	16.875	13.766
δ_K	MHz	13.766	-0.153	21.741	27.986
Φ_j	mHz	-1.117	-1.297	-723.733 (μHz)	-672.823 (μHz)
Φ_k	kHz	1.515	39.617	-11.978 (Hz)	-17.027 (Hz)
Φ_{jk}	Hz	610.693	-517.843 (mHz)	-11.773	-13.693
Φ_{kj}	kHz	-2.032	3.581 (Hz)	40.319 (Hz)	46.657 (Hz)
ϕ_j	μHz	4.575	29.504	-13.924	-11.863 (μHz)
ϕ_{jk}	Hz	283.132	38.949 (mHz)	-821.738	-1.391 (kHz)
ϕ_k	GHz	-0.180	-762.307 (Hz)	-0.570	-1.489
